# Capillary force on an ‘inert’ colloid: a physical analogy to dielectrophoresis†

**DOI:** 10.1039/d0sm02143a

**Published:** 2021-02-16

**Authors:** Joseph M. Barakat, Todd M. Squires

**Affiliations:** Department of Chemical Engineering, University of California, Santa Barbara California 93106 USA squires@engineering.ucsb.edu

## Abstract

“Inert” colloids are μm-scale particles that create no distortion when trapped at a planar fluid–fluid interface. When placed in a curved interface, however, such colloids can create interfacial distortions of quadrupolar symmetry – so-called “induced capillary quadrupoles.” The present work explores the analogy between capillary quadrupoles and electric dipoles, and the forces exerted on them by a symmetry-breaking gradient. In doing so, we weigh in on an outstanding debate as to whether a curvature gradient can induce a capillary force on an inert colloid. We argue that this force exists, for the opposite would imply that all dielectrophoretic forces vanish in two dimensions (2D). We justify our claim by solving 2D Laplace problems of electrostatics and capillary statics involving a single particle placed within a large circular shell with an imposed gradient. We show that the static boundary condition on the outer shell must be considered when applying the principle of virtual work to compute the force on the particle, as verified by a direct calculation of this force through integration of the particle stresses. Our investigation highlights some of the subtleties that emerge in virtual work calculations of capillary statics and electrostatics, thereby clarifying and extending previous results in the field. The broader implication of our results is that inert particles – including particles with planar, pinned contact lines and equilibrium contact angles – interact through interparticle capillary forces that scale quadratically with the deviatoric curvature of the host interface, contrary to recent claims made in the literature.

## Abstract 2

The present work explores the analogy between dielectrophoresis of non-polar particles and the motion of interface-trapped, “inert” colloids – particles with planar contact lines or equilibrium contact angles – in a curvature gradient.

## Graphical abstract

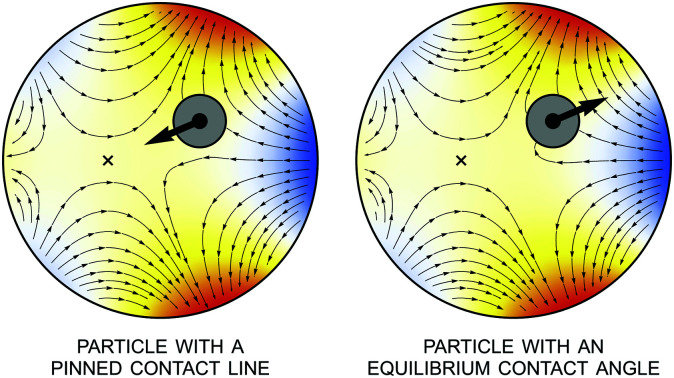

## 1 Introduction

Colloidal (μm-scale) particles readily adsorb to fluid–fluid interfaces and interact over large (mm-scale) distances *via* capillary forces.^1,2^ These interactions can be exploited to drive colloidal self-assembly at interfaces,^3–9^ which offers a promising route for the design of advanced two-dimensional (2D) materials.^2,10,11^ Interparticle capillary forces generically result from interfacial deformations due to the presence of the particles. Since colloidal particles have negligible buoyant weight, they can deform an interface only by virtue of their shape^12–15^ or wetting properties.^3–5^ Thus, colloidal capillary interactions are distinct from buoyancy-driven aggregation of mm-scale particles (*i.e.*, the “Cheerios effect”).^16–18^

The present work addresses “inert” colloids, *i.e.*, colloidal particles that produce no distortion in *planar* interfaces, but do deform *curved* interfaces. The wetting condition at the particle boundary interferes with the background curvature of the host interface, inducing an interfacial distortion of quadrupolar symmetry.^19^ Recently, there has been some disagreement^19–28^ as to whether such “induced capillary quadrupoles” experience a force when embedded in a gradient of interfacial curvature. Early theoretical work by Würger^19^ and others^20–22,29^ predicted that inert colloids on a curved interface would attract due to their induced quadrupoles, with a capillary force that scales quadratically with the deviatoric curvature of the host interface. However, later calculations by Sharifi-Mood *et al.*^23,24^ reported that the same force vanishes up to quadratic order in the curvature. Although both studies employed the principle of virtual work to compute the capillary force, they differ in their treatment of the outer boundary enclosing the particles. Confounding this issue is the practical challenge of measuring such forces, which are typically obscured by interfacial deformations due to particle roughness.^23,24^ The discrepancy between the two theoretical results, combined with the relative dearth of experimental evidence to support either view, has incited a spirited debate^25–28^ with no clear consensus on the “correct” approach.

Further insight might be gained by exploiting the analogy between capillary statics and 2D electrostatics. While this mathematical analogy is widely acknowledged in the literature,^2,29–31^ some of its important physical implications have received less attention. We propose that the capillary force on a quadrupole induced by a background curvature is akin to the dielectrophoretic force on an induced dipole in an inhomogeneous electric field. Dielectrophoretic forces are well known and well established.^32–39^ They can be computed directly, by integrating the Maxwell stress tensor over the particle boundary. Alternatively, they can be derived using the principle of virtual work, so long as the electrostatic condition on the outer enclosing boundary is taken into account.^40^ It stands to reason that consideration of the outer boundary is equally relevant to the calculation of capillary forces by virtual work arguments. Indeed, this issue was discussed explicitly by Domínguez *et al.*,^29^ who developed a useful stress tensor formulation for capillary statics based on the analogy to 2D electrostatics.

In the spirit of this analogy, we reexamine the force on an induced capillary quadrupole in an interface with an inhomogeneous, anisotropic curvature. Specifically, we show that particles with pinned contact lines or equilibrium contact angles in a curvature gradient are analogous to conducting or insulating particles in an electric field gradient. To resolve any ambiguities with the outer boundary, we consider a freely floating particle placed inside a large shell, and explicitly place sources on the outer shell that establish a gradient. We then compute the force on the particle using both the principle of virtual work and the stress tensor approach. In the electric problem, we find that neglecting the work done on the outer boundary leads to the erroneous conclusion that the dielectrophoretic force vanishes in 2D, whereas properly accounting for this work recovers the expected scaling between the force and the gradient of the squared electric field strength. Applying the same analysis to the analogous capillary problem yields a similar relationship between the force and the gradient of the squared deviatoric curvature, in agreement with earlier studies.^19–22,29^ Our results clarify and extend previous calculations in the field and suggest that inert colloids interact on curved interfaces. More broadly, our work highlights some of the issues that can arise when applying the principle of virtual work to compute interparticle forces in unbounded media.

## 2 Background

**Fig. 1:**
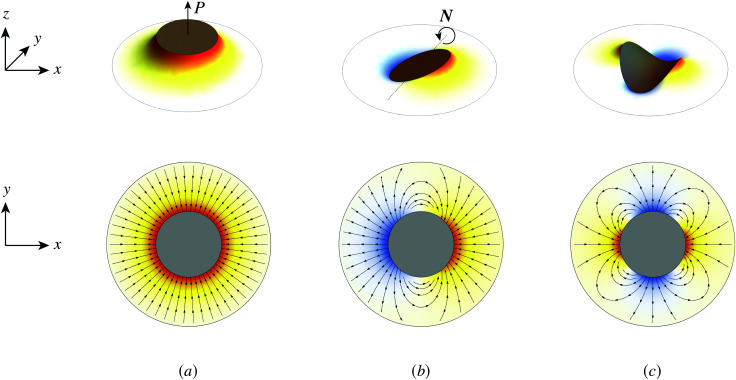
“Permanent” capillary multipoles sourced by a particle embedded in an initially planar fluid–fluid interface. “Hot” and “cold” colors represent, respectively, displacements of the interface above and below the undeformed plane. Gradient lines indicate the direction of the interface slope. (a) Monopole produced by a vertical displacement out of the plane (*e.g.*, due to the buoyant force *P* exerted on a light particle by a gravitational field). (b) Dipole produced by a rotation about the horizontal (*e.g.*, due to the magnetic torque ***N*** exerted on a paramagnetic particle by a magnetic field). (c) Quadrupole produced by a saddle undulation of the contact line (*e.g.*, due to particle shape, surface chemistry, or surface roughness).

Previous studies of capillary interactions between particles at fluid–fluid interfaces have largely focused on interfacial distortions like the ones shown in Fig. 1. By far the most well studied^16–18,41–47^ interactions are due to capillary monopoles (Fig. 1a). These interfacial deformations are produced by the action of a transverse force *P*, *e.g.*, the force of gravity acting on a heavy or light particle trapped at an interface. Two particles with overlapping capillary monopoles of “like sign” will feel a lateral force of attraction, as in the “Cheerios effect.”^16–18^ Similarly, a lateral torque ***N*** that rotates a particle out of the undeformed plane creates a capillary dipole (Fig. 1b). Davies *et al.*^9,48–51^ showed that such torques can be achieved, at least in theory, by magnetizing a paramagnetic particle *via* an orthogonal magnetic field.

Colloidal particles embedded in a fluid–fluid interface are typically too small to experience significant transverse forces or lateral torques, so their monopole and dipole moments are negligible.^52^ Thus, the lowest-order deformation mode produced by a colloid is a capillary quadrupole (Fig. 1c). Colloids may create their own quadrupoles by virtue of their anisotropic shape,^12–15,52,53^ surface chemistry,^3,4,54^ or surface roughness.^23,24,30,31,55–57^ We refer to such distortions as permanent quadrupoles, following the terminology of Domínguez *et al.*,^29^ because they persist even in the absence of a background curvature. In the presence of a curvature gradient, an embedded particle with a permanent quadrupole moment experiences a lateral force ***F*** and transverse torque *T*.^58^ Hu and Bush^59^ famously showed that this coupling enables the larva of the waterlily leaf beetle to climb water menisci by simply adopting an arched body posture, which creates a quadrupolar deformation. Importantly, the lateral force due to a permanent quadrupole has been shown (both theoretically^30^ and experimentally^23,24^) to scale linearly with the deviatoric curvature.

**Fig. 2:**
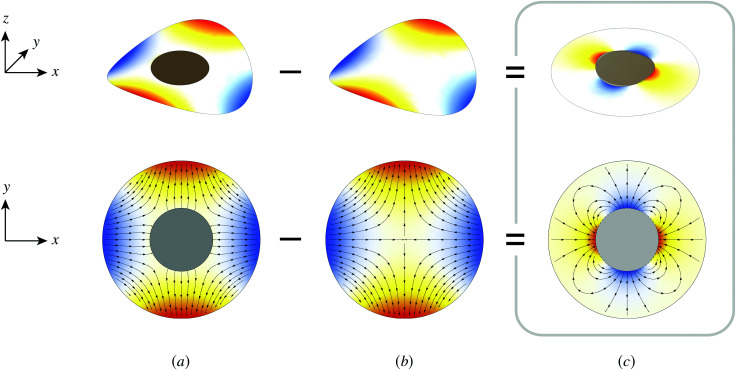
“Induced” capillary quadrupole emanating from a planar particle trapped at a curved fluid–fluid interface. The colors and gradient lines have the same meaning as in Fig. 1. (a) The presence of the particle distorts the surrounding interface. (b) In the particle's absence, the interface locally adopts a saddle shape, characteristic of a uniform deviatoric curvature. (c) Subtracting the particle-free interface from the distorted interface reveals the induced quadrupole.

Capillary quadrupole moments can also be *induced* by the mere presence of a colloidal particle in a background curvature (Fig. 2). Unlike permanent quadrupoles, induced quadrupoles vanish if the host interface is planar. To distinguish between permanent and induced quadrupoles, we define an “inert” colloid as one having no permanent multipole moment. Such colloids leave a planar interface undisturbed, yet produce quadrupolar distortions within a curved interface. Würger^19^ was the first to predict that two inert colloids (specifically, spherical colloids with equilibrium contact angles) would attract if embedded in an interface with anisotropic curvature. According to calculations by Würger^19^ and others,^20–22^ the capillary energy and lateral force of attraction between induced quadrupoles should scale *quadratically* with the deviatoric curvature, a second-order effect compared to the *linear* scaling predicted for permanent quadrupoles. Subsequent experiments published, in the same year, by Ershov *et al.*^8^ and Blanc *et al.*^21^ seem to support Würger's theory.

More recently, Sharifi-Mood *et al.*^23,24^ published experiments and theoretical calculations on the motion of cylindrical and spherical colloids in an externally imposed curvature gradient. They concluded, based on their theoretical results, that the force due to an induced capillary quadrupole should be much weaker, possibly even nonexistent, compared to Würger's prediction, and that only a permanent quadrupole could drive particle motion. The disparity between the findings of Würger and Sharifi-Mood *et al.* can be traced to how the principle of virtual work was applied to the region far from the particle. Sharifi-Mood *et al.*^24^ claimed that the work done to deform the interface in the far field exactly cancels the work done adjacent to the particle. This conclusion has drawn criticism, most notably in a series of comments by Galatola^25,28^ and Würger.^26^ Both of these authors asserted, albeit by slightly different rationale, that the work performed by a force acting on the particle cannot depend on the deformation in the far field. In particular, Galatola^25^ argued that no work is done far from the particle due to the divergence of the interface slope in that region, and that one must relax the assumption of small slopes in order to evaluate the work done on the particle. On the other hand, Würger^26^ avoided the problem of diverging interface slopes by placing a definite boundary at a fixed distance from the particle, wherein the small-slope approximation is valid. By this construction, Würger then argued that his original prediction^19^ is recovered so long as the wetting condition on the outer boundary is taken into account. Subsequent responses^27,28^ indicate that Galatola, Würger, and Sharifi-Mood *et al.* were unable to reach a clear consensus. To date, it is not obvious which approach is “correct,” leaving open the question of whether inert colloids will interact within a curved interface.

Compounding this issue is the practical challenge of measuring forces due to induced capillary quadrupoles. In large part, the difficulty lies in synthesizing truly inert colloids. Even a small amount of roughness on the surface of a colloid (say, 20 nm) undulates its contact line and creates a permanent distortion of the interface. Stamou *et al.*^30^ showed that such distortions drive large capillary forces that can overwhelm those induced by a background curvature. For instance, in the seminal experiments of Sharifi-Mood *et al.*,^23,24^ the measured capillary distortion energy due to an interface-trapped colloid (radius 5 μm) was of *O*(10^4^*kT*). This energy is consistent with contact-line undulations due to ∼20 nm of particle roughness, as measured using atomic force microscopy.^23,24^ By comparison, Galatola^25,28^ estimated that the induced quadrupole for the same colloid would contribute an *O*(10^3^*kT*) correction to the energy, which is smaller by an order of magnitude. Moreover, Sharifi-Mood *et al.*^23,24^ showed, by tracking particle trajectories, that the capillary energy scales linearly with the host interface curvature, further supporting their conclusion that a permanent quadrupole due to surface roughness drives particle motion. One potential way of suppressing these permanent quadrupoles would be to use molecularly thin colloids, such as the particles of graphene, molybdenum disulfide, and hexagonal boron nitride recently studied by Goggin *et al.*^11^ Planar particles do not possess undulated contact lines, yet do show evidence of interparticle interactions at interfaces.^10,11,60,61^

On the other hand, further theoretical insight might be gained if we revisit the virtual work calculation for an inert colloid. Domínguez *et al.*^29^ had previously raised concerns with applying the principle of virtual work to capillary problems in unbounded domains. They argued that the outer boundary condition does impact the virtual work calculation if the interfacial disturbance induced by a trapped particle does not decay “sufficiently fast.”^29^ Indeed, the effect of outer enclosing boundaries was heavily scrutinized in a separate, but related, debate in the literature regarding the possible origin of “monopolar-like” capillary attractions between charged colloidal particles trapped at oil–water interfaces.^62–75^ In order to avoid this outer boundary condition altogether, Domínguez *et al.*^29^ adopted a stress tensor formulation of capillary statics by analogy to the Maxwell stress tensor of electrostatics. By directly integrating the stress tensor over the particle boundary, they were able to reproduce the capillary force originally calculated, using virtual work arguments, by Würger.^19^ Later, Galatola and Fournier applied the stress tensor formalism to generalize Würger's result for arbitrary interface shapes.^21,22^ However, it is not yet clear, based solely on these calculations, how to reconcile the stress tensor and virtual work approaches, since the latter would appear to require information from the outer boundary. In fact, virtually all theoretical studies of particles in curved interfaces have, with few exceptions,^68,69^ focused on unbounded media, without explicitly considering the outer boundary condition.

By contrast, several relevant studies in the electrostatics literature have examined bounded domains.^40,76^ Liu *et al.*^40^ considered a dielectric particle (in 2D and 3D) bounded externally by a large shell with a prescribed potential distribution. They showed that the stress tensor method and the virtual work method yield the same expression for the dielectrophoretic force, for which there is substantial literature precedent.^32–38^ We propose that the dielectrophoretic force can be used as an analogy for the capillary force on an inert colloid, by exploiting the widely-acknowledged^2,29–31^ mathematical similarities between capillary statics and 2D electrostatics.

**Fig. 3:**
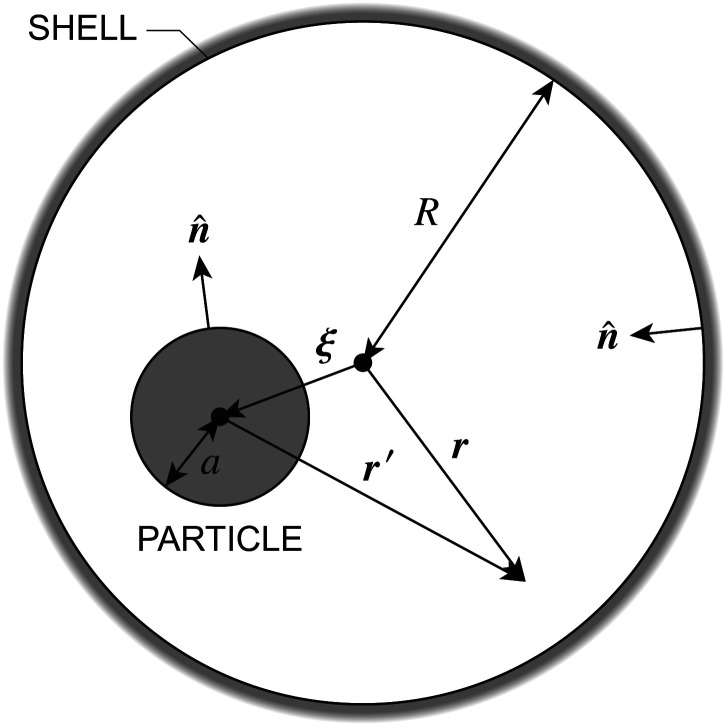
Schematic of a circular particle of radius *a* placed inside a circular shell of radius *R*, where ***ξ*** = ***r*** − ***r***′ is the particle position relative to the shell center. Note that the unit normal ***n̂*** always points into the domain, such that ***n̂*** = −***r***/*r* and ***r***′/r′ at *r* = *R* and *r*′ = *a*, respectively. The geometry applies to both the electric and capillary problems.

In this paper, we revisit the calculation of capillary forces on induced quadrupoles by way of analogy to dielectrophoretic forces on induced dipoles. We argue that the same subtleties appear in both the capillary and electric problems with regards to applying the principle of virtual work in an unbounded domain. To avoid such subtleties, we consider a bounded domain comprising a circular particle of radius *a* enclosed by a circular shell of radius *R* (Fig. 3). Sources placed on the external boundary set up a gradient in either an electric field or an interface curvature. The gradient interacts with the induced multipole moment on the particle (dipole in the electric problem, quadrupole in the capillary problem). No torque is exerted (*T* = 0), but the force ***F*** acting on the particle can be determined in one of two ways. In the first approach, the stress tensor ***σ*** is evaluated and integrated over the particle boundary,2.1
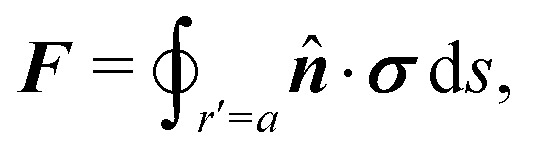
where ***n̂*** is the (outward pointing) unit normal and *r*′ is the distance measured from the particle's center. In the second approach, we first compute the energy *W* required to place the particle in the domain. Then, according to the principle of virtual work, an incremental displacement δ***ξ*** of the particle position is accompanied by a change δ*W* in the total energy:

2.2 δ*W* = −***F***·δ***ξ*** + δ*W*^ext^.

The basic laws of mechanics dictate that the force ***F*** appearing in eqn (2.2) must be given by (2.1). The last term δ*W*^ext^ in eqn (2.2) accounts for any external energy supplied to the system that is not associated with the change in the potential energy of the particle. This term is included to allow for the possibility that a virtual displacement δ***ξ*** of the particle can result in a shift of other parts of the system in order to maintain the boundary conditions. In such a case, some of the work is performed by the forces acting on those parts, in addition to the force ***F*** acting on the particle.

We show that if the boundary condition on the outer shell is neglected, then the force predicted by the virtual work method, eqn (2.2), identically vanishes in both the capillary and electric problems, as predicted by Sharifi-Mood and coworkers.^2,23,24,27^ However, this result disagrees with the force obtained by the stress tensor method, eqn (2.1). Accounting for the outer boundary condition, as originally suggested by Würger,^26^ rectifies the discrepancy between the two methods and gives a non-vanishing force. In the electric problem, we recover the well-established^32–36^ scaling of the dielectrophoretic force with the gradient of the squared electric field strength. In the capillary problem, we find that the capillary force scales with the gradient of the squared deviatoric curvature, in agreement with the findings of Würger, Galatola and coworkers.^19–22,25,26,28^ The direction of the force (up or down the gradient) depends upon the particle wetting condition.

Our work is impactful in several respects. For one, our results suggest that particles need not have undulated contact lines in order to interact on curved interfaces. To be clear, this does *not* imply that a permanent multipole due to contact-line undulation cannot lead to particle motion. Indeed, Sharifi-Mood *et al.*^23,24^ provide strong evidence that permanent quadrupoles drive colloidal capillary migration under the conditions of their experiments. Hence, their work remains an important and robust validation of the theory originally put forth by Stamou *et al.*^30^ However, our findings also suggest that induced multipoles can act *in addition to* permanent multipoles to drive particle motion – which of these two effects is dominant depends upon the properties of the particles and the host interface. For instance, molecularly thin, planar particles (*e.g.*, sheets of graphene) could interact within a curved fluid–fluid interface *via* induced capillary multipoles, whereas their permanent multipole moments would be, presumably, too weak to generate an appreciable force of attraction.

Moreover, our study is the first, to the best of our knowledge, to rigorously consider the capillary force on a particle in a bounded domain. We apply the method of reflections to approximate the particle–shell interaction, and validate this approximation using an exact solution in bipolar coordinates. Thus, we are able to show explicitly that the wetting condition on the outer boundary is a necessary ingredient in the principle of virtual work, as previously suggested by Domínguez *et al.*^29^ Handling this boundary condition can become a subtle task, with a close parallel to the electrical condition applied at an external electrode. However, the physical analogy between electrostatics and capillary statics is not complete, despite their shared mathematical structure. As we shall see, there are important distinctions between electrical and capillary work that emerge when boundaries are taken into account.

The remainder of this article focuses on paradigmatic problems in electrostatics and capillary statics for the 2D geometry sketched in Fig. 3. We begin in Section 3 with the electric problem, specifically focusing on dielectrophoretic forces exerted on ideally conducting and insulating particles. In Section 4, we consider the analogous capillary problem. There, we examine the capillary forces on colloids with either symmetrically pinned (non-undulated) contact lines or equilibrium contact angles. In the latter case, both cylindrical and spherical particles are considered. Finally, we discuss our results and provide concluding remarks in Section 5.

## 3 Electric problem

In this section, we examine dielectrophoresis of polarizable particles in an electric field as an analogy to the migration of inert colloids in a curved interface. Fig. 3 depicts a cylindrical particle (radius *a*) embedded in a dielectric medium (permittivity *ε*) and bounded externally by a cylindrical shell (radius *R*). No free charges are contained within the shell's interior. The same geometry was considered by Liu *et al.*^40^ in their study of dielectrophoresis of dielectric particles.

In the particle's absence, the electrostatic condition on the shell boundary establishes a potential *ψ*^ext^(***r***) at a position ***r*** relative to the shell's center. For simplicity, we consider the quadratic potential3.1
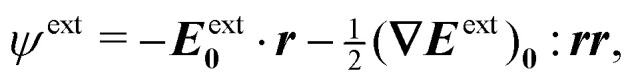
with the associated electric field

3.2 ***E***^ext^ = −**∇***ψ*^ext^ = ***E***^ext^_**0**_ + (**∇*E***^ext^)_**0**_·***r***.

Here, ***E***^ext^_**0**_ and (**∇*E***^ext^)_**0**_ are, respectively, the field and field gradient evaluated at ***r*** = **0**. It is assumed that these quantities can be controlled independently by tuning the electrostatic condition on the outer shell, as discussed below.

By the usual properties of electrostatics, the potential *ψ*^ext^ must satisfy Laplace's equation in the shell's interior,

3.3 ∇^2^*ψ*^ext^ = 0 *r* ≤ *R*,

which implies that (∇***E***^ext^)_**0**_ is both symmetric and traceless. Either of two conditions may be applied at the outer shell in order to establish the external potential given by eqn (3.1). One approach is to fix charges along the shell boundary so as to constrain the normal derivative of the potential:

3.4a ***n̂***·**∇***ψ*^ext^ = −***n̂***·***E***^ext^_**0**_ − ***n̂***·(**∇*E***^ext^)_**0**_·***r*** at *r* = *R*.

Here, the reference potential is chosen such that the average potential of the outer shell vanishes: 
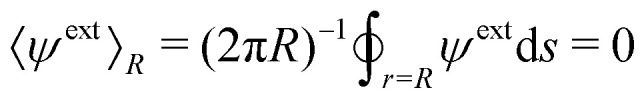
. Alternatively, one could prescribe the potential distribution at *r* = *R* by connecting the shell to a system of batteries:3.4b
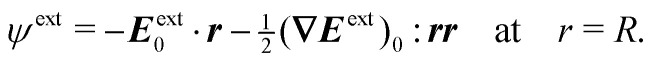
Either condition (3.4a) or (3.4b) yields (3.1) as the unique solution of (3.3). Thus, the external potential *ψ*^ext^ is insensitive to the condition applied at *r* = *R* when the internal medium is a homogeneous, linear dielectric.

The situation changes, however, when a polarizable particle is placed at ***r*** = ***ξ***. (Fig. 3 defines ***r***′ = ***r*** − ***ξ*** as the position relative to the particle's center.) Inserting the particle changes the potential to *ψ*(***r***;***ξ***), which now depends upon the electrostatic condition applied at the outer shell. To illustrate this dependence, we consider the change in electrical energy *W* required to embed the particle in the dielectric medium. Assuming the electrical properties of the particle are immaterial (*e.g.*, ideally conducting or insulating particles), then the energy *W* takes the form,3.5
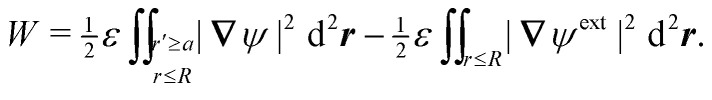
Clearly, *W* depends *non-locally* on the electric fields −**∇***ψ* and −**∇***ψ*^ext^. Information from the inner boundary *r*′ = *a* and outer boundary *r* = *R* is needed to fully specify the energy *W*. In subsequent calculations, it will be shown that the choice of electrostatic condition at *r* = *R* affects the energy even in the limit as the shell becomes infinitely large (*R* → ∞).

By contrast, the force ***F*** can only depend upon the potential disturbance in the immediate vicinity of the particle, regardless of the condition used to establish the field. This can be seen by substituting the Maxwell stress tensor3.6
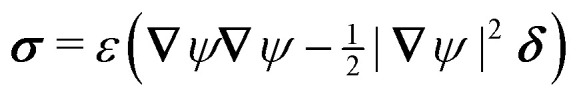
into eqn (2.1), giving3.7
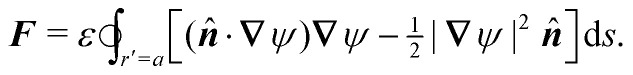
This expression shows that the force ***F*** depends only upon the *local* behavior of −**∇***ψ* near *r*′ = *a*. Of course, the force is related to the energy *W* by the principle of virtual work, eqn (2.2).

The electric potential *ψ* appearing in eqn (3.5)–(3.7) satisfies Laplace's equation,

3.8 ∇^2^*ψ* = 0 for *r*′ ≥ *a* and *r* ≤ *R*,

subject to appropriate boundary conditions at *r* = *R* and *r*′ = *a*. Again, one of two conditions may be applied at the outer shell *r* = *R*. If the charge density is fixed along the shell boundary, then3.9a
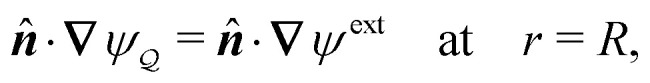
where *ψ*^ext^ is given by eqn (3.1) and the subscript 
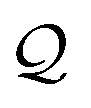
 indicates that the charge distribution is unchanged after inserting the particle. Alternatively, if the potentials are fixed by connecting the shell to a system of batteries, then3.9b
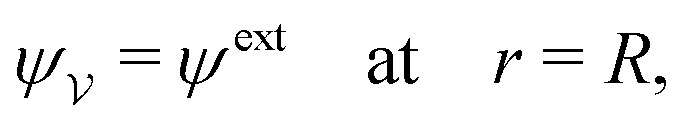
where the subscript 
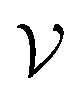
 now indicates a fixed distribution of potentials. One may specify either eqn (3.9a) or (3.9b) at the shell boundary, but not both.^77^ In either case, it is implied that the reference potential is set to the average potential of the shell.

All that remains is to specify the condition on the particle boundary *r*′ = *a*. Below, we consider two limiting cases, one where the particle acts like a perfect conductor (Section 3.1) and the other where it acts like a perfect insulator (Section 3.2). For each type of particle, the methodology for calculating the potential *ψ*, force ***F***, and energy *W* is essentially the same. Thus, to avoid redundancy, we focus the majority of our analysis on conducting particles and only briefly present the key results for insulating particles.

### 3.1 Conducting particle

We use the term “conductor” as a shorthand for particles with large dielectric constants. Traditionally, a conductor refers to a material that conducts electric current, which (for linear materials) is related to the electric field by Ohm's law. However, dielectric materials with permittivities much larger than *ε* tend to bend field lines into an orientation that is normal to their boundaries, as though they were drawing current from their surroundings.^78–80^ Thus, strong dielectrics behave like ideal conductors insofar as propagating fields, even though they cannot actually conduct currents.

At the boundary of a “conducting” particle, the tangential component of the field vanishes and an equipotential is formed:

3.10 *ψ* = *V* at *r*′ = *a*

The constant potential 
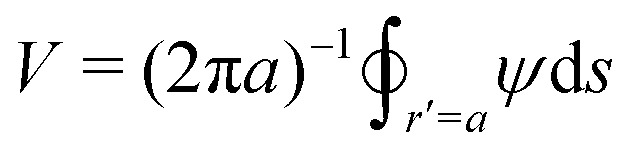
 is solely a function of the particle position ***ξ*** and is determined from the constraint that the particle carry zero net charge,3.11
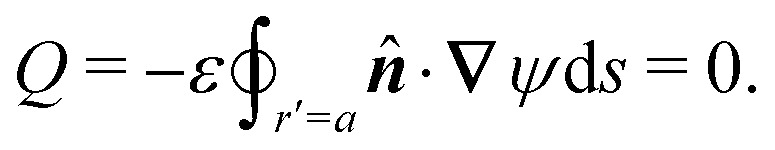
Since the charge vanishes, the first non-vanishing multipole moment on the particle is the dipole moment:3.12
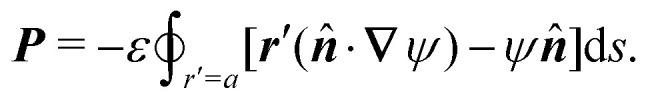


Eqn (3.8)–(3.11) comprise a linear boundary-value problem for *ψ*. For the special case where the particle is concentrically positioned inside the shell ***ξ*** = 0, the solution is straightforwardly obtained as a finite series of the 2D vector harmonics. For the more general case ***ξ*** ≠ 0, a solution is possible using the method of reflections^81–84^ (see the ESI,† Section S.1) or by eigenfunction expansions in bipolar coordinates^40,83^ (Section S.2, ESI†). Below, we present the first few reflections for the electric potential, which are sufficient to calculate the electric force and energy in the unbounded limit *R* → ∞.

**Fig. 4:**
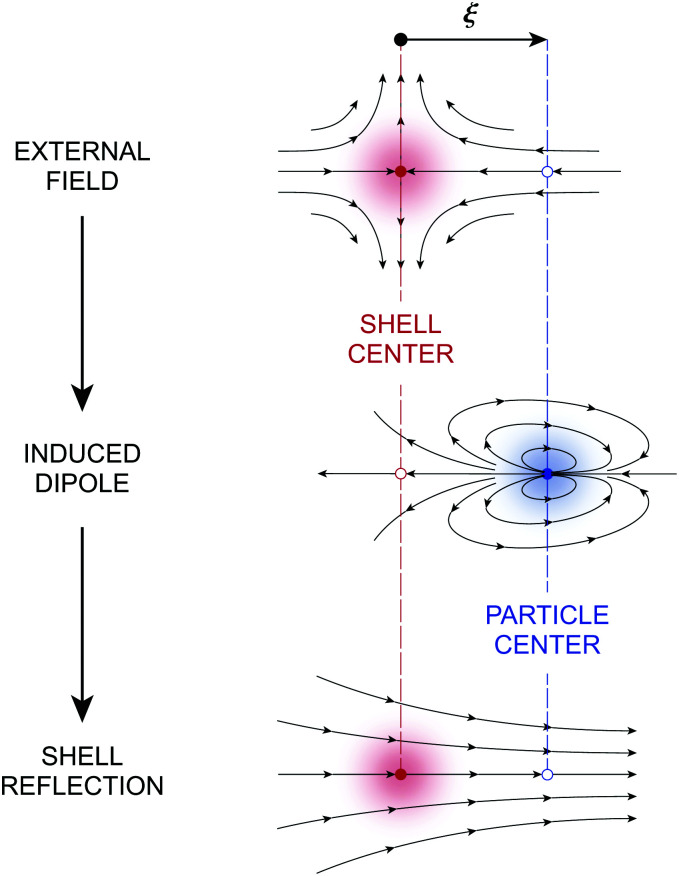
Pictorial representation of the first few reflections in the electric field 
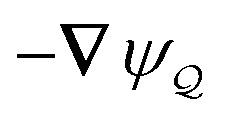
 for a conducting particle held in a system of fixed charges. For the field 
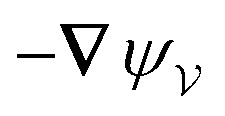
 in a system of fixed potentials, the direction of the shell reflection is reversed. In the sketch, “external field” refers to *ψ*^ext^, “induced dipole” refers to the terms ∝ ***r***′/***r***′^2^, and “shell reflection” refers to the terms ∝ ***r***/*R*^2^ in eqn (3.13) and (3.14).

Assuming *ξ* = *o*(*R*), the solution satisfying the fixed-charge condition (3.9a) is approximately given by3.13
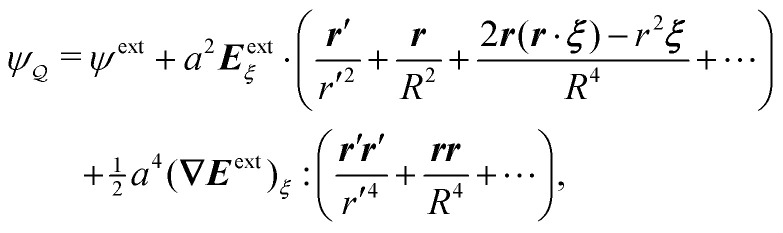
where the subscript “***ξ***” indicates evaluation at ***r*** = ***ξ***. Eqn (3.13) is derived explicitly in Section S.1.1 of the ESI.† The field lines associated with sequential reflections in eqn (3.13) are sketched in Fig. 4. Reflected modes from the particle are decaying harmonics with respect to ***r***′, with the leading contribution being the induced dipole. The shell reflections are growing harmonics with respect to ***r*** that decay as *R* → ∞; these are retained here for the virtual work calculation presented later in Section 3.1.2. Higher reflections that are omitted in eqn (3.13) contribute *O*(*a*^2^/*R*^2^) corrections in the vicinity of the particle.

If instead the potentials are fixed on the outer shell, then the proper boundary condition is given by eqn (3.9b). In this case, the approximation for the potential is3.14
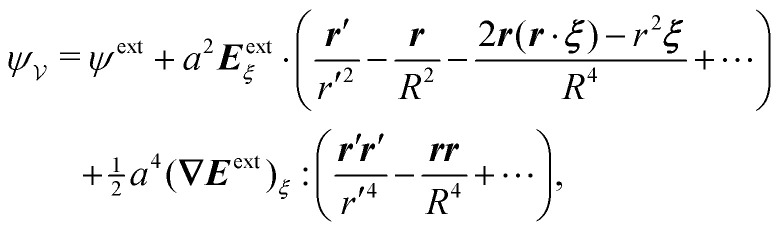
which differs from eqn (3.13) only in the sign of the shell reflections. It can be generally verified that the Neumann (fixed-charge) and Dirichlet (fixed-potential) boundary conditions on the outer shell induce harmonic reflections of opposite sign in 2D [see Appendix S.A, eqn (S.A.5)–(S.A.6), of the ESI†]. Later, we shall see that this sign reversal directly impacts the electrical energy required to insert the particle into the field.

With the solution for the potential in hand, the force can be calculated either directly, by use of the stress tensor method [*cf.*eqn (3.7)], or indirectly, by applying the principle of virtual work [*i.e.*, differentiating eqn (3.5) with respect to the particle position]. We illustrate both methods next.

#### 3.1.1 Electric force based on the stress tensor

After substituting either 
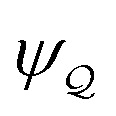
 or 
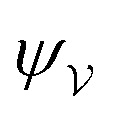
 [eqn (3.13) or (3.14)] into the stress-tensor integral (3.7) and taking the limit as *R* → ∞, we obtain

3.15 ***F*** = π*εa*^2^(**∇**|***E***^ext^|^2^)_***ξ***_,

where the higher-order terms are smaller by a factor of *a*^2^/*R*^2^. Eqn (3.15) is the canonical expression for the dielectrophoretic force on an ideally conducting, cylindrical particle.^32–36^ According to this expression, the force is directed up gradients in the external field strength |***E***^ext^|. Thus, freely floating conductors migrate to regions of high field strength – so-called *positive* dielectrophoresis.^85^ Physically, the force arises from the coupling between the background field gradient and the induced dipole moment [*cf.*eqn (3.12)]

3.16 ***P*** = 2π*εa*^2^***E***^ext^_***ξ***_,

so that eqn (3.15) has the expected form ***F*** = (***P***·**∇*E***^ext^)_***ξ***_ (*e.g.*, see Griffiths,^80^ p. 165).

It is worth reemphasizing the force on the particle is insensitive to the electrostatic condition at *r* = *R* when the medium is unbounded. This is because the Maxwell stresses depend only upon the potential disturbance in the immediate vicinity of the particle. Thus, the shell reflections in eqn (3.13) and (3.14) need not be considered. As we show below, this cannot be done when applying the principle of virtual work, because the electrical energy depends upon the potential disturbance everywhere in the domain.

#### 3.1.2 Electric force based on virtual work

In the previous section, we showed that the potentials 
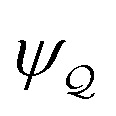
 and 
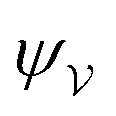
 give identical results for the force ***F*** in the limit as the shell radius *R* → ∞. The same is not true for the energy *W* because eqn (3.5) is a non-local integral equation. Special consideration must, therefore, be given to the limiting procedure when evaluating *W* in an unbounded medium. If the limit as *R* → ∞ is taken *before* evaluating the integrals in eqn (3.5), then one would incorrectly conclude that *W* = 0. We derive this result explicitly in the ESI† (Section S.1.1.2) by expanding the electric potential *ψ* up to the first particle reflection. On the other hand, taking the limit *after* integration recovers a finite energy *W* ≠ 0, as we show below.

Directly calculating the energy *W* by means of eqn (3.5) is straightforward, but tedious. For the integrals in (3.5) to converge properly, one must retain the shell reflections in the solution for the electric potential *ψ*; these are the terms that depend on *R* in eqn (3.13) and (3.14). The shell reflections decay algebraically as *R* → ∞, but are non-negligible when integrated over an *O*(*R*^2^) region. Indeed, the nonlinear coupling between the shell reflections and external field is what gives rise to the change in electrical energy. We evaluate this contribution explicitly in Section S.1.1.3 [specifically, eqn (S1.31)] of the ESI.†

A more judicious approach to calculating the energy circumvents the need to evaluate the shell reflections explicitly. Instead, by rearranging eqn (3.5), integrating by parts, and invoking the electrostatic condition (3.9), the line integral at *r* = *R* can be made to vanish identically (see Appendix A for the derivation). The remainder, to be evaluated below, is an integral solely over the particle boundary, to which the shell reflections contribute an *O*(*a*^2^/*R*^2^) correction. For the potential 
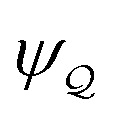
 [eqn (3.13)] satisfying the fixed-charge condition (3.9a), the insertion energy thus simplifies to3.17
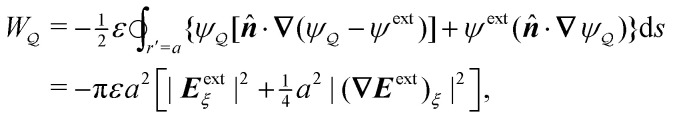
where corrections of *O*(*a*^2^/*R*^2^) vanish in the limit as *R* → ∞. On the other hand, applying the fixed-potential condition (3.9b) gives the potential 
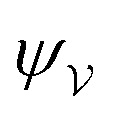
 [eqn (3.14)] and the energy3.18
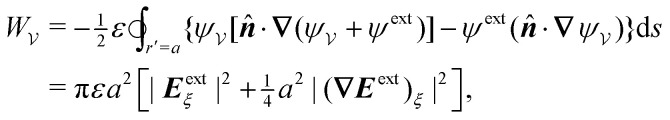
which is equal and opposite to 
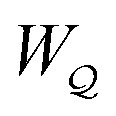
. Notably, eqn (3.17) and (3.18) predict a non-zero insertion energy because the electrostatic condition at *r* = *R* has already been applied. *Neglecting this condition altogether would lead one to conclude, incorrectly, that no energy is required to insert a conducting particle into a dielectric medium.* The difference in sign between eqn (3.17) and (3.18) can be traced to the terms containing *R* in eqn (3.13) and (3.14), even though these terms were not explicitly needed in the integration. Below, we examine how this sign reversal affects the virtual work calculation, starting with fixed-charge case.

The fixed-charge energy 
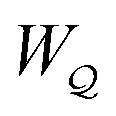
, given by eqn (3.17), depends upon the particle position ***ξ*** through the field ***E***^ext^_***ξ***_ = ***E***^ext^_**0**_ + (**∇*E***^ext^)_**0**_·***ξ***. Thus, a perturbation δ***ξ*** of the particle position is accompanied by an incremental change in energy,3.19
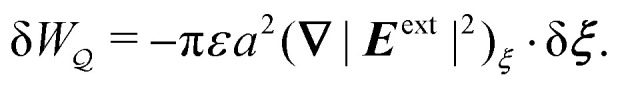
According to this expression, 
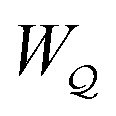
 decreases as the conducting particle moves towards the outer shell where the field is strongest. Since the source charge density is held fixed, no external work is applied to the system: 
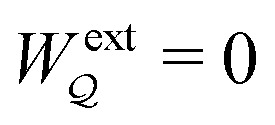
. In other words, changes in 
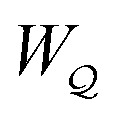
 are due solely to changes in the particle's potential energy. Thus, substituting eqn (3.19) into the virtual work principle (2.2) and rearranging gives the force3.20
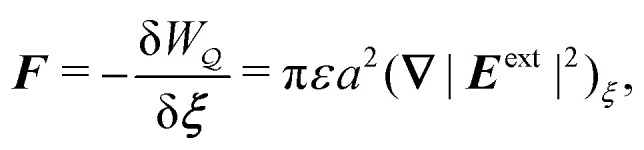
which is equivalent to the result obtained using the stress tensor method [*cf.*eqn (3.15)].

A slightly different approach is required when the potentials, rather than the charges, are held fixed on the outer shell. The fixed-potential energy 
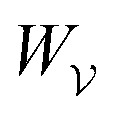
 is given by eqn (3.18), and the variation of 
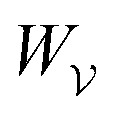
 with respect to ***ξ*** is3.21
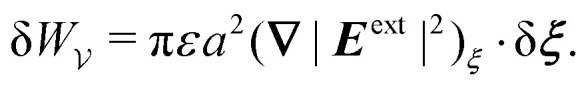
This expression would seem to imply that the force on the particle acts in the *opposite* direction, because 
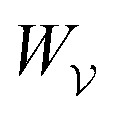
 increases as the particle is moved towards the outer shell. However, displacing the particle also alters the charge distribution on the shell in order to maintain its potential *ψ*^ext^. Since moving charges onto or off of the shell requires work, energy has to be supplied from an external power source (*e.g.*, a system of batteries). The amount of energy supplied (or work done) is3.22
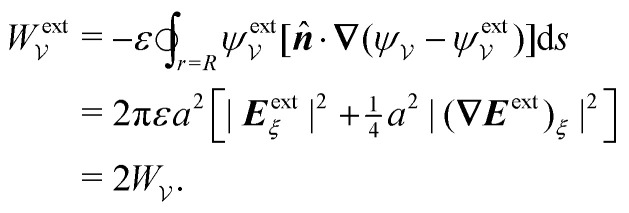
This is a classic result of electrostatics for dielectric capacitors (see, for example, Section 4.7 of Jackson^77^ or Sections 4.4.3 and 4.4.4 in Griffiths^80^). When the external field is set up by a fixed distribution of potentials, then exactly 
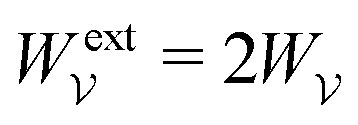
 of the energy accounts for the change in the source charge distribution. This “charging energy” must be excluded from the total electrical energy 
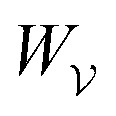
 when applying the principle of virtual work. The particle's potential energy is given by 
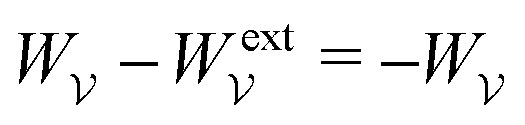
 and the force on the particle is, therefore,3.23
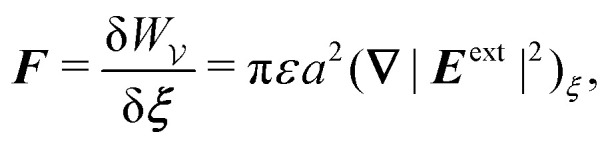
as shown previously in eqn (3.15) and (3.20).

**Fig. 5:**
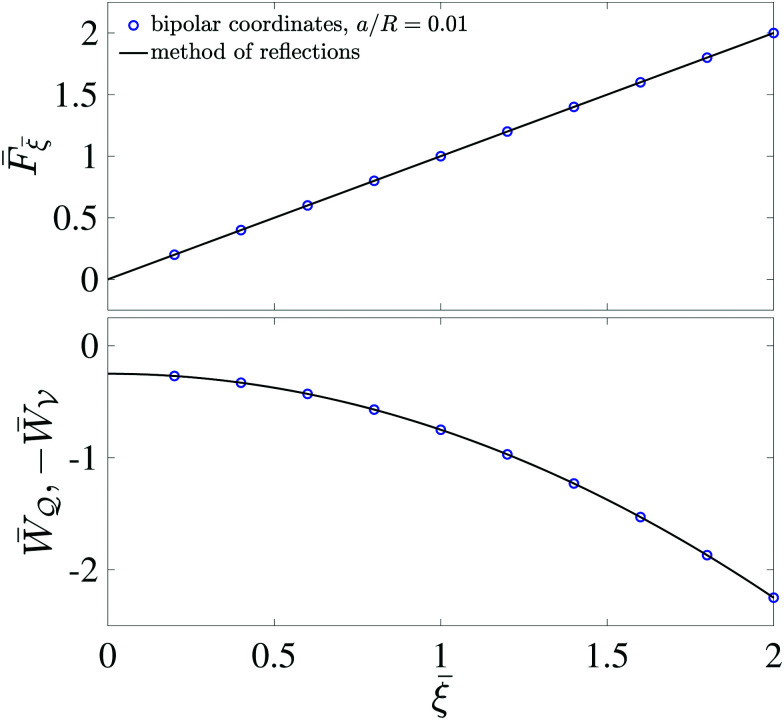
Electric force (top) and energy (bottom) plotted against the relative particle position for a conducting particle in an unbounded dielectric medium. The variables are scaled according to ***F̄*** = ***F***/[π*εa*^3^|(**∇*E***^ext^)_**0**_|^2^], *W̄* = *W*/[π*εa*^4^|(**∇*E***^ext^)_**0**_|^2^], and *ξ̄* = (***ξ*** − ξ*)/*a*, where ξ* is defined by eqn (3.24).

Irrespective of whether the charges or potentials are fixed on the outer shell, the particle's potential energy is generally given by 
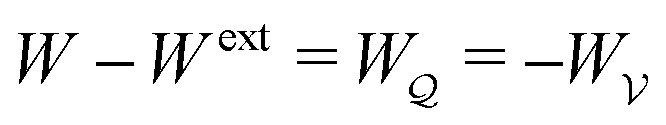
. A conducting particle's potential energy is maximized when the gradient of the local field strength vanishes. This occurs at an unstable fixed point ***ξ*** = ξ*, where ξ* is defined as3.24
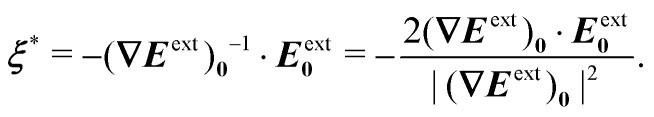
Fig. 5 plots the force ***F*** [eqn (3.15)] and energy *W* − *W*^ext^ [eqn (3.17) and (3.18)] against the relative particle position ***ξ*** − ξ*. Since the externally applied field ***E***^ext^ varies linearly with position, the force and energy are, respectively, linear and quadratic functions of ***ξ*** − ξ*. Also shown, for comparison, are the exact results for *a*/*R* = 0.01 using eigenfunction expansions in bipolar coordinates (see the ESI,† Section S.2). The two solutions show excellent agreement because the errors neglected in the reflections expansions are of *O*(*a*^2^/*R*^2^).

### 3.2 Insulating particle

The analysis presented thus far applies to conductors. We now briefly turn our attention to insulators, *i.e.*, dielectric materials with permittivities much smaller than *ε*. For insulating particles, the boundary condition (3.10) at *r*′ = *a* is replaced by

3.25 ***n̂***·**∇***ψ* = 0 at *r*′ = *a*

Thus, rather than maintaining an equipotential boundary, eqn (3.25) states that a perfect insulator expels field lines away from the boundary. The zero-charge condition (3.11) is satisfied automatically by (3.25).

Taken together, eqn (3.8), (3.9), and (3.25) comprise a boundary-value problem for an insulating particle. As in the previous section, the solution for the electric potential *ψ* can be approximated, to a satisfactory degree of accuracy, using the method of reflections (see the ESI,† Section S.1.2). The key difference to recognize is that the insulating Neumann condition (3.25) induces particle reflections of opposite sign compared to the conducting Dirichlet condition (3.10). Hence, the induced dipole moment on the insulating particle is

3.26 ***P*** = −2π*εa*^2^***E***^ext^_***ξ***_,

which is equal and opposite to eqn (3.26).

The fact that the dipole moment reverses sign implies that the energy and force are also equal and opposite to the results derived for a conducting particle [*cf.*eqn (3.17)–(3.23)]:3.27
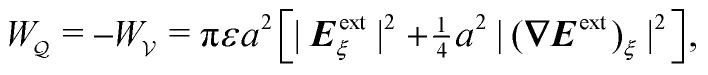
and3.28
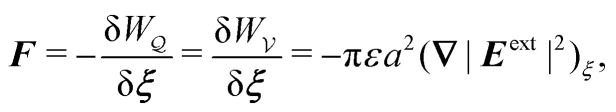
where higher-order corrections are smaller by a factor of *a*^2^/*R*^2^. Eqn (3.27) indicates that the potential energy of an insulating particle increases with field strength and is minimized at the stable fixed point ***ξ*** = ξ* [*cf.*eqn (3.24)]. As before, the difference between the fixed-charge 
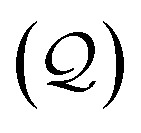
 and fixed-potential 
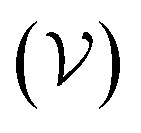
 energies can be traced to the extra “charging energy” that must be supplied in order to maintain a constant potential distribution on the outer shell [*cf.*eqn (3.22)]. However, the force ***F***, given by eqn (3.28), is independent of whether the charges or the potentials are prescribed on the outer shell. Indeed, eqn (3.28) is the canonical expression for the dielectrophoretic force on a cylindrical, insulating particle in an unbounded medium. Since the particle's potential energy increases with the local field strength, this force is directed down the gradient – so-called *negative* dielectrophoresis.

**Fig. 6:**
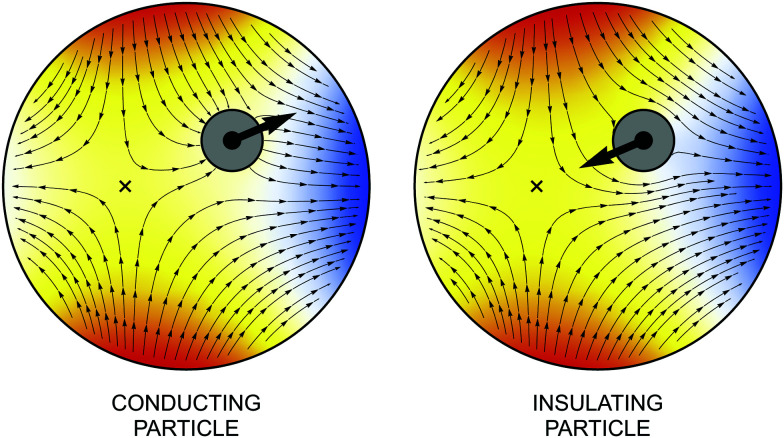
Electric potential and field lines for conducting (left) or insulating (right) particles. Field lines are directed away from regions of positive charge (*ψ* > 0, “hot” colors) towards regions of negative charge (*ψ* < 0, “cold” colors). The thick arrow indicates the direction of the electric force. The fixed point, indicated by a cross, is stable for the conductor and unstable for the insulator [*cf.*eqn (3.24)].

Fig. 6 illustrates the potential and field lines for conducting and insulating particles. For ***E***^ext^_**0**_ ≠ **0**, the fixed point ***r*** = ξ* is displaced from the shell's center ***r*** = **0**. The conducting particle attracts field lines and is drawn towards the outer shell where the field is strongest. By contrast, the insulator expels field lines and is drawn towards the fixed point.

The results presented above for cylindrical conductors and insulators can be viewed as two limiting cases of a dielectric material with permittivity *κε*, where *κ* is the dielectric constant. Dielectric particles were investigated by Liu *et al.*^40^ under conditions where the potential distribution on the outer shell was specified. The dielectrophoretic force on the particle is generally given by

3.29 ***F*** = π*εfa*^2^(**∇**|***E***^ext^|^2^)_***ξ***_,

where *f* = (*κ* − 1)/(*κ* + 1) is the 2D Clausius–Mossotti factor.^39^ In the strong-dielectric limit (*κ* → ∞), *f* = 1 and eqn (3.29) simplifies to (3.15). In the opposite limit (*κ* → 0) for weak dielectrics, *f* = − 1 and we recover eqn (3.28).

This concludes our analysis of the electric problem. Although the literature on this problem is well established, we carried out the calculations explicitly to illustrate the subtleties that emerge, though often under-appreciated, when applying the principle of virtual work. Importantly, if the electrostatic condition at *r* = *R* is neglected, then one might incorrectly conclude that no work is needed to move the particle within the field. Accounting for this condition is necessary to calculate the energy and recover the well-known result [eqn (3.29)] for the dielectrophoretic force, even in the unbounded limit *R* → ∞. Below, we show that the same issues arise in the calculation of capillary forces on particles trapped at fluid–fluid interfaces.

## 4 Capillary problem

In this section, we consider an inert, circularly symmetric particle trapped at a curved fluid–fluid interface (interfacial tension *γ*) bounded externally by a cylindrical shell (Fig. 3). The effect of a static pressure, *e.g.*, due to gravity, is neglected in our analysis. The vertical position of the interface is defined, respectively, by *z* = *ζ*^ext^(***r***) and *ζ*(***r***;***ξ***) before and after the particle is embedded in the interface. For simplicity, we assume that the host interface height *ζ*^ext^ is composed of a “saddle” plus a “monkey saddle,”4.1
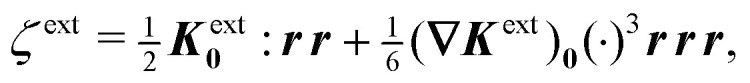
with the associated curvature tensor

4.2 ***K***^ext^ = **∇∇***ζ*^ext^ = ***K***^ext^_**0**_ + (**∇*K***^ext^)_**0**_·***r***.

Here, ***K***^ext^_**0**_ and (**∇*K***^ext^)_**0**_ denote the curvature and curvature gradient evaluated at ***r*** = **0**. Other relevant geometric properties of the interface are defined in Appendix B.

Assuming small slopes |**∇***ζ*^ext^| ≪ 1, the governing equations of capillary statics are equivalent to those of electrostatics,^16^ with the interface height taking the place of the electric potential. Hence, *ζ*^ext^ satisfies Laplace's equation,

4.3 ∇^2^*ζ*^ext^ = 0 for *r* ≤ *R*.

Eqn (4.3) implies that ***K***^ext^_**0**_ and (**∇*K***^ext^)_**0**_ are both symmetric and traceless, characteristic of an interface with vanishing mean curvature but a finite and inhomogeneous deviatoric curvature [for the definition of the deviatoric curvature, see eqn (B.10) of Appendix B]. The cubic interface profile can be established either by fixing the height (*i.e.*, the position of the contact line) at the outer boundary,4.4a
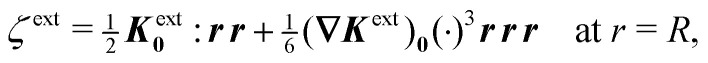
or by fixing the slope (*i.e.*, the contact angle),4.4b
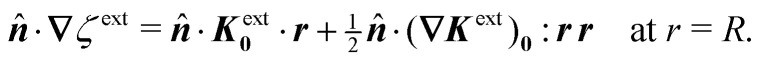


We note that eqn (4.1) is expanded up to cubic order in ***r***, whereas (3.1) is truncated after the quadratic term, due to the physical constraints imposed on the interface height as compared to the electric potential. In the electric problem, the reference potential is taken to be the average potential of the outer shell: 
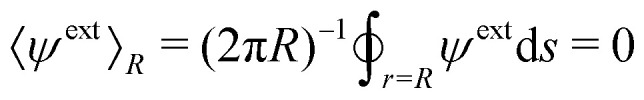
. This constraint eliminates the constant term in eqn (3.1). In the capillary problem, the analogous constraint requires the interface height to be measured in a frame that is translated and rotated into the average height and slope of the outer shell: 
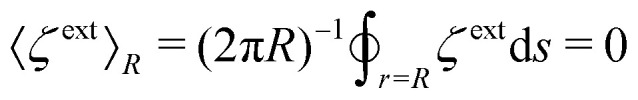
 and 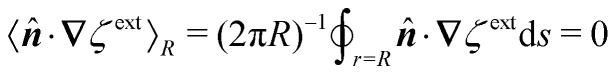. Thus, both the constant and linear terms vanish in eqn (4.1).

**Fig. 7:**
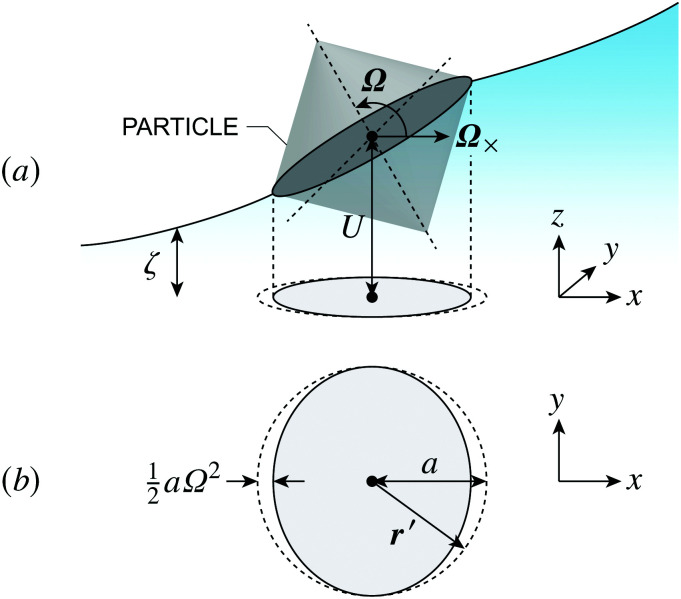
(a) 3D illustration of an interface-trapped particle with a circular contact line translated vertically by *U* and rotated horizontally by ***Ω*** with respect to the undeformed plane. The vector 
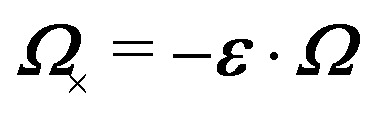
 is orthogonal to ***Ω***. (b) 2D illustration of how rotation deforms the boundary in the undeformed plane. A point ***r***′ measured from the center of the particle to a circle of radius *a* is transferred to a new point 
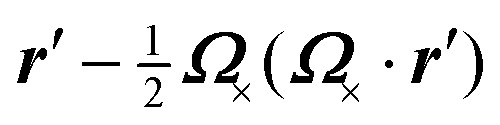
 on an ellipse.

Placing a colloidal particle at a horizontal position ***r*** = ***ξ*** distorts the interface, causing it to adopt a new profile *ζ*(***r***;***ξ***). In the small-slope limit |**∇***ζ*| ≪ 1 and |**∇***ζ*^ext^| ≪ 1, the capillary distortion energy *W* associated with inserting the particle may be expanded up to quadratic order in the interface slope (see Appendix C for the derivation). Assuming the contact line is circular with radius *a*, this energy is given as4.5
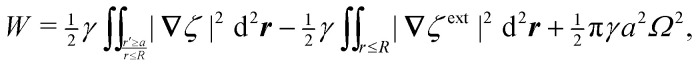
where ***Ω*** is the rotation of the particle out of the horizontal plane. The last term in eqn (4.5) accounts for the *O*(*Ω*^2^) distortion of the contact line in the horizontal plane (illustrated in Fig. 7).

Eqn (4.5) excludes the “trapping energy” −π*γa*^2^, which represents the work done to eliminate a circular patch of interfacial area. Thus, the energy *W* accounts only for distortions of the interface curvature and vanishes if *ζ* = 0 [in this sense, *W* is directly analogous to the electrical energy defined by eqn (3.5)]. If the contact line is non-circular in the rotated frame, then additional terms must be added to eqn (4.5) to account for the distortion of the particle boundary (an example is given later, in Section 4.2.2).

The capillary analogue of the Maxwell stress tensor was derived by Domínguez *et al.*^29^ and is given by4.6
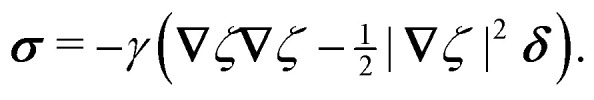
The capillary stresses result from the nonlinear curvature-capillary interaction between the particle and the surrounding interface. Substituting eqn (4.6) into (2.1) then yields the lateral force on the particle:4.7
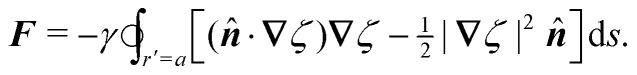
(For the derivation of the last two expressions, see Appendix D.) Domínguez *et al.*^29^ noted that eqn (4.6) and (4.7) bear resemblance to their electrical analogues (3.6) and (3.7), but for the difference in sign of the right-hand side. We propose to complete the analogy by introducing the imaginary unit 
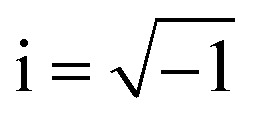
 and the substitutions *ε* → *γ* and *ψ* → i*ζ*. By defining an “imaginary potential,” the sign of Coulomb's force law is reversed. Hence, an electric charge density *q* = −*ε*∇^2^*ψ* can be made analogous to a capillary pressure (or force density) *p* = −*γ*∇^2^*ζ* by setting *q* → i*p*. The inversion of the force law implies that like “capillary charges” attract, with positive and negative charges referring, respectively, to the vertical forces that displace the interface above and below the undeformed plane.^86^

We are left to evaluate the interface height *ζ* appearing in eqn (4.5)–(4.7). This is done by solving Laplace's equation,

4.8 ∇^2^*ζ* = 0 for *r*′ ≥ *a* and *r* ≤ *R*,

subject to boundary conditions at *r*′ = *a* and *r* = *R*. Two possibilities exist for the latter condition. Either the interface heights on the outer shell are fixed,4.9a
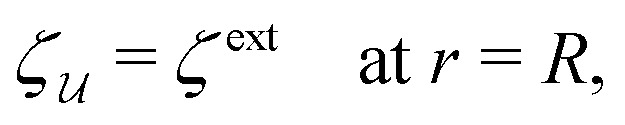
or the interface slopes are fixed,4.9b
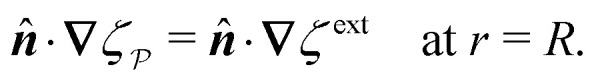
Here, the subscripts 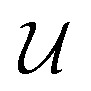 and 
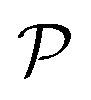
 denote, respectively, the fixed-height and fixed-slope conditions. In both cases, we assume that the reference frame is translated and rotated into the average height and slope of the outer shell.

From here, the procedure is similar to the one laid out in Section 3. First, we evaluate the interface height around a particle with a pinned contact line (Section 4.1), which is the mathematical analogue of a 2D electrical conductor. As in the electric problem, we employ the stress tensor and the principle of virtual work to compute the capillary force on the particle. The analysis is presented explicitly for particles with symmetrically pinned contact lines, and proper consideration is given to the wetting condition on the outer boundary. Our virtual work calculation reveals, somewhat surprisingly, that the interface height at a boundary plays a similar role to the electric charge density. Subsequently, we consider cylindrical and spherical particles with equilibrium contact angles (Section 4.2). We show that equilibrated cylinders are analogous to 2D electrical insulators with a boundary charge distribution, whereas spheres are akin to insulators with a boundary distribution of radially oriented dipoles.

### 4.1 Particle with a pinned contact line

**Fig. 8:**
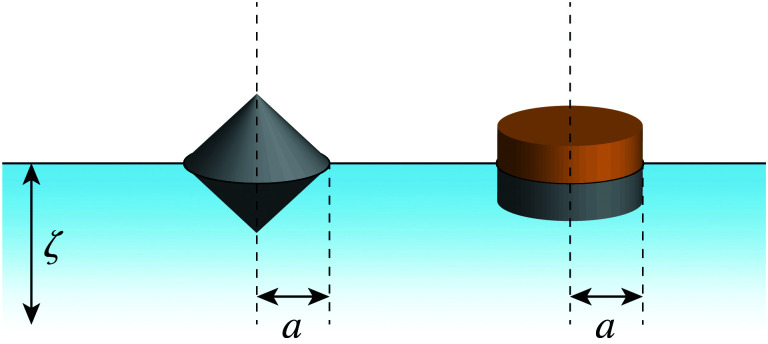
Conceptual strategies for pinning the contact line at a circular particle boundary. (left) Bicone-shaped particle with a sharp edge. (right) “Janus” particle with heterogeneous surface chemistry.

In principle, there are several ways in which to pin the contact line to the particle boundary; two possibilities are sketched in Fig. 8. One strategy is to design the shape of the particle to have a sharp edge (*e.g.*, the bicone shape depicted in Fig. 8, left). Another option is to synthesize a “Janus” particle with heterogeneous surface chemistry (Fig. 8, right). In this section, we consider an interface-trapped particle with a pinned, circularly symmetric contact line of radius *a*. The effect of a static contact-line undulation, *e.g.*, due to an irregular particle shape^14^ or surface roughness,^23,24,30^ is not our primary focus (although it is briefly considered in Appendix E). As was discussed in Section 2, such static undulations can produce a permanent capillary quadrupole (as well as higher multipoles^31,56^) in the interface shape. Below, we focus only on the *induced* quadrupole.

Assuming the particle is translated vertically by *U* and rotated horizontally by ***Ω*** (Fig. 7a), the general boundary condition for a particle with a symmetrically pinned contact line is

4.10 *ζ* = *U* + ***Ω***_×_·***r***′ at *r*′ = *a*

where 
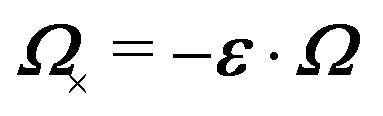
 is the vector orthogonal to ***Ω*** in the horizontal plane. The constants 
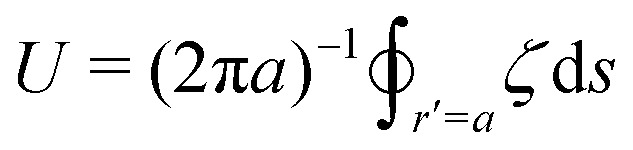
 and 
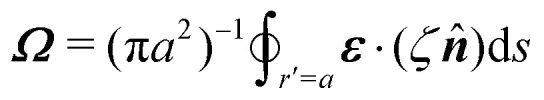
 are solely functions of ***ξ*** and must be determined by applying force and torque balances to the particle. Mechanical isolation requires that the transverse force *P* on the particle vanishes:4.11
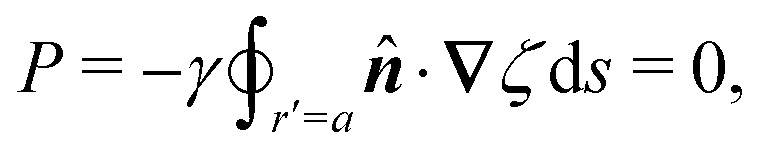
which, for isotropic particles like the ones considered here, uniquely specifies the particle translation *U*. Similarly, the rotation ***Ω*** is determined by insisting that the lateral torque ***N*** on the particle also vanishes:4.12
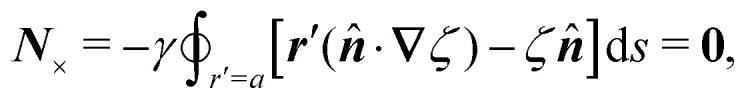
where 
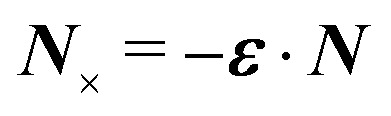
 is the vector orthogonal to ***N***. Following our aforementioned convention of an “imaginary potential,” the quantities i*P* and i***N***_×_ are, respectively, analogous to the charge *Q* and polarization ***P*** of electrostatics [*cf.*eqn (3.11) and (3.12)]. Since both the monopole and dipole moments vanish, the first non-vanishing multipole moment is the quadrupole moment:4.13
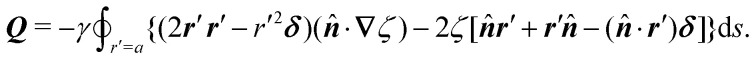


Following Section 3.1, we approximate the solution of eqn (4.8)–(4.12) for the interface height *ζ* using the method of reflections (see the ESI,† Section S.3) and verify this approximation using an exact solution in bipolar coordinates (Section S.4, ESI†). The first few reflections are presented in eqn (4.14) and (4.15), below. If the fixed-height condition (4.9a) at the outer shell is satisfied, then the solution for the interface height is given by4.14
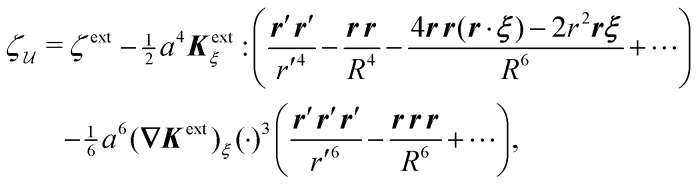
whereas the solution satisfying the fixed-slope condition (4.9b) is4.15
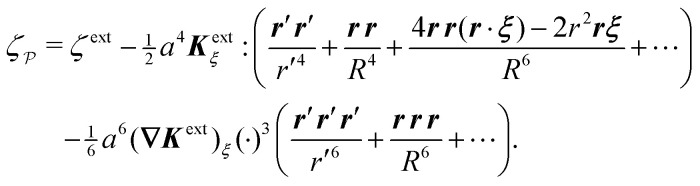
Here, it is implicitly assumed that *ξ* = *o*(*R*). For a detailed derivation of eqn (4.14) and (4.15), see Section S.3.1 of the ESI.†

**Fig. 9:**
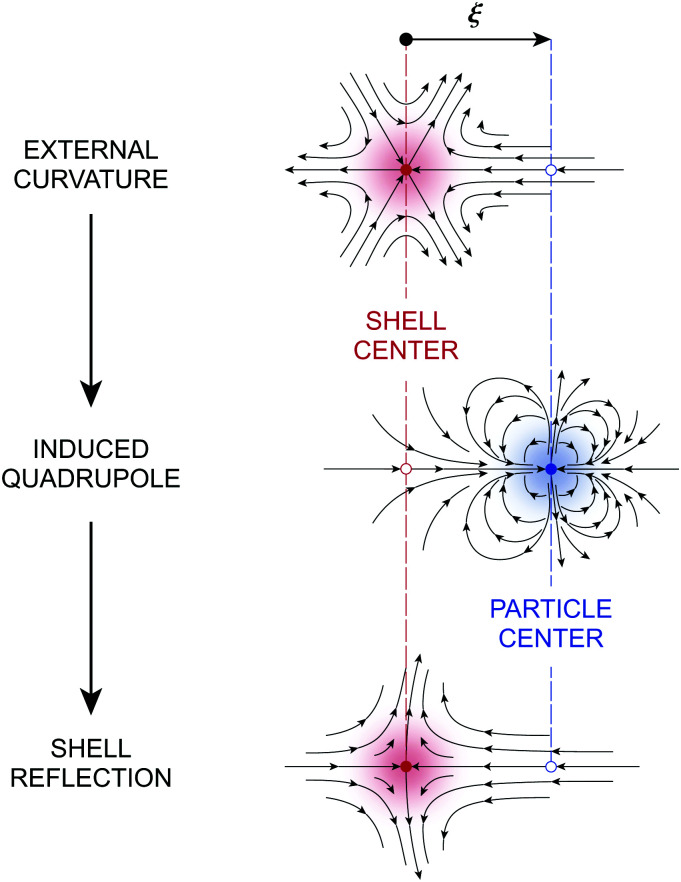
Pictorial representation of the first few reflections in the interface gradient 
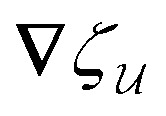
 for a particle with a pinned contact line trapped at an interface with a fixed outer height distribution. For the gradient 
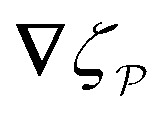
 defined by a fixed outer slope distribution, the direction of the shell reflection is reversed. In the sketch, “external curvature” refers to *ζ*^ext^, “induced quadrupole” refers to the terms ∝ ***r***′***r***′/*r*′^4^, and “shell reflection” refers to the terms ∝***rr***/*R*^4^ in eqn (4.14) and (4.15).

Fig. 9 depicts the various contributions to *ζ*. The externally imposed interface height *ζ*^ext^ (Fig. 9, top) induces a capillary quadrupole emanating from the particle (Fig. 9, middle). This disturbance propagates to the outer shell, inducing additional reflected modes that constrain either the outer height or the outer slope (Fig. 9, bottom). The two solutions (4.14) and (4.15) are similar but for the difference in sign of the shell reflections [recall that the same result emerged in the electric problem, *cf.*eqn (3.13) and (3.14)]. Higher reflections, which will not be needed in our analysis, contribute *O*(*a*^4^/*R*^4^) corrections to the interface height near the particle. Compared to the electric problem, these corrections are substantially weaker due to the faster decay of the capillary quadrupole (∼1/*r*′^2^) compared to the electric dipole (∼1/*r*′).

An important distinction between eqn (4.14) and (4.15) and their electrostatic analogues (3.13) and (3.14) is the sign of the first reflected mode emanating from the particle. For an electrical conductor, the induced electric dipole moment ***P*** is aligned with the local electric field ***E***^ext^_***ξ***_, as shown by eqn (3.16). Consequently, polarized conductors tend to migrate towards regions of high field strength. On the other hand, the induced capillary quadrupole moment ***Q*** for a particle with a pinned contact line is *anti-aligned* with the local curvature ***K***^ext^_***ξ***_, *viz.*,

4.16 ***Q*** = − 2π*γa*^4^***K***^ext^_***ξ***_

by use of eqn (4.13). As we show below, this anti-parallel alignment implies that the force on a particle with a pinned (non-undulated) contact line is directed towards regions of low deviatoric curvature. We first compute this force by directly integrating the capillary stress tensor over the particle boundary [eqn (4.7)]. Then, we apply the principle of virtual work to derive the same force [by differentiating eqn (4.5) with respect to ***ξ***].

#### 4.1.1 Capillary force based on the stress tensor

In an unbounded interface, the lateral force on the particle can be calculated by substituting either 
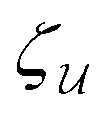
 or 
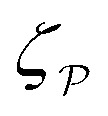
 [eqn (4.14) or (4.15)] into the stress-tensor integral (4.7) and subsequently taking the limit as *R* → ∞. Thence, we obtain4.17
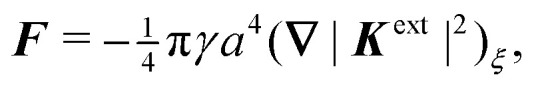
where the terms neglected are smaller by a factor of *a*^4^/*R*^4^. Using eqn (D.11) in Appendix D, we may show that the transverse torque *T* on the particle identically vanishes.

Eqn (4.17) shows that the force acts against the curvature gradient if the contact line on the particle is pinned. Using the definition of the induced quadrupole moment given by eqn (4.16), the force may be rewritten as 
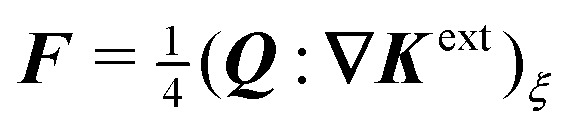
. This form is equivalent, save for a factor of two, to eqn (39) in the article by Domínguez *et al.*^29^ The analogous dielectrophoretic force on an electrically conducting particle in a dielectric medium is given by eqn (3.15).

Sharifi-Mood and coworkers^2,23,24,27^ arrived at a different conclusion based on virtual work arguments. Contrary to eqn (4.17), these authors found that the capillary force on an inert particle vanishes in a curvature gradient. In the next section, we uncover the source of discrepancy between our approach and theirs by applying the virtual work method to compute the force on the particle.

#### 4.1.2 Capillary force based on virtual work

As discussed previously in Section 3.1.2, the issue with using the energy *W* to calculate the force ***F*** is that the condition on the outer boundary must be taken into account. Indeed, this very issue was raised by Domínguez *et al.*^29^ in their comparison of the stress tensor and virtual work methods to compute capillary forces. The shell reflections in eqn (4.14) and (4.15) ensure that the interface height *ζ* satisfies the boundary condition (4.9) at *r* = *R*. If these reflections are neglected in the limit as *R* → ∞, then a direct evaluation of eqn (4.5) gives *W* = 0 as predicted by Sharifi-Mood *et al.*^23,24^ In the ESI,† we explicitly derive this result by truncating the solution for the interface height after the first particle reflection (see Section S.3.1.2, ESI†). However, this result is incompatible with the force (4.17) obtained *via* the stress tensor. In Section S3.1.3 of the ESI,† we show that including the shell reflections in eqn (4.5) gives *W* ≠ 0 and rectifies the disparity between the stress tensor and virtual work methods. This is, in fact, the very resolution offered by Würger.^26^ Below, we arrive at the same result without having to evaluate the shell reflections explicitly.

In lieu of evaluating the energy *W* by “brute force,” a much simpler approach is to factorize the integrand of eqn (4.5), integrate by parts, and use the boundary condition (4.9) to eliminate the integral over *r* = *R* (see Appendix A). This leaves only the line integral over the particle boundary to be evaluated, and the shell reflections contribute an *O*(*a*^4^/*R*^4^) correction to this integral. Using 
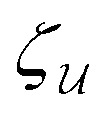
 from eqn (4.14), we may evaluate the capillary distortion energy for an interface with fixed outer heights:4.18
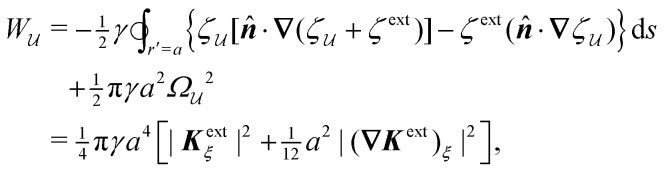
where corrections of *O*(*a*^4^/*R*^4^) vanish asymptotically as *R* → ∞. Identifying 
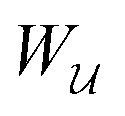
 as the particle's potential energy, we may then use the virtual work principle (2.2) to derive the force on the particle. Varying 
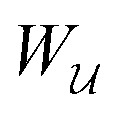
 with respect to ***ξ*** gives4.19
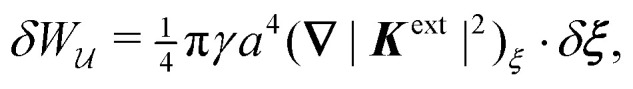
from which the force ***F*** is obtained upon rearrangement:4.20
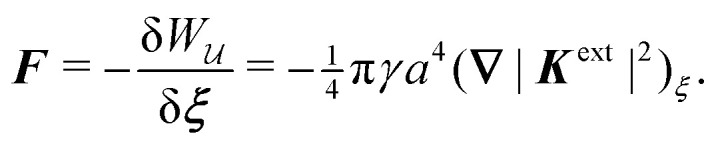
Notably, the capillary force based on virtual work [eqn (4.20)] is equivalent to the force based on the stress tensor [eqn (4.17)].

Eqn (4.18)–(4.20) may be rationalized as follows. First, by assuming the interface height 
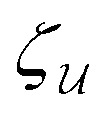
 is fixed at an outer radius *R*, the resulting distortion energy 
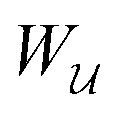
, given by eqn (4.18), is non-zero. Moreover, no external work is needed to displace the particle (
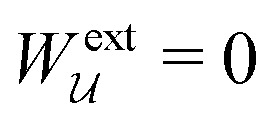
), which allowed us to relate the force ***F*** to the negative derivative of 
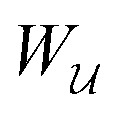
 with respect to ***ξ*** [eqn (4.20)]. The last result is interesting because the mathematically analogous problem of electrostatics, that of fixed potentials on the outer shell, requires some extra work to be done in order to move a particle through an electric field. We showed in Section 3.1.2 that this work must be supplied by an external power source in order to adjust the charge distribution on the outer shell [*cf.*eqn (3.22)]. In capillary statics, fixing the height (the mathematical analogue of the potential) does not require any extra work.

**Fig. 10:**
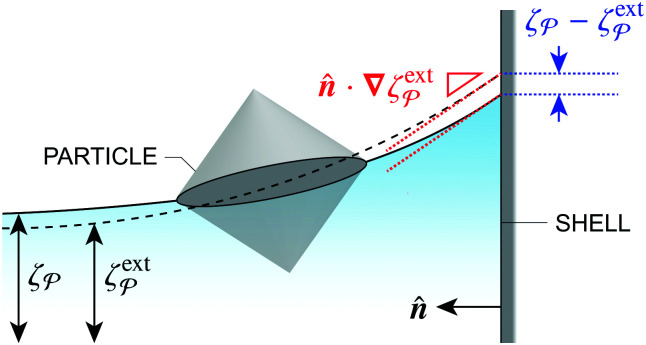
Illustration of the interface displacement 
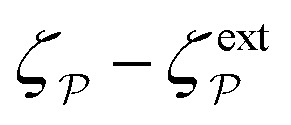
 induced by the particle while maintaining a fixed slope 
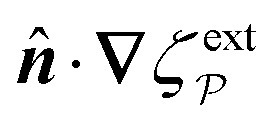
 at the shell boundary. The Young–Dupré work of adhesion, given by eqn (4.23), accounts for the change in the wetted area on the shell surface.

The opposite is true when the interface slopes, rather than the heights, are prescribed on the outer shell. Following the same procedure as above, we may calculate the distortion energy 
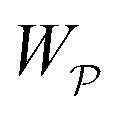
 associated with the deformation 
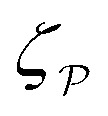
 [eqn (4.15)] for fixed outer slopes. In the limit as *R* → ∞, we obtain4.21
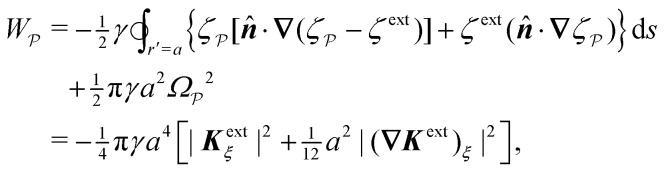
which is equal and opposite to 
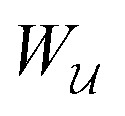
. Likewise, the variation of 
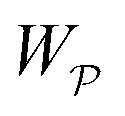
 with respect to ***ξ*** is equal and opposite to eqn (4.19),4.22
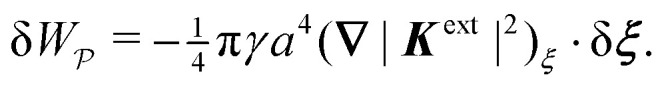
This expression shows that the capillary energy, somewhat non-intuitively, *decreases* as the particle moves towards regions of high curvature. The reduction in energy is attributed to the adjustment of the interface height at the shell boundary in order to maintain a fixed slope (see Fig. 10 for an illustration). As the outer height changes, so does the wetted area on the shell surface. The energy required to alter the wetted area is given by the Young-Dupré work of adhesion,4.23
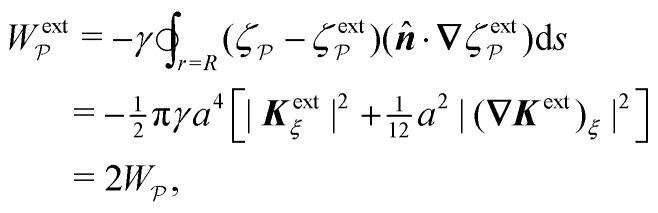
which is physically analogous to the “charging energy” defined by eqn (3.22). In this sense, the interface height *ζ* at a fluid–solid boundary plays a similar role to the electric charge density −*ε*(***n̂***·**∇***ψ*). Since the wetted area on the boundary is not fixed, one must account for the change in the adhesion energy 
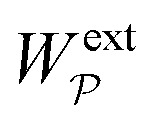
 when applying the principle of virtual work. Thence, we identify 
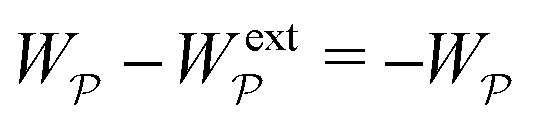
 as the potential energy of the particle in a curved interface with externally fixed slopes. Using eqn (2.2) and (4.22), the force on the particle is then given by4.24
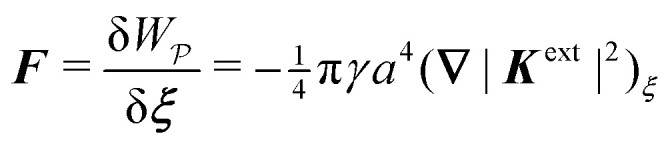
as before.

The particle's potential energy 
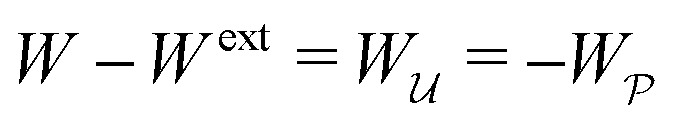
 is minimized at a stable fixed point ***ξ*** = ξ*, where the gradient of deviatoric curvature vanishes:4.25
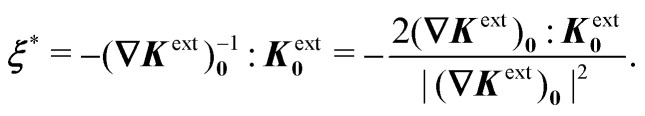
Physically, this energy minimum can be understood as the region of the interface that best accommodates the shape of the contact line. In the analogous electrical problem, the point ***ξ*** = ξ* [defined by eqn (3.24)] is unstable (energy-maximizing) for a conducting particle. That is, electrical conductors are attracted to regions of high field strength, whereas particles with pinned contact lines are attracted to low-curvature regions of the interface.

**Fig. 11:**
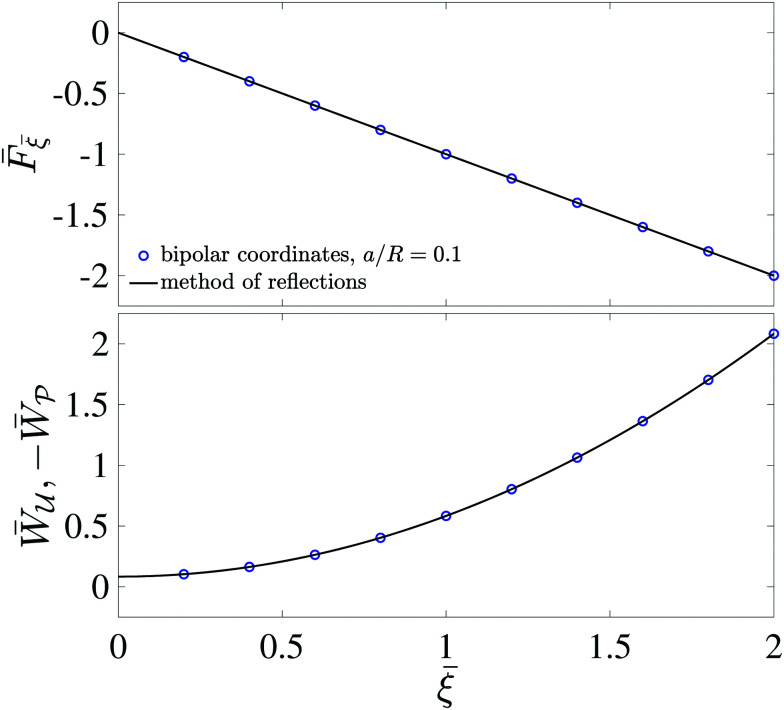
Capillary force (top) and energy (bottom) plotted against the relative particle position for a particle with a circularly pinned contact line trapped at an unbounded interface. The variables are scaled according to 
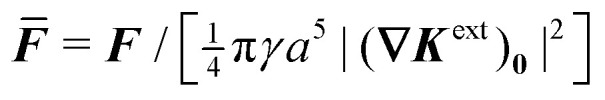
, 
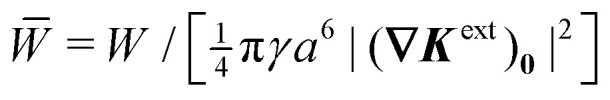
, and ***ξ̄*** = (***ξ*** − ξ*)/*a*, where ξ* is defined by eqn (4.25).

Fig. 11 plots the force ***F*** [eqn (4.17)] and energy *W* − *W*^ext^ [eqn (4.18) and (4.21)] against the relative particle position ***ξ*** − ξ*. Also plotted are results for *a*/*R* = 0.1 obtained by means of eigenfunction expansions in bipolar coordinates (see the ESI,† Section S.4). The two solutions are indistinguishable because the *O*(*a*^4^/*R*^4^) errors in the reflections solution are negligibly small. As *a*/*R* is increased, higher-order corrections contribute more significantly to the force on the particle; these corrections depend upon whether the heights (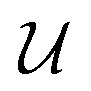) or slopes (
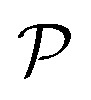
) are fixed on the outer shell. Further details on finite-size effects can be found in Section S.3.1.5 of the ESI.†

The preceding results explicitly assume that the interface is pinned to a circularly symmetric particle. In the presence of a curvature gradient (*e.g.*, a “monkey saddle” surface), such particles experience a capillary force against the gradient that drives them towards regions of low curvature. If the contact line is asymmetrically pinned (*i.e.*, undulated), then the coupling between the asymmetric distortion of the interface to the curvature gradient drives the particle towards regions of *high* curvature.^23,24,30^ This effect is neglected in our analysis above, but is briefly considered in Appendix E.

### 4.2 Particle with an equilibrium contact angle

Our final calculation focuses on interface-trapped particles with equilibrium contact angles instead of pinned contact lines. Spherical colloids with equilibrium wetting properties were first analyzed by Würger^19^ and later re-examined by numerous investigators.^20–28^ It is, therefore, appropriate that our analysis has come full circle to this original problem. We shall see that a particle with an equilibrium contact angle is the capillary analogue of an electrical insulator.

**Fig. 12:**
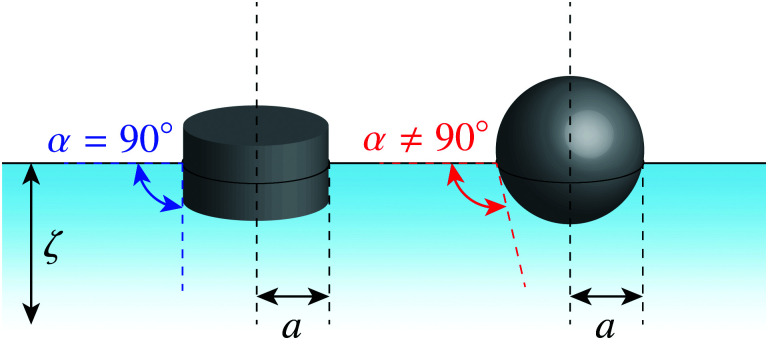
Particles with an equilibrium contact angle *α*. (left) Cylindrical particle with *α* = 90°. (right) Spherical particle with *α* ≠ 90°.

Since the contact line is free to move, the pinned-contact-line condition (4.10) no longer applies. The proper wetting condition for a fixed contact angle depends upon the particle shape. Fig. 12 illustrates the equilibrium wetting condition for cylindrical and spherical particles. A cylindrical particle (Fig. 12, left) is free to translate in the transverse direction but must rotate to an orientation perpendicular to the interface plane in order balance lateral torques. If the particle's buoyant weight is negligible, then a balance of transverse forces restricts the contact angle to 90°; otherwise, the interface would slip until it pins to the edge of the cylinder. By comparison, a spherical particle (Fig. 12, right) is rotationally invariant yet translates until the interface meets the boundary at a prescribed contact angle; this ensures that transverse forces are balanced. Since the particle translation is an extra degree of freedom, the contact angle is unrestricted for spheres.

In the presence of a background curvature, the interfacial distortions induced by cylindrical and spherical particles are distinct and must be treated separately. Below, we first consider the wetting condition for a cylindrical particle with a contact angle of 90° (Section 4.2.1). Then, we examine a spherical particle without placing any restrictions on the contact angle (Section 4.2.2). In both cases, we denote by *a* the radius of the circular contact line in a planar interface. We shall see that the capillary force is qualitatively similar for both cylindrical and spherical particles, but for a difference in the numerical prefactor.

#### 4.2.1 Cylindrical particle

For a cylindrical particle with a contact angle of 90°, the equilibrium wetting condition constrains the slope of the interface at the particle boundary:

4.26 ***n̂***·**∇***ζ* = ***n̂***·***Ω***_×_ at *r*′ = *a*,

where the particle rotation 
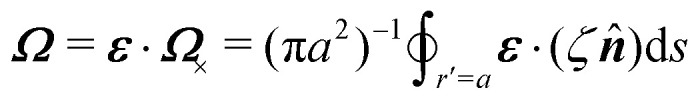
 is determined from the torque-free condition (4.12). The force-free condition (4.11) is satisfied automatically by (4.26); thus, the particle translation does not enter into the problem. This reflects the fact that a cylinder is translationally invariant along its axis. It should be noticed that eqn (4.26) is mathematically analogous to the insulating Neumann condition (3.25), except that the right-hand side is non-zero.

Solving eqn (4.26), (4.9), (4.12), and (4.26) for *ζ* is straightforward using the method of reflections (see the ESI,† Section S.3.2.1). The solution is similar to the one found in Section 4.1 for a particle with a pinned contact line [*cf.*eqn (4.14) and (4.15)], except that the particle reflections reverse sign. Consequently, the induced quadrupole moment is equal and opposite to eqn (4.16):

4.27 ***Q*** = 2π*γa*^4^***K***^ext^_***ξ***_.

Applying the same methods as in Section 4.1, the potential energy and force for a cylindrical particle with an equilibrium contact angle of 90° are, respectively,4.28
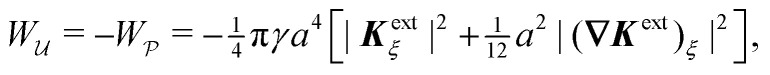
and4.29
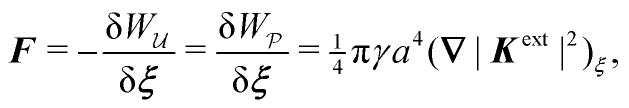
where corrections of *O*(*a*^4^/*R*^4^) have been neglected. Comparison with eqn (4.18)–(4.24) reveals that the energy and force have reversed sign. That is, the equilibrium wetting condition drives particles towards more curved regions of the interface. The difference between 
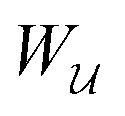
 and 
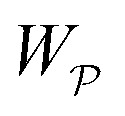
 in eqn (4.28) is attributed to the Young–Dupré work of adhesion needed to maintain a fixed slope distribution on the outer shell [*cf.* eqn (4.23)].

**Fig. 13:**
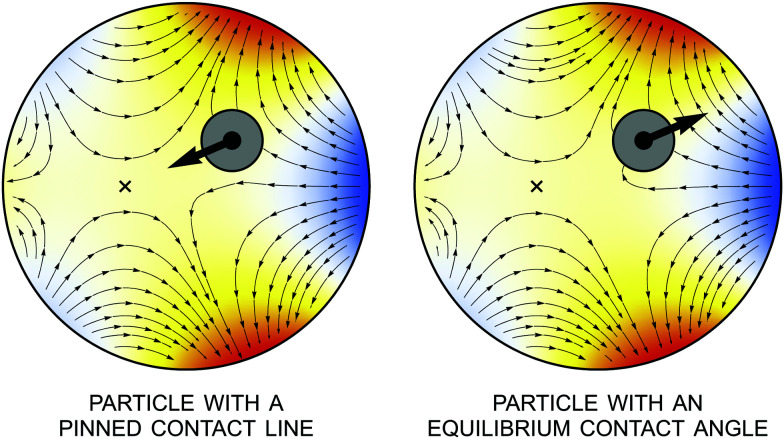
Interface height and gradient lines for a cylindrical particle with a symmetrically pinned contact line (left) or an equilibrium contact angle of 90° (right). Gradient lines are directed away from lower regions (*ζ* < 0, “cold” colors) towards higher regions (*ζ* > 0, “hot” colors). The thick arrow indicates the direction of the capillary force. The fixed point, indicated by a cross, is stable for the pinning condition and unstable for the equilibrium wetting condition [*cf.*eqn (4.25)].

Fig. 13 illustrates the interface height and gradient for pinning and equilibrium wetting conditions on the particle boundary. The fixed point ***r*** = ξ* is indicated by a cross, where ξ* is again given by eqn (4.25). This point attracts particles with pinned contact lines (stable) and repels particles with equilibrium contact angles (unstable). It is noteworthy that the gradient lines always terminate at the particle boundary, regardless of the wetting condition, because the particle rotation ***Ω*** is finite.

#### 4.2.2 Spherical particle

Würger^19^ derived the equilibrium wetting condition for a spherical particle with an arbitrary contact angle:4.30
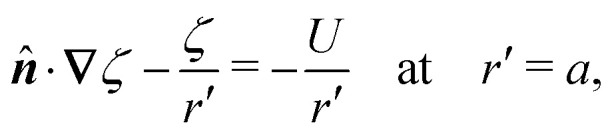
where the particle translation 
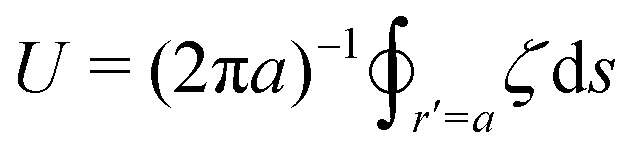
 is determined from the force-free condition (4.11). The torque-free condition (4.12) is automatically satisfied by (4.30), a consequence of the rotational invariance of spheres. Here, *a* denotes the lateral radius of the contact line in the undeformed interface (see Fig. 12, right). The radius of the sphere measured from its centroid is given by *a*/sin *α*, where *α* is the contact angle.

The Robin boundary condition (4.30) for spheres differs from the Neumann condition (4.26) for cylinders. The difference can be understood in the context of their 2D electrostatic analogues. Physically, eqn (4.26) specifies a distribution of transverse forces at the contact line, which have a one-to-one correspondence with electric charges. Thus, a cylindrical particle is akin to an electrical insulator with a prescribed boundary charge density such that its dipole moment vanishes. By contrast, eqn (4.30) constrains the lateral torque distribution at the contact line. This is analogous to a boundary distribution of splayed, radially oriented electric dipoles of equal strength. In this sense, spherical particles can be understood as uncharged insulators with a prescribed dipole density.

Eqn (4.8), (4.9), (4.11), and (4.30) may be straightforwardly solved for *ζ* using the method of reflections (see the ESI,† Section S.3.2.2). The quadrupole moment thus obtained,4.31
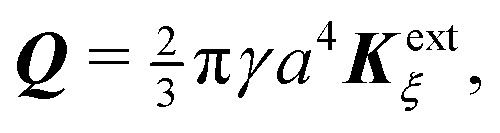
is one-third that found for a cylindrical particle of radius *a* [*cf.*eqn (4.27)]. The force ***F*** may then be calculated using either the stress-tensor integral (4.7) or the point-quadrupole formula 
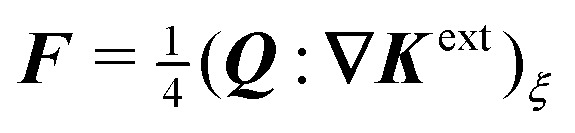
 to give4.32
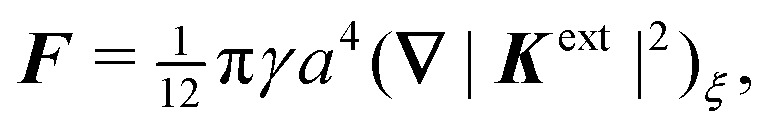
which is one-third the result (4.29) for cylinders.

**Fig. 14:**
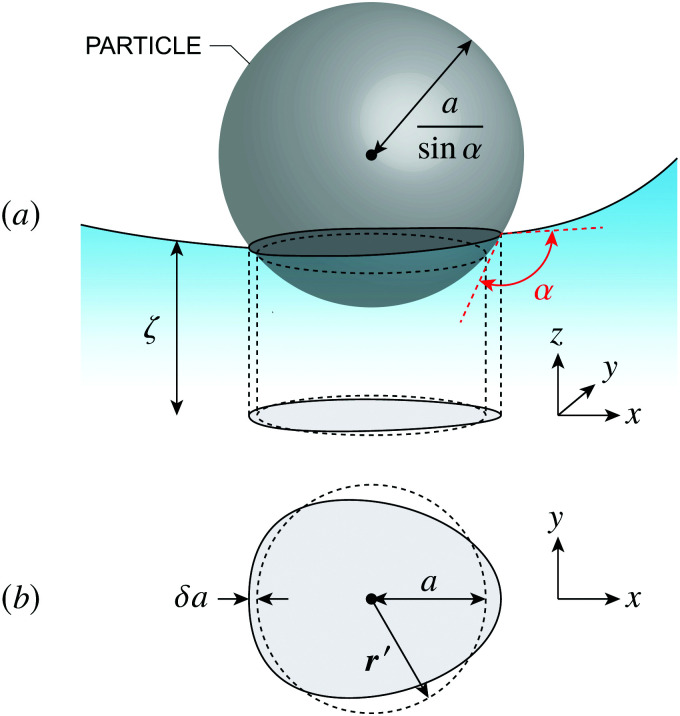
(a) 3D and (b) 2D illustrations of the contact-line distortion on a spherical particle with an equilibrium contact angle *α*. The radius *a* of the undeformed contact line is deformed to a new radius *a* + δ*a* [defined by eqn (4.33)] when the particle is embedded in an interface with an inhomogeneous, deviatoric curvature. The capillary work required to deform the contact line is given by eqn (4.36).

Calculating the energy is slightly more involved because the shape of the contact line on the particle is not necessarily circular. Recall that the energy *W* given by eqn (4.5) was derived assuming that the contact line was a perfect circle rotated out of the horizontal plane. For spherical particles, this is only true when the curvature of the interface vanishes everywhere (***K***^ext^ = **0**). When the interface is curved (***K***^ext^ ≠ **0**), then the contact line on the particle deforms in order to maintain a fixed contact angle *α* (Fig. 14). The radial distortion *δa* of the contact line is related to *a* and *ζ* by the equation,^19^

4.33 (*a* + δ*a*)^2^ = *a*^2^ − 2*a

<svg xmlns="http://www.w3.org/2000/svg" version="1.0" width="11.058824pt" height="16.000000pt" viewBox="0 0 11.058824 16.000000" preserveAspectRatio="xMidYMid meet"><metadata>
Created by potrace 1.16, written by Peter Selinger 2001-2019
</metadata><g transform="translate(1.000000,15.000000) scale(0.010294,-0.010294)" fill="currentColor" stroke="none"><path d="M400 1320 l0 -40 -40 0 -40 0 0 -40 0 -40 40 0 40 0 0 40 0 40 80 0 80 0 0 -40 0 -40 80 0 80 0 0 40 0 40 40 0 40 0 0 40 0 40 -40 0 -40 0 0 -40 0 -40 -80 0 -80 0 0 40 0 40 -80 0 -80 0 0 -40z M240 1080 l0 -40 40 0 40 0 0 -40 0 -40 40 0 40 0 0 -80 0 -80 -80 0 -80 0 0 -40 0 -40 -40 0 -40 0 0 -80 0 -80 -40 0 -40 0 0 -160 0 -160 40 0 40 0 0 -40 0 -40 160 0 160 0 0 -40 0 -40 -120 0 -120 0 0 -40 0 -40 120 0 120 0 0 40 0 40 40 0 40 0 0 80 0 80 -40 0 -40 0 0 40 0 40 -160 0 -160 0 0 80 0 80 40 0 40 0 0 120 0 120 80 0 80 0 0 40 0 40 40 0 40 0 0 80 0 80 120 0 120 0 0 40 0 40 -120 0 -120 0 0 -40 0 -40 -40 0 -40 0 0 80 0 80 -80 0 -80 0 0 -40z"/></g></svg>

 *cot *α*−**^2^,

where ** = (*ζ* − *U* − ***Ω***_×_·***r***′)|_*r*′=*a*_ is the height of the contact line in the co-translated and co-rotated frame.

**Fig. 15:**
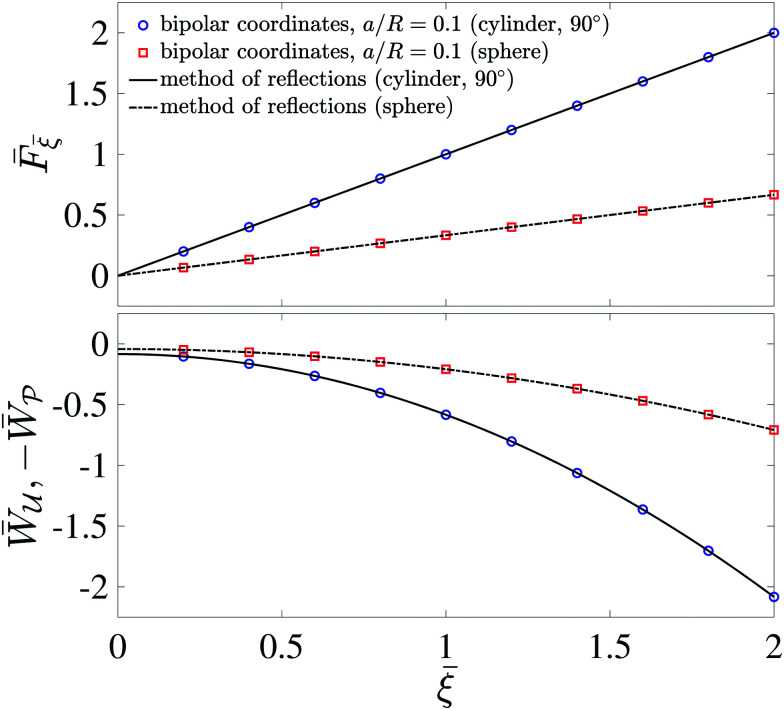
Capillary force (top) and energy (bottom) plotted against the relative particle position for cylindrical and spherical particles with equilibrium contact angles. The variables have the same meaning as in Fig. 11, assuming a common radius *a* of the undeformed contact line. The radius of the sphere is given by *a*/sin *α* for an arbitrary contact angle *α*; the cylinder is restricted to contact angles of 90°.

The distortion energy *W*_1_ associated with inserting the particle into the interface, without accounting for the deformation of the contact line, may be calculated using eqn (4.5). In the limit as *R* → ∞, we obtain4.34
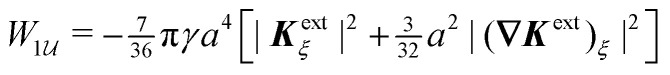
for a fixed distribution of heights on the outer boundary, or4.35
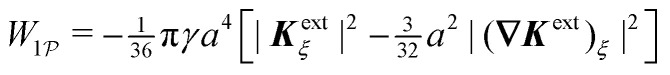
for fixed slopes. The additional work *W*_2_ required to deform the contact line from *r*′ = *a* to *a* + δ*a* is given by4.36
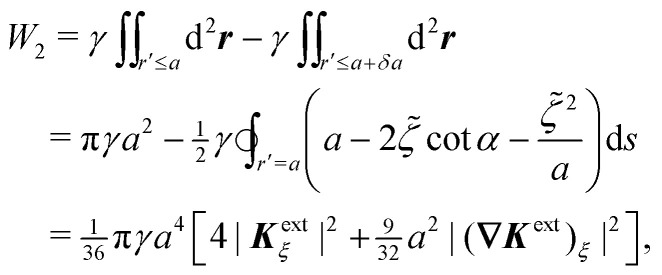
where we have used eqn (4.33) to eliminate δ*a*. Since the term linear in *ζ̃* integrates to zero, the result (4.36) is independent of the contact angle *α*. Summing the two energies *W* = *W*_1_ + *W*_2_ then gives4.37
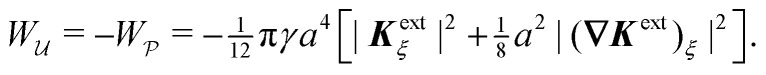
The last expression is the desired particle potential energy and may be compared against the analogous result (4.28) for cylinders. It is straightforward to show, using eqn (4.32) and (4.37), that the force and energy are related by the virtual work principle 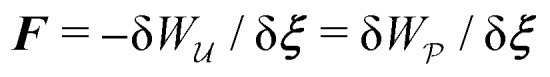, as was shown for cylinders [*cf.*eqn (4.29)]. A comparison of the force and energy for cylinders and spheres with equilibrium contact angles is shown in Fig. 15. Also plotted are exact results for *a*/*R* = 0.1 by means of eigenfunction expansions in bipolar coordinates (see the ESI,† Section S.4). Clearly, the bipolar solutions agree very well with the unbounded, analytical results, which neglect errors of *O*(*a*^4^/*R*^4^).

The first term on the right-hand side of eqn (4.37) – the part of the energy that depends on ***ξ*** – was derived first by Würger^19^ and later by other investigators.^20–22^ Although the magnitude of this term has recently become controversial in the literature,^19–28^ our calculations here – using both the stress tensor and the principle of virtual work – confirms Würger's original prediction. The key insight, as suggested by Würger himself,^26^ is to realize that the interfacial distortion produced by the spherical particle disturbs the static boundary condition at a faraway distance *R*. Enforcing this condition requires some work to be done; as we have shown, this work remains finite even as *R* → ∞. It follows that the capillary distortion energy *W* depends upon whether the heights (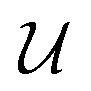) or slopes (
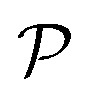
) are prescribed at *r* = *R*. The energies in these two cases are equal and opposite, as indicated by eqn (4.37), as a direct consequence of the fact that the particle is inert (for the contrary example of a “non-inert” particle, see Appendix E).

## 5 Discussion and conclusions

At the end of Section 3, we summarized classical results for cylindrical conductors and insulators by writing the general expression for the dielectrophoretic force,

5.1 ***F*** = (***P***·**∇*E***^ext^)_***ξ***_ = π*εfa*^2^(**∇**|***E***^ext^|^2^)_***ξ***_,

where ***P*** = 2π*εfa*^2^***E***^ext^_***ξ***_ is the electric dipole moment and *f* is the 2D Clausius–Mossotti factor that ranges from −1 (for ideal insulators) to 1 (for ideal conductors). Based on our results from Section 4, we may write a similar expression for the capillary force on an interface-trapped colloid:5.2
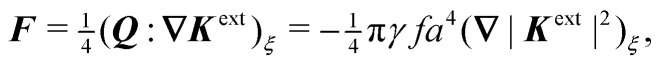
where ***Q*** = −2π*γfa*^4^***K***^ext^_***ξ***_ is the capillary quadrupole moment and *f* = 1 for a particle with a circularly pinned contact line, −1 for a cylindrical particle with a 90° contact angle, and 
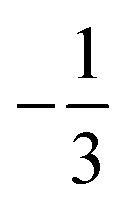
 for a spherical particle with an arbitrary contact angle. These expressions clearly show the analogy between dielectrophoresis and the motion of inert colloids in a curved interface. To calculate the velocity of the overdamped motion, one need only equate the force ***F*** to the drag exerted on the particle by the surrounding media.

Both eqn (5.1) and (5.2) can be expressed as the gradient of a potential energy by use of the principle of virtual work. In the electric problem, the correct potential energy is 
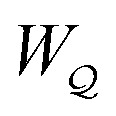
 – the electrical energy needed to insert the particle into a fixed distribution of *charges*. In practice, it is more common to establish the field using a configuration of electrodes maintained at fixed *potentials*. Then, the associated energy 
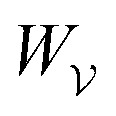
 is *not* equivalent to the particle's potential energy. It is well known in the electrostatics literature^77,80^ that some extra work 
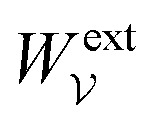
 must be done (*e.g.*, by a system of batteries) to move charges onto or off of the electrodes, and this work must be excluded from the total electrical energy 
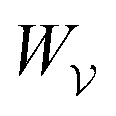
. For non-polar particles, the batteries must do 
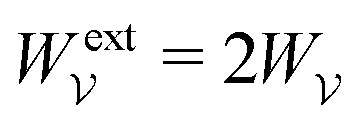
 amount of work and the correct potential energy (for fixed potentials) is 
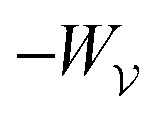
.

In the capillary problem, we showed that fixing the interface height at a boundary has a similar effect to fixing the electric charge density. This result is somewhat surprising, since the interface height *ζ* is the mathematical analogue of the electric potential *ψ*. Nevertheless, there is a physical basis for it. If the interface heights are fixed at the outer boundary enclosing the particle, then the adhesion energy remains constant as the particle moves through the interface, and the only change to the capillary energy is due to particle-sourced interfacial distortions. Hence, we identified 
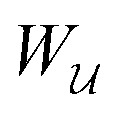
 as the particle's potential energy. By analogy to the electric problem, fixing the outer *slopes* results in a different energy 
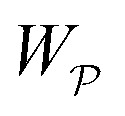
. We showed that the difference between the two energies is associated with the Young–Dupré work of adhesion 
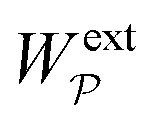
 needed to alter the wetted area on the outer boundary. For inert colloids, 
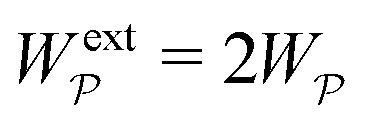
 and the correct potential energy (for fixed slopes) is 
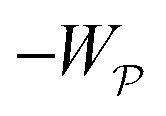
. Of course, it is typically easier to create a fluid–fluid interface in the laboratory by fixing the interface *heights*, provided the wall of the container has a well defined edge.

**Fig. 16:**
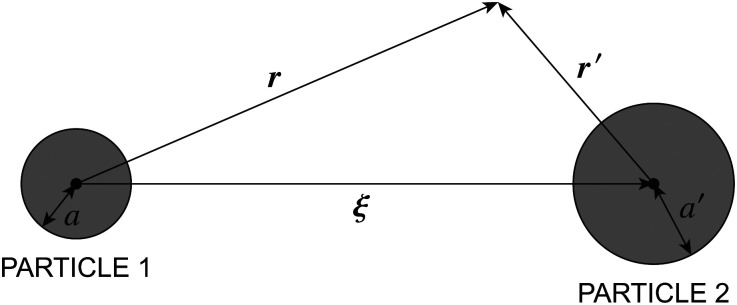
Two circular particles of radii *a* and *a*′ with center-to-center separation ***ξ*** = ***r*** − ***r***′.

An important implication of these results is that two interface-trapped particles will attract each other if the interface has a finite deviatoric curvature, even if the particles themselves do not create distortions within a planar interface. To see this explicitly, it is useful to apply eqn (5.2) to a pair of particles. Fig. 16 illustrates two circularly symmetric particles of radii *a* and *a*′ that are well separated, *i.e.*, their center-to-center separation ***ξ*** is large in comparison to their radii. For simplicity, we assume the particles are embedded in an interface with constant curvature ***K***^ext^_**0**_. In the limit of infinite separation *ξ* → ∞, the external curvature induces the quadrupole moments ***Q*** = −2π*γfa*^4^***K***^ext^_**0**_ and ***Q***′ = −2π*γf*′*a*′^4^***K***^ext^_**0**_ on the first and second particle, respectively. For finite but large separations, the total curvature ***K***^ext^ that is “felt” by the second particle is, to a first approximation, the sum of the host-interface curvature ***K***^ext^_**0**_ and the quadrupolar distortion produced by the first particle:5.3
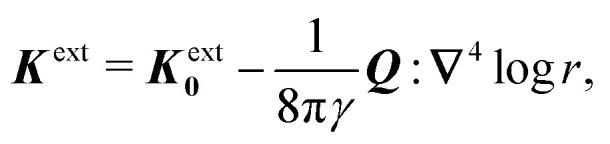
where ***r*** is the position measured from the center of the first particle. Then, the lateral force on the second particle (at ***r*** = ***ξ***) due to the presence of the first (at ***r*** = **0**) is, to the quadrupolar level of approximation,5.4
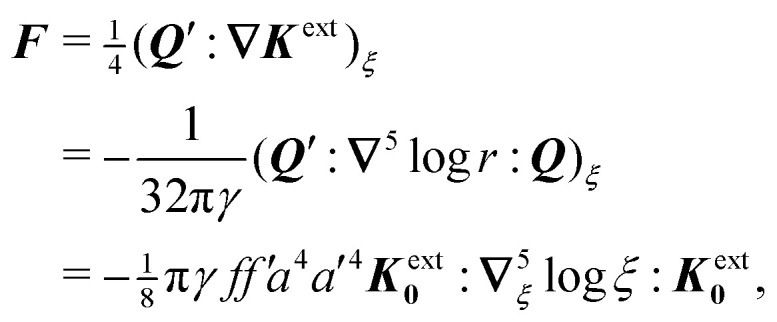
where the errors neglected are of *O*(*a*^4^/*ξ*^4^,*a*′^4^/*ξ*^4^). By symmetry arguments, the force exerted on the first particle by the second is equal and opposite to eqn (5.4). This force scales like ∼1/*ξ*^5^ (corresponding to a potential energy ∼1/*ξ*^4^) as determined by previous investigators.^19,29,30^

In the pair problem just described, the transverse torque on each particle vanishes, *T* = 0. This is because (i) the particles are circularly symmetric and (ii) their induced quadrupole moments are always aligned with the local curvature [this can also be proven rigorously, by applying eqn (D.11) in Appendix D]. However, the net torque on a pair (or, more broadly, a cluster) of particles is non-zero, in general, because the force ***F*** on the individual particles is not necessarily parallel to their pair separation ***ξ***. The transverse torque on a particle pair acts to rotate ***ξ*** until it is aligned with a principal curvature (*i.e.*, parallel to an eigenvector of ***K***^ext^_**0**_), at which point the torque vanishes. Once aligned, ***K***^ext^_**0**_ = −*Dξ*^2^**∇**^2 ^_***ξ***_log *ξ* = *D*(2***ξξ***/*ξ*^2^ − ***δ***) and the force on each particle [eqn (5.4)] simplifies to5.5
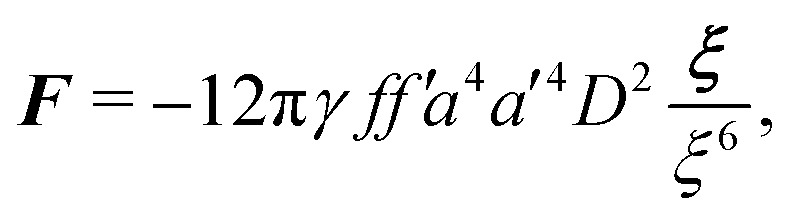
where 
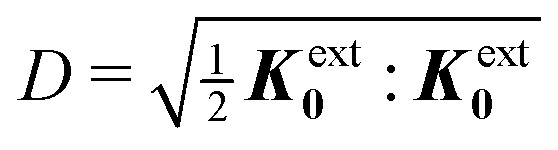
 is the deviatoric curvature [defined in Appendix B, eqn (B.10)]. According to eqn (5.5), particle pairs will attract if their form factors *f* and *f*′ share the same sign. Conversely, a particle with a pinned contact line (*f* > 0) will repel a particle with an equilibrium contact angle (*f*′ < 0).

Our findings confirm a type of capillary attraction between “inert” particles that was originally discovered by Würger^19^ for spherical particles with equilibrium contact angles. In fact, we have extended his prediction to explain the force of attraction between particles with planar, pinned contact lines and cylindrical particles with 90° contact angles. Moreover, our calculations reveal the source of inconsistency that led Sharifi-Mood and coworkers^23,24^ to conclude, incorrectly, that particles with planar, non-undulated contact lines would not migrate in a curvature gradient. The issue, which was correctly identified by Würger^26^ in a subsequent comment, is in the treatment of the outer boundary enclosing the particle, which is ultimately responsible for establishing the curvature of the host interface. Although this boundary might be sufficiently far from the particle so as not to affect its motion, we have demonstrated (specifically, in Section 4.1.2) that the capillary energy is not independent of the far-field wetting condition. If one properly accounts for this wetting condition, as we have done, then it is clear that the distortion energy is finite and the lateral force predicted by a virtual work argument is exactly that given by integrating the stress tensor (4.6) over the particle boundary.

Regarding the energy calculation, two points are worthy of emphasis. First, as a general rule, it is often preferable to use the principle of virtual work to derive interparticle forces in a many-body system in lieu of integrating the stress tensor over the particle boundaries. Although the stress tensor method is the more “direct” approach, it is computationally infeasible to apply it to a system containing anything more than two particles. Comparatively, it is a much simpler matter to compute the energy *W* for a many-body system. (This was done, for instance, by Bonnecaze and Brady^87^ in their simulation of dielectric particle suspensions interacting through dielectrophoretic and hydrodynamic forces.) Consequently, it is crucial that the energy *W* is accurately calculated; to this end, we hope our study clarifies the subtle, yet essential, role played by boundaries in such calculations. As was mentioned earlier, this subtlety was also identified by Domínguez *et al.*^29^ and later by Würger^26^ in a qualitative sense. Our work systematically develops their arguments, on a rigorous and quantitative basis, for a single particle trapped at a curved, but bounded, interface, thereby illustrating the effect of the boundary on the capillary energy.

The second point we wish to emphasize is the connection between dielectrophoresis and the interfacial migration of inert colloids, and its important implications for the work done on a moving particle. Our central argument is that the verified^32^ existence of dielectrophoretic forces on cylindrical, non-polar bodies implies that there must be an analogous capillary force on an interface-trapped, inert colloid, and *vice versa*. Put another way: if an induced capillary quadrupole does not lead to particle migration in a curvature gradient, *it would imply that 2D dielectrophoresis does not exist!* In fact, this very conclusion was reached, albeit unintentionally, in a recent review by Liu *et al.*^2^ (Sectin 5.1.1 of their article). There, they argued that embedding a conducting particle in a dielectric medium with an applied electric field requires no electrical work. Our calculations in Section 3.1 directly refute this conclusion; again, the discrepancy can be explained by considering the electrostatic condition in the far field.

Interestingly, a similar problem also emerges in 3D dielectrophoresis. For a spherical conductor of radius *a* placed in a linear field set up by fixed charges, classical arguments^77^ show that the electrical energy in 3D is5.6
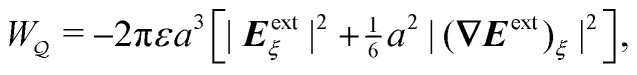
which may be compared to the analogous result (3.17) in 2D. On the other hand, if the electrostatic condition on the outer boundary is neglected, then one obtains instead5.7
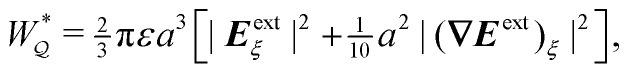
which is non-zero but different from the correct result (5.6). Eqn (5.7) mistakenly suggests that a conducting particle is forced in the opposite direction (against the field gradient) with one-third the strength. It should be recalled that the analogous 2D energy was 
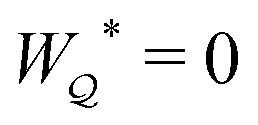
 when the outer boundary condition was neglected. Thus, the 2D problem is, in some sense, pathological. It is straightforward to verify that eqn (5.6), not (5.7), gives the correct dielectrophoretic force^33,34,39^ upon differentiation with respect to ***ξ***:5.8
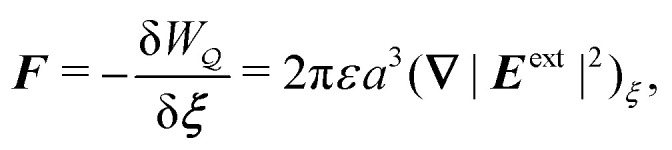
which is the 3D analogue of (3.20).

Although our study is the first, to the best of our knowledge, to explicitly consider an outer shell enclosing an interface-trapped particle, all of the results presented in the main text apply to the unbounded limit *R* → ∞. The first effect of the particle–shell interaction due to an induced capillary quadrupole contributes an *O*(*a*^4^/*R*^4^) correction to the capillary force and energy. These corrections are summarized in Table S.2 of the ESI† for different types of particles and shell wetting conditions. In the electric problem, the analogous finite-size correction is of *O*(*a*^2^/*R*^2^) (see Table S.1, ESI†) due to the slower decay of the dipolar interaction. All of the analytical results for finite values of *a*/*R* were computed using the method of reflections and verified by comparing against an exact solution in bipolar coordinates (see Fig. S.1–S.10, ESI† for the comparisons).

Besides accounting for the finite size of the domain, a number of other physical forces may be introduced to ensure that the energy remains finite. One such resolution was offered by Galatola,^25^ who suggested that the small-slope approximation |**∇***ζ*| ≪ 1 does not apply far from the particle. He argued that, because the curvature of the interface is assumed to be everywhere finite, the interface slope |**∇***ζ*| diverges in the far field. In this region, Laplace's equation ∇^2^*ζ* = 0 is no longer a suitable approximation of the exact governing equation5.9
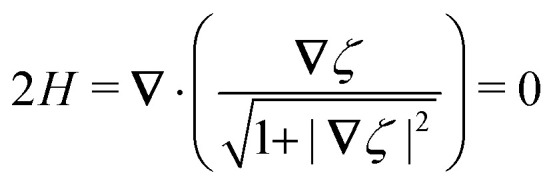
for the shape of a static interface under zero pressure. Here, *H* is the mean curvature defined in Appendix B [eqn (B.8)]. Eqn (5.9) shows that the curvature 2*H* remains bounded even as |**∇***ζ*| → ∞. For Laplace's equation to hold within the domain of interest, the shell radius *R* must be small compared to the characteristic radius of curvature of the interface. Assuming the host-interface curvature is given by eqn (4.2), this yields the criteria |***K***^ext^_**0**_| ≪ 1/*R* and |(**∇*K***^ext^)_**0**_| ≪ 1/*R*^2^. If these conditions are not met, then the exact curvature 2*H* must be used in place of its small-slope approximation ∇^2^*ζ*. This also means that the expressions for the capillary energy *W*, force ***F***, and torque *T* must be modified to account for finite interface slopes.

Another, perfectly reasonable possibility is the force of gravity exerts a hydrostatic pressure on the interface to weigh it down in the far field. Gravitational forces were neglected in Section 4. In the small-slope limit, the addition of hydrostatic pressure transforms Laplace's equation into^88^5.10
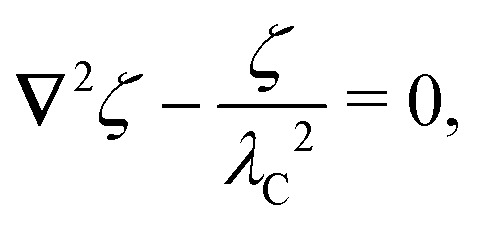
where 
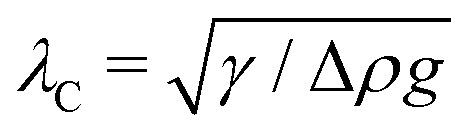
 is the capillary length, Δ*ρ* is the difference in (mass) density between the two fluids, and *g* is the acceleration due to gravity. For gravity to be safely neglected, the size of the container must be smaller than the capillary length: *R* ≪ *λ*_C_. For typical working fluids, this means that the shell radius *R* must be smaller than a few millimeters (*e.g.*, for a water–air interface, *λ*_C_ = 2.7 mm). If this condition is not met, then the hydrostatic pressure [the second term in eqn (5.10)] causes exponential decay of particle-sourced interfacial distortions at large distances *r* = *O*(*λ*_C_). At that point, the disturbance created at the outer boundary is immaterial. The electrostatic analogue of a hydrostatic pressure is an electrolytic medium with *N* mobile charge carriers. In the high-temperature limit (*kT*/*e* ≫ *z*_*j*_*ψ*), the governing equation for *ψ* then becomes^89^5.11
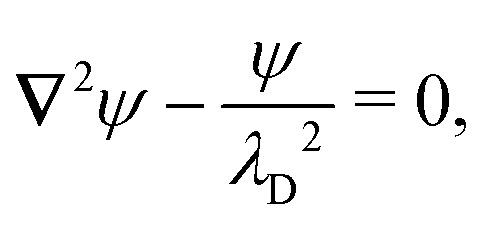
where 
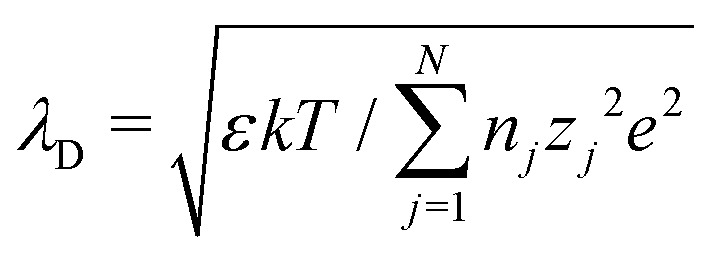
 is the Debye screening length, *e* is the elementary charge, and *n*_*j*_ and *z*_*j*_ denote, respectively, the number density and valence of the *j*th charge carrier. Eqn (5.11) states that the electric field is exponentially screened at distances *r* = *O*(*λ*_D_). For screening effects to be safely neglected in the electric problem (Section 3), we must have *R* ≪ *λ*_D_.

Finally, it bears mentioning that there are other analogies to capillary statics, besides 2D electrostatics, that may be useful to gain physical insight. There is, of course, historical precedent^77^ for the association of Poisson's equation (the inhomogeneous form of Laplace's equation) to problems of electrostatics. However, Poisson's equation in 2D governs a wide variety of other physical phenomena.^90^ As an example, both electrostatics and magnetostatics share a common mathematical structure in 2D. Consequently, either one of these can be made analogous to capillary statics through a suitable change of variables (see Table 1, as well as footnote 28 in the article by Domínguez *et al.*^29^). Of particular relevance to the present work is the one-to-one correspondence between *dielectrophoresis* and *magnetophoresis*.^39^ In fact, the latter phenomenon is more commonplace: a paper clip sitting on a table will not move on its own, but placing a bar magnet nearby magnetizes the paper clip, pulling it towards the bar magnet. From a pedagogical perspective, there is greater value in an analogy that is more immediately familiar. On that basis alone, we could have written this entire article in terms of a physical analogy to magnetophoresis, instead of dielectrophoresis. We chose to focus on the latter, due to the already well-established^2,29–31^ connection between capillary statics and electrostatics in the literature.

**Table 1:** Physical analogies between variables in capillary statics, electrostatics, and magnetostatics in 2D. Here, *γ* is the interfacial tension, *p* is the capillary pressure, *ζ* is the interface height, and 
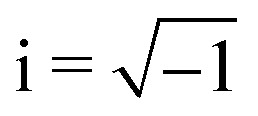
 is the imaginary unit

Electrostatics		Magnetostatics	
Permittivity	*ε* → *γ*	Permeability	*μ* → 1/*γ*
Charge density	*q* → i*p*	Current density	*J* → *p*
Electric potential	*ψ* → i*ζ*	Magnetic potential	*A* → *ζ*
Electric field	***E*** → − i**∇***ζ*	Magnetic field	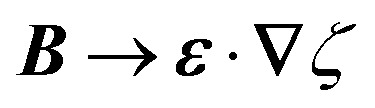

There are other reasons, besides greater familiarity, for why other physical analogies should be explored. As was discussed near the beginning of this section, there are subtle differences between capillary statics and electrostatics that emerge when evaluating the energy *W*. However, these differences are not ubiquitous among other physical analogies. By way of illustration, consider the magnetic work required to move a paramagnetic particle through a magnetic field ***B***^ext^. The potential energy of the particle is equivalent to the magnetic energy *W* of the configuration, provided that the magnetic potential *A* is fixed on the boundaries that establish the field.^77,78^ However, in the analogous problem of calculating the electric work done on a dielectric particle moving through an electric field ***E***^ext^, it is the electric charge density −*ε*(***n̂***·**∇***ψ*), not the electric potential *ψ*, that must be fixed on the boundaries. As it turns out, the problem of fixed potentials *ψ* in electrostatics is analogous to fixed currents *J* in magnetostatics (see Jackson,^77^ p. 214). Turning to the capillary problem, one immediately concludes that the interface height *ζ* plays a similar role to the magnetic potential *A*, and, furthermore, the two are mathematical analogues according to Table 1. In this sense, the analogy between capillarity and magnetism is, surprisingly, more complete than the analogy to electricity.

## Conflicts of interest

There are no conflicts to declare.

## Supplementary Material

SM-017-D0SM02143A-s001

## Appendices

### A Partial integration of the energy integral

In this appendix, we illustrate how to integrate eqn (3.5) and (4.5) by parts such that the line integral at *r* = *R* vanishes, resulting in eqn (3.17), (3.18), (4.18) and (4.21) in the main text. Essentially the same approach is used by Jackson^77^ (pp. 166–167) and by Landau and Lifshitz^78^ (pp. 52–53) in order to evaluate the electrical energy.

We consider two harmonic functions *f*(***r***) and *g*(***r***) defined within the geometry of Fig. 3, and denote the integral *I* by

A.1

Such an integral appears in eqn (3.5) and (4.5) with (*f*,*g*) = (*ψ*,*ψ*^ext^) and (*ζ*,*ζ*^ext^), respectively. A clever factorization of the integrand yields the following, equivalent representation:

A.2

Here the integral over *r* ≤ *R* has been broken into two parts, one evaluated over the particle region and the other over the interstitial region bounded by the particle and the shell.

Eqn (A.2) may be integrated by parts in one of two ways. In the first approach, the gradient of (*f* + *g*) in the first term is integrated, giving

A.3

where we have used ∇^2^*f* = ∇^2^*g* = 0 to eliminate the area integrals, leaving only line integrals at the particle and shell boundaries. If ***n̂***·**∇***f* = ***n̂***·**∇***g* at *r* = *R*, then the integral over the shell boundary vanishes. This leads directly to eqn (3.17) and (4.21) in the main text.

On the other hand, if we integrate the gradient of (*f* − *g*) in the first term of eqn (A.2), we obtain instead

A.4

Here, the second integral vanishes if *f* = *g* at *r* = *R*, resulting in eqn (3.18) and (4.18) in the main text. Thus, although eqn (A.3) and (A.4) are entirely equivalent, they are respectively preferred in situations where Neumann and Dirichlet conditions are applied to the outer boundary.

### B Differential geometry of a Monge patch

In the Monge gauge, the three-dimensional (3D) position of a surface is given by ***r*** + *ζ*(***r***)*ẑ*, where ***r*** is the horizontal position, *ζ*(***r***) is the vertical position of the surface at ***r***, and *ẑ* is the unit vector in the vertical direction. In keeping with the notation of the main text, we denote by ***δ*** the two-dimensional (2D) unit tensor in the horizontal plane. Below, we express the differential geometry of a Monge patch for small slopes |**∇***ζ*| ≪ 1, retaining terms up to quadratic order in |**∇***ζ*|.

In local polar coordinates (*r*,*θ*), the covariant tangential vectors of the surface are

B.1a

B.1b

where ***ẑ*** × ***r*** = *r****θ̂*** is the vector tangent to a circle of radius *r*. In order to form a complete covariant basis, we define the unit normal  to the surface as

B.2

where the last line is an approximation for small slopes |**∇***ζ*| ≪ 1. The surface metric *J* is defined as the scalar triple product of the covariant basis vectors scaled with respect to the radius *r*:

B.3

Thence, we may also define the contravariant tangential vectors *via* the reciprocal relations,

B.4a

B.4b

where we have used *ẑ*·***r*** = 0. The vector line element along an arbitrary contour *Γ* tangent to the surface is given by

B.5

Likewise, the area element of an arbitrary surface patch *Σ* is

B.6

The curvature tensor of the surface is defined with respect to the gradient of the surface normal as

B.7

For all intents and purposes, the *O*(|**∇***ζ*|^2^) terms in eqn (B.7) are of little consequence and may be neglected, giving ***K*** ≃ **∇∇***ζ*. The principal scalar invariants of ***K*** include the trace

B.8

and the sum of principal minors

B.9

(The determinant of ***K*** vanishes identically, det ***K*** = 0.) Here, *H* and *K* denote the mean and Gaussian curvatures, respectively. The deviatoric curvature *D* is related to *H* and *K* by the relation

B.10

The sign of *D* is a matter of convention, depending on the direction of the surface normal . It is worth noting that eqn (B.8)–(B.10) are accurate up to *O*(|**∇***ζ*|^3^) errors.

For interfaces under zero static pressure, a balance of forces normal to the interface shows that the mean curvature *H* must vanish locally (see, for instance, Section 60 of Landau and Lifshitz^91^). In the small-slope limit, eqn (B.87) thus gives

B.11 ∇^2^*ζ* = 0,

which is Laplace's equation. Substituting eqn (B.11) into (B.10) gives the deviatoric curvature  for a static interface.

### C Capillary distortion energy

Eqn (4.5) for the capillary distortion energy *W* was presented in the main text without derivation. In this appendix, we derive this expression starting from a general representation for the interface (interfacial tension *γ*) surrounding an embedded particle with a circular contact line (radius *a*) and bounded externally by a circular shell (radius *R*).

If the interface were initially flat, then inserting the particle creates no distortion and the energy cost is given by the “trapping energy,”

C.1

For a curved interface with initial height *z* = *ζ*^ext^(***r***), inserting the particle distorts the interface to a new height *z* = *ζ*(***r***;***ξ***). We denote by *Σ* and *Σ*_0_ the set of contiguous points occupied by the interface and the particle, respectively. Then, using the area element defined by eqn (B.6), we may write the new energy cost as

C.2

where the second line is an approximation up to quadratic order in the interface slopes |**∇***ζ*| and |**∇***ζ*^ext^|. For the first two integrals in eqn (C.2), the integrands are sufficiently small such that the regions of integration may be transferred to the undeformed interface plane:

C.3

C.4

The same action cannot be taken for the for the last integral in eqn (C.2) because the integrand is of *O*(1). However, we may apply the divergence theorem to transform the area integral over *Σ*_0_ into a line integral over the bounding contour *Γ*:

C.5

To transfer the contour of integration to *r*′ = *a*, we must account for the small distortion of the contact line. For circular contact lines, the only such distortion is due to a rotation ***Ω*** out of the horizontal plane (see Fig. 7). Up to quadratic order in ***Ω***, eqn (C.5) may be approximated as

C.6

where the perturbations *δ****r***′, *δ****n̂***, and *δ*d*s* are proportional to the dyad  and ***ε*** is the 2D alternating tensor). Applying the differential geometry of curves, we find

C.7

C.8

C.9

where  is the unit tangent to the contour (the intermediate algebra leading to these expressions is available from the authors upon request). After substituting eqn (C.7)–(C.9) into (C.6) and setting ***r***′ = *a****n̂*** at *r*′ = *a*, we obtain

C.10

Summing eqn (C.3), (C.4), and (C.10) then gives

C.11

It is worth noting that the first integral in eqn (C.11) contributes a term  that exactly cancels the last term  arising from the boundary distortion. Thus, the constant part of the energy *W*_1_ is −π*γa*^2^, as expected for circularly symmetric particles.

Subtracting the trapping energy *W*_0_ [eqn (C.1)] from *W*_1_ [eqn (C.11)] then gives the energy solely associated with the distortion of the interface:

C.12

which is equivalent to eqn (4.5) in the main text. For non-circular contact lines, additional terms must be added to eqn (C.12) to account for the deformation of the contact line in the co-rotated frame (*e.g.*, see Section 4.2.2 for a discussion of spherical particles with equilibrium contact angles).

### D Capillary force and torque

In this appendix, we derive the force and torque on an interface-trapped particle due to the nonlinear curvature-capillary interaction. Some of this material can be found in the articles by Domínguez *et al.*,^29^ Fournier,^92^ and Galatola;^93^ however, for the sake of clarity, it is worth revisiting the derivation.

Our starting point is an expression for the force d***f*** due to interfacial tension *γ* acting on a line element d***ℓ*** of an arbitrary contour *Γ*. Using elementary geometry, we may write

D.1 d***f*** = −*γ****v̂*** × d***ℓ***,

where  is the unit normal to the interface as defined in Appendix B. Substituting eqn (B.5) for d***ℓ*** and using the reciprocal relations (B.4), we find

D.2

The last expression may be simplified further *via* the substitutions

D.3

and

D.4

[equivalently, eqn (4.6)] to get

D.5 d***f*** ≃ [*γ****n̂*** + *γ*(***n̂***·**∇***ζ*)***ẑ*** + ***n̂***·***σ***]d*s*.

The first (constant) term on the right-hand side of eqn (D.5) represents the force exerted on the contour in the absence of interfacial curvature. The second (linear) term is of *O*(|**∇***ζ*|) and will be shown, below, to contribute to the transverse force and lateral torque on the particle. The final (quadratic) term is of *O*(|**∇***ζ*|^2^) through the capillary stress tensor ***σ***, and will be shown to contribute to the lateral force and transverse torque.

To calculate the total force on the particle, we integrate eqn (D.5) over the contact line *Γ*:

D.6

where we have used . The horizontal component of eqn (D.6) is

D.7

where ***F*** is the horizontal force exerted *on* the particle *by* the interface. eqn (D.7) is equivalent to (4.7) in the main text with the substitution (4.6) for the stress tensor ***σ***. The vertical component of eqn (D.6) is

D.8

where *P* denotes the vertical force exerted *on* the interface *by* the particle. Hence, a positive pressure force *P* displaces the particle above the undeformed interface plane. For a circularly symmetric particle, the resulting interface profile is a capillary monopole (Fig. 1a):

D.9

For the monopole to vanish, we must have *P* = 0 [eqn (4.11)].

To calculate the torque on the particle, we take the cross product of the position (measured from the particle's center at ***r*** = ***ξ***, *z* = *U*) with the differential force element (D.5) and integrate over the contact line:

D.10

where we have used ***r***′ = ***r*** − ***ξ*** and . For a general vector ***A*** in the horizontal plane, we define the orthogonal vector . Then, taking the vertical component of eqn (D.10) gives

D.11

The transverse torque *T* exerted *on* the particle *by* the interface is non-zero only if the particle has inherent orientation that couples to the background curvature [see, for example, eqn (E.7) in Appendix E]. For circularly symmetric, “inert” colloids, no such orientation exists and we have *T* = 0. The horizontal component of eqn (D.10) is given by

D.12

where  denotes the horizontal torque exerted *on* the interface *by* the particle. A positive torque ***N***, *e.g.*, due to a polarized pressure distribution on the particle face, rotates the particle out of the plane. The simplest of such deformations is the capillary dipole (Fig. 1b):

D.13

For the dipole to vanish, we must have ***N***_×_ = **0** [eqn (4.12)].

For mechanically isolated particles with vanishing monopole moment (*P* = 0) and dipole moment (***N***_×_ = **0**), the lowest-order multipole moment is the (symmetric and traceless) quadrupole moment ***Q***, which is given by eqn (4.13) in the main text. A pure capillary quadrupole centered at the particle admits the interface profile (Fig. 1c),

D.14

### E Effect of a static contact-line undulation

Stamou *et al.*^30^ and later Sharifi-Mood *et al.*^23,24^ showed that a static undulation of the contact line causes a particle to migrate towards regions of high curvature. Such transverse undulations are neglected in the analysis presented in Section 4.1, but we briefly consider their effect here. Approximating the static undulation as quadrupolar (see Fig. 1 of Stamou *et al.*^30^), the boundary condition (4.10) is modified to read,

E.1

where ***Π*** is a symmetric and traceless tensor. If eqn (E.1) is satisfied, then the new quadrupole moment of the particle given by

E.2 ***Q*** = 2π*γa*^4^(***Π*** − ***K***^ext^_***ξ***_),

instead of eqn (4.16). Thus, the contact-line undulation creates a permanent quadrupole moment 2π*γa*^4^***Π*** that can oppose the induced quadrupole moment −2π*γa*^4^***K***^ext^_***ξ***_. The natural electrostatic analogue of an interface-trapped particle with an undulated contact line is an “electret” (a permanently polarized dielectric) in an electric field.

Following the same approach used to derive eqn (4.18), we may calculate the capillary energy for fixed heights on the outer shell:

E.3

Compared to eqn (4.18), (E.3) contains additional terms proportional to ***Π***. The crucial coupling term ***Π***:***K***^ext^_***ξ***_ results in both a force and torque on the particle (discussed below). Since the wetted area on the outer shell remains fixed, no additional energy is supplied to the system: . Thus, we identify  as the particle's potential energy in an external curvature.

Similarly, the energy while holding the outer slopes fixed is

E.4

which may be compared to eqn (4.21). Importantly, the coupling term ***Π***:***K***^ext^_***ξ***_ does not appear in eqn (E.4). Thus, we find , contrary to the result for a symmetrically pinned contact line. Physically, the undulation of the particle's contact line can be regarded as a source of force density (the capillary analogue of an electric charge density) that contributes additional work when the interface height on the outer shell is not fixed. Indeed, if we calculate the Young–Dupré work of adhesion needed to displace the outer height, we find

E.5

which may be compared to eqn (4.23). Subtracting eqn (E.5) from (E.4) gives , *i.e.*, the particle's potential energy.

In their analysis of particles with undulated contact lines, Sharifi-Mood *et al.*^23,24^ did not enforce the wetting condition on an outer enclosing boundary. Consequently, they derived an energy  that differs from the correct energy (E.3) in that the coupling term ***Π***:***K***^ext^_***ξ***_ is halved while the terms containing |***K***^ext^_***ξ***_|^2^ and |(**∇*K***^ext^)_***ξ***_|^2^ vanish altogether. This error was correctly identified by Galatola.^25^ For practical purposes, the wrong prefactor of the coupling term ***Π***:***K***^ext^_***ξ***_ has little consequence because the amplitude of ***Π*** is typically fit to experimental data. Thus, the only modification when using the formulas of Sharifi-Mood *et al.*^23,24^ is that the fit values of ***Π*** must be doubled.

By applying a virtual work principle to eqn (E.3), one derives the lateral force on the particle,

E.6

which again satisfies the point-quadrupole formula  with ***Q*** given by (E.2). (Note that  in this case!) If ***Π*** and ***K***^ext^ are aligned, then the force due to the permanent quadrupole acts against the induced quadrupole, driving the particle towards regions of high curvature.

One may also use a virtual work argument to derive the transverse torque *T* on the particle. Unlike “inert” particles, which have no intrinsic orientation, particles with undulated contact lines have a preferred director ***p***. Expanding the quadrupolar amplitude as ***Π*** = 2***pp*** − *p*^2^***δ***, the torque on the particle is directly given by the virtual work principle,

E.7

which, by use of eqn (E.2), simplifies to . This form is equivalent, save for a factor of two, to eqn (40) in the article by Domínguez *et al.*^29^ According to eqn (E.7), the torque acts to align the director ***p*** with the principal curvature of the host interface. When *T* = 0, then ***Π*** and ***K***^ext^_***ξ***_ are aligned and the permanent and induced quadrupoles act in opposition to each other.

For the contact-line undulation to be safely neglected, its quadrupolar amplitude must be small compared to the local curvature: |***Π***| ≪ |***K***^ext^_***ξ***_|. For an interface with characteristic deviatoric curvature *D*, we have |***K***^ext^_***ξ***_| ∼ *D*. According to the particle boundary condition (E.1), the quadrupolar amplitude scales as ***Π*** ∼ *ζ̃*/*a*^2^, where *ζ̃* = (*ζ* − *U* − ***Ω***_×_·***r***′)|_*r*′=*a*_ is the height of the particle-sourced undulation. This gives a criterion *ζ̃* ≪ *a*^2^*D* for the permanent quadrupole to be subdominant. To be quantitative, we assume a typical particle radius *a* ≃ 5 μm and deviatoric curvature *D* ≃ 1 mm^−1^, which are characteristic of the conditions used in the studies by Sharifi-Mood *et al.*^23,24^ Then, for the permanent quadrupole to be subdominant, the contact-line undulation *ζ̃* must be much smaller than *a*^2^*D* ≃ 25 nm, which is commensurate with surface roughnesses measured by Sharifi-Mood *et al.*^23,24^ using atomic force microscopy. Assuming the contact-line undulation (*ζ̃*) is fixed, the ratio |***Π***|/|***K***^ext^_***ξ***_| can be reduced either by increasing the particle radius *a* (while maintaining a small buoyant weight) or by increasing the deviatoric curvature *D* of the host interface.

## References

[cit1] Deshmukh O. S., van den Ende D., Stuart M. C., Mugele F., Duits M. H. G. (2015). Adv. Colloid Inteface Sci..

[cit2] Liu I. B., Sharifi-Mood N., Stebe K. J. (2018). Annu. Rev. Condens. Matter Phys..

[cit3] Bowden N., Terfort A., Carbeck J., Whitesides G. M. (1997). Science.

[cit4] Bowden N., Choi I. S., Grzybowski B. A., Whitesides G. M. (1999). J. Am. Chem. Soc..

[cit5] Bowden N., Arias F., Deng T., Whitesides G. M. (2001). Langmuir.

[cit6] McGorty R., Fung J., Kaz D., Manoharan V. (2010). Mater. Today.

[cit7] Zhang Z., Pfleiderer P., Schofield A. B., Clasen C., Vermant J. (2011). J. Am. Chem. Soc..

[cit8] Ershov D., Sprakel J., Appel J., Stuart M. A. C., van der Gucht J. (2013). Proc. Natl. Acad. Sci. U. S. A..

[cit9] Xie Q., Davies G., Harting J. (2017). ACS Nano.

[cit10] Goggin D. M., Samaniuk J. R. (2018). AIChE J..

[cit11] Goggin D. M., Zhang H., Miller E. M., Samaniuk J. R. (2020). ACS Nano.

[cit12] Loudet J. C., Alsayed A. M., Zhang J., Yodh A. G. (2005). Phys. Rev. Lett..

[cit13] Loudet J. C., Yodh A. G., Pouligny B. (2006). Phys. Rev. Lett..

[cit14] Lewandowski E. P., Bernate J. A., Searson P. C., Stebe K. J. (2008). Langmuir.

[cit15] Lewandowski E. P., Cavallaro Jr. M., Botto L., Bernate J. C., Garbin V., Stebe K. J. (2010). Langmuir.

[cit16] Nicolson M. M. (1949). Math. Proc. Cambridge Philos. Soc..

[cit17] Singh P., Joseph D. D. (2005). J. Fluid Mech..

[cit18] Vella D., Mahadevan L. (2005). Am. J. Phys..

[cit19] Würger A. (2006). Phys. Rev. E: Stat., Nonlinear, Soft Matter Phys..

[cit20] Léandri J., Würger A. (2013). J. Colloid Interface Sci..

[cit21] Blanc C., Fedorenko D., Gross M., In M., Abkarian M., Gharbi M. A., Fournier J. B., Galatola P., Nobili M. (2013). Phys. Rev. Lett..

[cit22] Galatola P., Fournier J. P. (2014). Soft Matter.

[cit23] Yao L., Sharifi-Mood N., Liu I. B., Stebe K. J. (2015). J. Colloid Interface Sci..

[cit24] Sharifi-Mood N., Liu I. B., Stebe K. J. (2015). Soft Matter.

[cit25] Galatola P. (2016). Soft Matter.

[cit26] Würger A. (2016). Soft Matter.

[cit27] Sharifi-Mood N., Liu I. B., Stebe K. J. (2016). Soft Matter.

[cit28] Galatola P. (2016). Soft Matter.

[cit29] Dominguez A., Oettel M., Dietrich S. (2008). J. Chem. Phys..

[cit30] Stamou D., Duschl C., Johannsmann D. (2000). Phys. Rev. E: Stat., Nonlinear, Soft Matter Phys..

[cit31] Danov K. D., Kralchevsky P. A., Naydenov B. N., Brenn G. (2005). J. Colloid Interface Sci..

[cit32] Pohl H. A. (1951). J. Appl. Phys..

[cit33] Pohl H. A. (1958). J. Appl. Phys..

[cit34] Pohl H. A., Crane J. S. (1972). J. Theor. Biol..

[cit35] Pohl H. A., Pollock K. (1978). J. Electrost..

[cit36] Pohl H. A., Pollock K., Crane J. S. (1978). J. Biol. Phys..

[cit37] Green N. G., Morgan H. (1999). J. Phys. Chem. B.

[cit38] Kang K. H., Kang Y., Xuan X., Li D. (2006). Electrophoresis.

[cit39] Jones T. B., Electromechanics of Particles, Cambridge University Press, 1995

[cit40] Liu H., Bau H. H. (2004). Phys. Fluids.

[cit41] Gifford W. A., Scriven L. E. (1971). Chem. Eng. Sci..

[cit42] Chan D. Y. C., Henry Jr. J. D., White L. R. (1981). J. Colloid Interface Sci..

[cit43] Hinsch K. (1983). J. Colloid Interface Sci..

[cit44] Camoin C., Roussell J. F., Faure R., Blanc R. (1987). Europhys. Lett..

[cit45] Kralchevsky P. A., Paunov V. N., Ivanov I. B., Nagayama K. (1992). J. Colloid Interface Sci..

[cit46] Kralchevsky P. A., Paunov V. N., Denkov N. D., Ivanov I. B., Nagayama K. (1993). J. Colloid Interface Sci..

[cit47] Paunov V. N., Kralchevsky P. A., Denkov N. D. (1993). J. Colloid Interface Sci..

[cit48] Davies G. B., Krüger T., Coveney P. V., Harting J., Bresme F. (2014). Soft Matter.

[cit49] Davies G. B., Krüger T., Coveney P. V., Harting J., Bresme F. (2014). Adv. Mater..

[cit50] Davies G. B., Krüger T., Coveney P. V., Harting J., Bresme F. (2014). Adv. Mater..

[cit51] Davies G. B., Botto L. (2015). Soft Matter.

[cit52] Botto L., Lewandowski E. P., Cavallaro M., Stebe K. J. (2012). Soft Matter.

[cit53] Basavaraj M. G., Fuller G. G., Fransaer J., Vermant J. (2006). Langmuir.

[cit54] Rothemund P. W. K. (2000). Proc. Natl. Acad. Sci. U. S. A..

[cit55] Kralchevsky P. A., Denkov N. D., Danov K. D. (2001). Langmuir.

[cit56] Liu I. B., Bigazzi G., Sharifi-Mood N., Yao L., Stebe K. J. (2017). Phys. Rev. Fluids.

[cit57] Read A., Kandy S. K., Liu I. B., Radhakrishnan R., Stebe K. J. (2020). Soft Matter.

[cit58] Liu I. B., Sharifi-Mood N., Stebe K. J. (2016). Philos. Trans. R. Soc., A.

[cit59] Hu D. L., Bush J. W. M. (2005). Nature.

[cit60] Cote L. J., Kim F., Huang J. (2009). J. Am. Chem. Soc..

[cit61] Imperiali L., Liao K. H., Clasen C., Fransaer J., Macosko C. W., Vermant J. (2012). Langmuir.

[cit62] Nikolaides M. G., Bausch A. R., Hsu M. F., Dinsmore A. D., Brenner M. P., Gay C., Weitz D. A. (2002). Nature.

[cit63] Megens M., Aizenberg J. (2003). Nature.

[cit64] Nikolaides M. G., Bausch A. R., Hsu M. F., Dinsmore A. D., Brenner M. P., Gay C., Weitz D. A. (2003). Nature.

[cit65] Foret L., Würger A. (2004). Phys. Rev. Lett..

[cit66] Danov K. D., Kralchevsky P. A., Boneva M. P. (2004). Langmuir.

[cit67] Würger A., Foret L. (2005). J. Phys. Chem. B.

[cit68] Oettel M., Domnguez A., Dietrich S. (2005). Phys. Rev. E: Stat., Nonlinear, Soft Matter Phys..

[cit69] Domnguez A., Oettel M., Dietrich S. (2005). J. Phys.: Condens. Matter.

[cit70] Oettel M., Domnguez A., Dietrich S. (2005). J. Phys.: Condens. Matter.

[cit71] Oettel M., Domnguez A., Dietrich S. (2006). Langmuir.

[cit72] Danov K. D., Kralchevsky P. A. (2006). Langmuir.

[cit73] Würger A. (2006). Europhys. Lett..

[cit74] Domnguez A., Oettel M., Dietrich S. (2007). Europhys. Lett..

[cit75] Würger A. (2007). Europhys. Lett..

[cit76] Singh P., Aubry N. (2005). Phys. Rev. E: Stat., Nonlinear, Soft Matter Phys..

[cit77] Jackson J. D., Classical Electrodynamics, John Wiley & Sons, 3rd edn, 1999

[cit78] Landau L. D. and Lifshitz E. M., Electrodynamics of Continuous Media, Pergamon Press, 1960

[cit79] Price R. H. (1992). Eur. J. Phys..

[cit80] Griffiths D. J., Introduction to Electrodynamics, Prentice Hall, 3rd edn, 1999

[cit81] Smoluchowski M. (1911). Bull. Int. Acad. Sci. Cracovie.

[cit82] Golusin G. M. (1934). Math. Sb..

[cit83] Happel J. and Brenner H., Low Reynolds Number Hydrodynamics, Springer, 1965

[cit84] Luke J. H. C. (1989). SIAM J. Appl. Math..

[cit85] Gascoyne P. R. C., Vykoukal J. (2002). Electrophoresis.

[cit86] Domnguez A., Oettel M., Dietrich S. (2007). J. Chem. Phys..

[cit87] Bonnecaze R. T., Brady J. F. (1998). J. Chem. Phys..

[cit88] de Gennes P. G., Brochard-Wyart D. and Quéré D., Capillarity and Wetting Phenomena: Drops, Bubbles, Pearls, Waves, Springer, 2004

[cit89] Newman J. and Thomas-Alyea K. E., Electrochemical Systems, John Wiley & Sons, 2004

[cit90] Bazant M. Z. (2016). Phys. Rev. Fluids.

[cit91] Landau L. D. and Lifshitz E. M., Fluid Mechanics, Pergamon Press, 1959

[cit92] Fournier J. B. (2007). Soft Matter.

[cit93] Galatola P. (2016). Phys. Rev. E: Stat., Nonlinear, Soft Matter Phys..

