# Supplementary material for: Capillary force on an ‘inert’ colloid: a physical analogy to dielectrophoresis
Source: Soft Matter. 2021 Feb 16;17(12):3417–42. doi: 10.1039/d0sm02143a (PMC8323820; doi:10.1039/d0sm02143a)
Supplement: SM-017-D0SM02143A-s001 [file SM-017-D0SM02143A-s001.pdf]

Electronic Supplementary Material (ESI) for Soft Matter.
This journal is © The Royal Society of Chemistry 2021

Supplemental Material for “Capillary force on an ‘inert’ colloid: a physical analogy to dielectrophoresis”

Joseph M. Barakat and Todd M. Squires

Contents

Introduction 5
S.1 Electric problem: method of reflections 5
S.1.1 Conducting particle 5
S.1.1.1 Governing equations 5
S.1.1.2 First reflection 7
S.1.1.3 Second reflection 8
S.1.1.4 Composite solution 9
S.1.1.5 Higher reflections: finite-size effects 10
S.1.2 Insulating particle 12
S.2 Electric problem: bipolar coordinates 13
S.2.1 Governing equations 13
S.2.2 Eigenfunction expansions 15
S.2.3 Solution of the series coefficients 15
S.2.4 Numerical procedure 16
S.3 Capillary problem: method of reflections 17
S.3.1 Particle with a pinned contact line 17
S.3.1.1 Governing equations 17
S.3.1.2 First reflection 19
S.3.1.3 Second reflection 20
S.3.1.4 Composite solution 21
S.3.1.5 Higher reflections: finite-size effects 22
S.3.2 Particle with an equilibrium contact angle 23
S.3.2.1 Cylindrical particle 24
S.3.2.2 Spherical particle 24
S.4 Capillary problem: bipolar coordinates 28
S.4.1 Governing equations 28
S.4.2 Eigenfunction expansions 29
S.4.3 Solution of the series coefficients 31
S.4.4 Numerical procedure 32
Appendix S.A Vector harmonics in two dimensions 34
Appendix S.B Transformation to bipolar coordinates 35
References 37

List of Tables

S.1 Analytical expressions for the electric force and energy, including the first finite-size correction, for a particle held in a fixed system of charges ($\mathcal{Q}$) or potentials ($\mathcal{V}$) [cf. Eqs. (S.1.38)-(S.1.45)]. 5

S.2 Analytical expressions for the capillary force and energy, including the first finite-size correction, for a particle held in a fixed system of heights ($\mathcal{U}$) or slopes ($\mathcal{P}$) [cf. Eqs. (S.3.41)-(S.3.48) and (S.3.54)-(S.3.55)]. 17

List of Figures

S.1 Electric force and energy for a conducting particle held in a fixed system of charges. The dimensionless force $\bar{\boldsymbol{F}} = \boldsymbol{F}/[\pi\varepsilon a^3|(\boldsymbol{\nabla E}^{\text{ext}})_{\boldsymbol{0}}|^2]$ (*top*) and energy $\bar{W} = W/[\pi\varepsilon a^4|(\boldsymbol{\nabla E}^{\text{ext}})_{\boldsymbol{0}}|^2]$ (*bottom*) are plotted against the relative particle position $\bar{\boldsymbol{\xi}} = (\boldsymbol{\xi} - \boldsymbol{\xi}^*)/a$ (*left*) and the radius ratio a/R (*right*), where $\boldsymbol{\xi}^*$ is the fixed point as defined by Eq. (3.24) in the main text. For the numerical calculations in bipolar coordinates, we set the field amplitudes $E_0 = E_1 = 1$ and phase angles $\alpha_0 = \alpha_1 = 0$ [cf. Eqs. (S.2.19)-(S.2.20)]. 11

S.2 Electric force and energy for a conducting particle held in a fixed system of potentials. The variables have the same meaning as in Fig. S.1. 12

S.3 Electric force and energy for an insulating particle held in a fixed system of charges. The variables have the same meaning as in Fig. S.1. 13

S.4 Electric force and energy for an insulating particle held in a fixed system of potentials. The variables have the same meaning as in Fig. S.1. 14

S.5 Capillary force and energy for a particle with a symmetrically pinned contact line trapped at an interface with a fixed height distribution on the outer shell. The dimensionless force $\bar{\boldsymbol{F}} = \boldsymbol{F}/[\frac{1}{4}\pi\gamma a^5|(\boldsymbol{\nabla K}^{\text{ext}})_{\boldsymbol{0}}|^2]$ (*top*) and energy $\bar{W} = W/[\frac{1}{4}\pi\gamma a^6|(\boldsymbol{\nabla K}^{\text{ext}})_{\boldsymbol{0}}|^2]$ (*bottom*) are plotted against the relative particle position $\bar{\boldsymbol{\xi}} = (\boldsymbol{\xi} - \boldsymbol{\xi}^*)/a$ (*left*) and the radius ratio a/R (*right*), where $\boldsymbol{\xi}^*$ is the fixed point as defined by Eq. (4.25) in the main text. For the numerical calculations in bipolar coordinates, we set the curvature amplitudes $K_0 = K_1 = 1$ and phase angles $\alpha_0 = \alpha_1 = 0$ [cf. Eqs. (S.4.37)-(S.4.38)]. 23

S.6 Capillary force and energy for a particle with a symmetrically pinned contact line trapped at an interface with a fixed outer slope distribution. The variables have the same meaning as in Fig. S.5. 24

S.7 Capillary force and energy for a cylindrical particle with an equilibrium contact angle of 90° trapped at an interface with a fixed outer height distribution. The variables have the same meaning as in Fig. S.5. 25

S.8 Capillary force and energy for a cylindrical particle with an equilibrium contact angle of 90° trapped at an interface with a fixed outer slope distribution. The variables have the same meaning as in Fig. S.5. 25

S.9 Capillary force and energy for a spherical particle with an equilibrium contact angle trapped at an interface with a fixed outer height distribution. The variables have the same meaning as in Fig. S.5. 27

S.10 Capillary force and energy for a spherical particle with an equilibrium contact angle trapped at an interface with a fixed outer slope distribution. The variables have the same meaning as in Fig. S.5. 27

S.11 Isocontours of the bipolar coordinates σ and τ. 36

Introduction

In this supplemental document, we provide details for the solution of the Laplace problems for the electric potential ψ and interface height ζ described in the main text (the relevant geometry is sketched in Fig. 3). For both the electric and capillary problems, we solve the Laplace problem approximately using the method of reflections[1–4] (§S.1 and §S.3) and validate our approximation using an exact solution in bipolar coordinates[3,5] (§S.2 and §S.4). Concurrently, we also calculate the (electric or capillary) force $\boldsymbol{F}$ and energy W. In the main text, results for $\boldsymbol{F}$ and W are presented in the "unbounded" limit as the shell radius $R \to \infty$. By expanding to higher reflections (and verifying against the bipolar solution), we also determine the first finite-size correction to $\boldsymbol{F}$ and W in terms of the ratio a/R, where a is the particle radius. The resulting analytical expressions are summarized in Tables S.1 and S.2 for the electric and capillary problems, respectively.

S.1 Electric problem: method of reflections

Table S.1 Analytical expressions for the electric force and energy, including the first finite-size correction, for a particle held in a fixed system of charges ($\mathcal{Q}$) or potentials ($\mathcal{V}$) [cf. Eqs. (S.1.38)-(S.1.45)].

Particle	Electric Force	Electric Energy
Conductor	$\boldsymbol{F}_{\mathcal{Q}} = \pi\varepsilon a^2\left(1-\dfrac{a^2}{R^2}\right)(\boldsymbol{\nabla}\lvert\boldsymbol{E}^{\text{ext}}\rvert^2)_{\boldsymbol{\xi}}+\cdots$	$W_{\mathcal{Q}} = -\pi\varepsilon a^2\left[\left(1-\dfrac{a^2}{R^2}\right)\lvert\boldsymbol{E}^{\text{ext}}_{\boldsymbol{\xi}}\rvert^2+\tfrac{1}{4}a^2\lvert(\boldsymbol{\nabla}\boldsymbol{E}^{\text{ext}})_{\boldsymbol{\xi}}\rvert^2\right]+\cdots$
	$\boldsymbol{F}_{\mathcal{V}} = \pi\varepsilon a^2\left(1+\dfrac{a^2}{R^2}\right)(\boldsymbol{\nabla}\lvert\boldsymbol{E}^{\text{ext}}\rvert^2)_{\boldsymbol{\xi}}+\cdots$	$W_{\mathcal{V}} = \pi\varepsilon a^2\left[\left(1+\dfrac{a^2}{R^2}\right)\lvert\boldsymbol{E}^{\text{ext}}_{\boldsymbol{\xi}}\rvert^2+\tfrac{1}{4}a^2\lvert(\boldsymbol{\nabla}\boldsymbol{E}^{\text{ext}})_{\boldsymbol{\xi}}\rvert^2\right]+\cdots$
Insulator	$\boldsymbol{F}_{\mathcal{Q}} = -\pi\varepsilon a^2\left(1+\dfrac{a^2}{R^2}\right)(\boldsymbol{\nabla}\lvert\boldsymbol{E}^{\text{ext}}\rvert^2)_{\boldsymbol{\xi}}+\cdots$	$W_{\mathcal{Q}} = \pi\varepsilon a^2\left[\left(1+\dfrac{a^2}{R^2}\right)\lvert\boldsymbol{E}^{\text{ext}}_{\boldsymbol{\xi}}\rvert^2+\tfrac{1}{4}a^2\lvert(\boldsymbol{\nabla}\boldsymbol{E}^{\text{ext}})_{\boldsymbol{\xi}}\rvert^2\right]+\cdots$
	$\boldsymbol{F}_{\mathcal{V}} = -\pi\varepsilon a^2\left(1-\dfrac{a^2}{R^2}\right)(\boldsymbol{\nabla}\lvert\boldsymbol{E}^{\text{ext}}\rvert^2)_{\boldsymbol{\xi}}+\cdots$	$W_{\mathcal{V}} = -\pi\varepsilon a^2\left[\left(1-\dfrac{a^2}{R^2}\right)\lvert\boldsymbol{E}^{\text{ext}}_{\boldsymbol{\xi}}\rvert^2+\tfrac{1}{4}a^2\lvert(\boldsymbol{\nabla}\boldsymbol{E}^{\text{ext}})_{\boldsymbol{\xi}}\rvert^2\right]+\cdots$

S.1.1 Conducting particle

Conducting particles are discussed in §3.1 in the main text. Below, we describe the mathematical details underlying the results presented in that section.

S.1.1.1 Governing equations

Laplace problem: Consider the boundary-value problem for a conducting particle described in the main text [Eqs. (3.8)-(3.11)]. The electric potential ψ obeys Laplace's equation,

$$\nabla^2\psi = 0 \quad \text{for} \quad r' \geq a \quad \text{and} \quad r \leq R, \tag{3.8}$$

subject to boundary conditions on the shell,

either $$\hat{\boldsymbol{n}}\cdot\boldsymbol{\nabla}\psi_{\mathcal{Q}} = \hat{\boldsymbol{n}}\cdot\boldsymbol{\nabla}\psi^{\text{ext}} \quad \text{at} \quad r = R \tag{3.9a}$$

or $$\psi_{\mathcal{V}} = \psi^{\text{ext}} \quad \text{at} \quad r = R, \tag{3.9b}$$

and the particle,

$$\psi = V \quad \text{at} \quad r' = a, \tag{3.10}$$

$$Q = -\varepsilon \oint_{r'=a} \hat{\boldsymbol{n}}\cdot\boldsymbol{\nabla}\psi \, \mathrm{d}s = 0. \tag{3.11}$$

Here, ψ^{ext} is the externally applied potential given by Eq. (3.1), V is the constant potential on the particle boundary, and Q is the net charge.

Electric potential: To solve this problem, we expand ψ and V as series of sequential approximations or "reflections,"

$$\psi = \sum_{n=0}^{\infty} \psi^{(n)}, \tag{S.1.2}$$

$$V = \sum_{n\ \text{odd}}^{\infty} V^{(n)}, \tag{S.1.3}$$

where the zeroth reflection is given by

$$\psi^{(0)} = \psi^{\text{ext}} = -\boldsymbol{E}_{\boldsymbol{0}}^{\text{ext}} \cdot \boldsymbol{r} - \tfrac{1}{2}(\boldsymbol{\nabla}\boldsymbol{E}^{\text{ext}})_{\boldsymbol{0}} : \boldsymbol{r}\boldsymbol{r} \tag{S.1.4}$$

[cf. Eq. (3.1)] and each subsequent reflection in the series is chosen to partially satisfy the boundary conditions (3.9)-(3.11) as follows:

$$\left.\begin{aligned} &\psi^{(n)} + \psi^{(n-1)} = V^{(n)} \\ &\oint_{r'=a} \hat{\boldsymbol{n}} \cdot \boldsymbol{\nabla}(\psi^{(n)} + \psi^{(n-1)})\,\mathrm{d}s = 0 \end{aligned}\right\} \quad \text{at} \quad r' = a, \qquad n = 1, 3, \ldots, \tag{S.1.5}$$

$$\left.\begin{aligned} &\hat{\boldsymbol{n}} \cdot \boldsymbol{\nabla}(\psi_Q^{(n)} + \psi_Q^{(n-1)}) = 0 \\ &\psi_{\mathcal{V}}^{(n)} + \psi_{\mathcal{V}}^{(n-1)} = 0 \end{aligned}\right\} \quad \text{at} \quad r = R, \qquad n = 2, 4, \ldots. \tag{S.1.6}$$

Thus, the even-numbered reflections $\psi^{(2)}, \psi^{(4)}, \ldots$ are determined by iteratively satisfying the outer boundary condition (3.9), which produces a series of growing harmonics emanating from the shell's center at $\boldsymbol{r} = \boldsymbol{0}$. The odd-numbered reflections $\psi^{(1)}$, $\psi^{(3)}, \ldots$ and $V^{(1)}, V^{(3)}, \ldots$ are chosen to satisfy the inner boundary conditions (3.10)-(3.11), resulting in a series of decaying harmonics emanating from the particle's center at $\boldsymbol{r} = \boldsymbol{\xi}$. Some useful properties of 2D vector harmonics are detailed in Appendix S.A of this supplemental document.

Electric force: Once the potential ψ is known, the force $\boldsymbol{F}$ may be calculated using Eq. (3.7) from the main text:

$$\boldsymbol{F} = \varepsilon \oint_{r'=a} \left[(\hat{\boldsymbol{n}} \cdot \boldsymbol{\nabla}\psi)\boldsymbol{\nabla}\psi - \tfrac{1}{2}|\boldsymbol{\nabla}\psi|^2 \hat{\boldsymbol{n}}\right] \mathrm{d}s. \tag{3.7}$$

Substituting Eq. (S.1.2) into (3.7) yields the double-series expansion,

$$\boldsymbol{F} \equiv \sum_{n=0}^{\infty}\sum_{m=0}^{n} \boldsymbol{F}^{(mn)} = \varepsilon \sum_{n=0}^{\infty}\sum_{m=0}^{\infty} \oint_{r'=a} \left[(\hat{\boldsymbol{n}} \cdot \boldsymbol{\nabla}\psi^{(m)})\boldsymbol{\nabla}\psi^{(n)} - \tfrac{1}{2}(\boldsymbol{\nabla}\psi^{(m)} \cdot \boldsymbol{\nabla}\psi^{(n)})\hat{\boldsymbol{n}}\right] \mathrm{d}s, \tag{S.1.7}$$

where $\boldsymbol{F}^{(mn)}$ depends upon the product of terms containing $\psi^{(m)}$ and $\psi^{(n)}$. In Eq. (S.1.7), we have defined $\boldsymbol{F}^{(mn)}$ such that m runs from 0 to n; thus, each nth reflection contributes $n + 1$ terms to the sum. E.g., for the zeroth reflection ($n = 0$), we need only compute one term:

$$\begin{aligned} \boldsymbol{F}^{(00)} &= \varepsilon \oint_{r'=a} \hat{\boldsymbol{n}} \cdot \left(\boldsymbol{\nabla}\psi^{(0)}\boldsymbol{\nabla}\psi^{(0)} - \tfrac{1}{2}|\boldsymbol{\nabla}\psi^{(0)}|^2 \boldsymbol{\delta}\right) \mathrm{d}s \\ &= \boldsymbol{0}. \end{aligned} \tag{S.1.8}$$

Thus, the self-coupling of the zeroth reflection $\psi^{(0)}$ (i.e., the external potential) contributes no force to the particle.

Electric energy: To calculate the insertion energy W, we must apply Eq. (3.5) from the main text:

$$W = \tfrac{1}{2}\varepsilon \iint_{\substack{r' \geq a \\ r \leq R}} |\boldsymbol{\nabla}\psi|^2 \,\mathrm{d}^2\boldsymbol{r} - \tfrac{1}{2}\varepsilon \iint_{r \leq R} |\boldsymbol{\nabla}\psi^{\text{ext}}|^2 \,\mathrm{d}^2\boldsymbol{r}. \tag{3.5}$$

After integrating by parts and using $\nabla^2\psi = \nabla^2\psi^{\text{ext}} = 0$ to eliminate the area integrals, we obtain

$$W = -\tfrac{1}{2}\varepsilon \oint_{r'=a} \psi(\hat{\boldsymbol{n}} \cdot \boldsymbol{\nabla}\psi)\mathrm{d}s - \tfrac{1}{2}\varepsilon \oint_{r=R} \hat{\boldsymbol{n}} \cdot (\psi\boldsymbol{\nabla}\psi - \psi^{\text{ext}}\boldsymbol{\nabla}\psi^{\text{ext}})\,\mathrm{d}s, \tag{S.1.9}$$

which depends only upon line integrals over the particle and shell boundaries. Substituting Eq. (S.1.2) into (S.1.9) for ψ and setting $\psi^{\text{ext}} = \psi^{(0)}$ yields the double-series expansion,

$$W \equiv \sum_{n=0}^{\infty}\sum_{m=0}^{n} W^{(mn)} = -\tfrac{1}{2}\varepsilon \sum_{n=0}^{\infty}\sum_{m=0}^{\infty} \left(\oint_{r'=a} \psi^{(m)}(\hat{\boldsymbol{n}} \cdot \boldsymbol{\nabla}\psi^{(n)})\mathrm{d}s + \oint_{r=R} \hat{\boldsymbol{n}} \cdot (\psi^{(m)}\boldsymbol{\nabla}\psi^{(n)} - \psi^{(0)}\boldsymbol{\nabla}\psi^{(0)})\,\mathrm{d}s\right), \tag{S.1.10}$$

where $W^{(mn)}$ depends upon the product of terms containing $\psi^{(m)}$ and $\psi^{(n)}$. Using Eq. (S.1.4), we may calculate the part of the energy due solely to $\psi^{(0)}$:

$$\begin{aligned} W^{(00)} &= -\tfrac{1}{2}\varepsilon \oint_{r'=a} \psi^{(0)}(\hat{\boldsymbol{n}} \cdot \boldsymbol{\nabla}\psi^{(0)})\,\mathrm{d}s \\ &= -\tfrac{1}{2}\pi\varepsilon a^2 \left[|\boldsymbol{E}^{\text{ext}}_{\boldsymbol{\xi}}|^2 + \tfrac{1}{4}a^2|(\boldsymbol{\nabla}\boldsymbol{E}^{\text{ext}})_{\boldsymbol{\xi}}|^2\right], \end{aligned} \tag{S.1.11}$$

i.e., the work done to eliminate the external field within the particle. Equation (S.1.11) does not account for the field disturbance created by the particle, which contributes like-ordered terms to the energy. These terms depend on higher reflections.

Below, we compute the sequential approximations $\psi^{(n)}$, $V^{(n)}$, $\boldsymbol{F}^{(mn)}$, and $W^{(mn)}$ for the first few values of n and $m = 0, \dots, n$. We implicitly assume that the particle is much smaller than the characteristic dimension of the shell, $a/R \ll 1$, and sufficiently far away from the shell boundary, $\xi = o(R)$. Initially, we shall approximate the reflections such that only the leading-order contributions to the force and energy are retained, neglecting errors of $O(a^2/R^2)$. These leading-order contributions are exact in the limit as the shell becomes infinitely large. Subsequently, we evaluate the $O(a^2/R^2)$ correction to the force and energy, which accounts for the finite size of the shell.

S.1.1.2 First reflection

The particle-free potential $\psi^{(0)}$ violates the particle boundary conditions (3.10)-(3.11). Thus, we add the first reflection $\psi^{(1)}$ such that

$$\nabla^2\psi^{(1)} = 0, \tag{S.1.12}$$

$$\psi^{(1)} + \psi^{(0)} = V^{(1)} \quad \text{at} \quad r' = a, \tag{S.1.13}$$

$$\oint_{r'=a} \hat{\boldsymbol{n}} \cdot \boldsymbol{\nabla}(\psi^{(1)} + \psi^{(0)})\,\mathrm{d}s = 0, \tag{S.1.14}$$

which remediates the conditions at $r' = a$. To solve the boundary-value problem (S.1.12)-(S.1.14), it is convenient to first recast $\psi^{(0)}$ in terms of the particle-centered position $\boldsymbol{r}'$. Substituting $\boldsymbol{r} = \boldsymbol{r}' + \boldsymbol{\xi}$ into Eq. (S.1.4) gives,

$$\psi^{(0)} = -\boldsymbol{E}^{\text{ext}}_{\boldsymbol{0}} \cdot \boldsymbol{\xi} - \tfrac{1}{2}(\boldsymbol{\nabla}\boldsymbol{E}^{\text{ext}})_{\boldsymbol{0}} : \boldsymbol{\xi}\boldsymbol{\xi} - \boldsymbol{E}^{\text{ext}}_{\boldsymbol{\xi}} \cdot \boldsymbol{r}' - \tfrac{1}{2}(\boldsymbol{\nabla}\boldsymbol{E}^{\text{ext}})_{\boldsymbol{\xi}} : \boldsymbol{r}'\boldsymbol{r}', \tag{S.1.15}$$

where we have defined the external field and field gradient at the particle's center:

$$\boldsymbol{E}^{\text{ext}}_{\boldsymbol{\xi}} = \boldsymbol{E}^{\text{ext}}_{\boldsymbol{0}} + (\boldsymbol{\nabla}\boldsymbol{E}^{\text{ext}})_{\boldsymbol{0}} \cdot \boldsymbol{\xi}, \tag{S.1.16}$$

$$(\boldsymbol{\nabla}\boldsymbol{E}^{\text{ext}})_{\boldsymbol{\xi}} = (\boldsymbol{\nabla}\boldsymbol{E}^{\text{ext}})_{\boldsymbol{0}}. \tag{S.1.17}$$

Substituting Eq. (S.1.15) into (S.1.13)-(S.1.14) and integrating then yields the first reflection

$$\psi^{(1)} = a^2\boldsymbol{E}^{\text{ext}}_{\boldsymbol{\xi}} \cdot \frac{\boldsymbol{r}'}{r'^2} + \tfrac{1}{2}a^4(\boldsymbol{\nabla}\boldsymbol{E}^{\text{ext}})_{\boldsymbol{\xi}} : \frac{\boldsymbol{r}'\boldsymbol{r}'}{r'^4}, \tag{S.1.18}$$

$$V^{(1)} = -\boldsymbol{E}^{\text{ext}}_{\boldsymbol{0}} \cdot \boldsymbol{\xi} - \tfrac{1}{2}(\boldsymbol{\nabla}\boldsymbol{E}^{\text{ext}})_{\boldsymbol{0}} : \boldsymbol{\xi}\boldsymbol{\xi}. \tag{S.1.19}$$

It is straightforward to verify that Eqs. (S.1.18)-(S.1.19) uniquely satisfy (S.1.12)-(S.1.14). Thus, the first reflection induces dipolar and quadrupolar disturbances to the potential. The sum $\psi^{(0)} + \psi^{(1)}$ represents the potential in a semi-infinite dielectric medium, which is exact in the limit as the shell radius $R \to \infty$.

At this order of approximation, we may compute the following contributions to the force from Eq. (S.1.7):

$$\begin{aligned} \boldsymbol{F}^{(01)} &= \varepsilon \oint_{r'=a} \hat{\boldsymbol{n}} \cdot \left[\boldsymbol{\nabla}\psi^{(0)}\boldsymbol{\nabla}\psi^{(1)} + \boldsymbol{\nabla}\psi^{(1)}\boldsymbol{\nabla}\psi^{(0)} - (\boldsymbol{\nabla}\psi^{(0)} \cdot \boldsymbol{\nabla}\psi^{(1)})\boldsymbol{\delta}\right] \mathrm{d}s \\ &= 2\pi\varepsilon a^2 \boldsymbol{E}^{\text{ext}}_{\boldsymbol{\xi}} \cdot (\boldsymbol{\nabla}\boldsymbol{E}^{\text{ext}})_{\boldsymbol{\xi}}, \end{aligned} \tag{S.1.20}$$

$$\begin{aligned} \boldsymbol{F}^{(11)} &= \varepsilon \oint_{r'=a} \hat{\boldsymbol{n}} \cdot \left(\boldsymbol{\nabla}\psi^{(1)}\boldsymbol{\nabla}\psi^{(1)} - \tfrac{1}{2}|\boldsymbol{\nabla}\psi^{(1)}|^2\boldsymbol{\delta}\right) \mathrm{d}s \\ &= \boldsymbol{0}. \end{aligned} \tag{S.1.21}$$

Thus, the first non-vanishing contribution to the force arises from the coupling between the particle-free potential $\psi^{(0)}$ and the leading-order ("unbounded") disturbance $\psi^{(1)}$. Using Eq. (S.1.10), we obtain the following contributions to the energy:

$$W^{(01)} = -\varepsilon \oint_{r'=a} \psi^{(0)}(\hat{\boldsymbol{n}} \cdot \boldsymbol{\nabla}\psi^{(1)})\mathrm{d}s - \varepsilon \oint_{r=R} \psi^{(0)}(\hat{\boldsymbol{n}} \cdot \boldsymbol{\nabla}\psi^{(1)})\,\mathrm{d}s$$
$$= 0, \tag{S.1.22}$$
$$W^{(11)} = -\tfrac{1}{2}\varepsilon \oint_{r'=a} \psi^{(1)}(\hat{\boldsymbol{n}} \cdot \boldsymbol{\nabla}\psi^{(1)})\mathrm{d}s - \tfrac{1}{2}\varepsilon \oint_{r=R} \psi^{(1)}(\hat{\boldsymbol{n}} \cdot \boldsymbol{\nabla}\psi^{(1)})\,\mathrm{d}s$$
$$\simeq \tfrac{1}{2}\pi\varepsilon a^2 \left[|\boldsymbol{E}^{\mathrm{ext}}_{\boldsymbol{\xi}}|^2 + \tfrac{1}{4}a^2|(\boldsymbol{\nabla}\boldsymbol{E}^{\mathrm{ext}})_{\boldsymbol{\xi}}|^2\right], \tag{S.1.23}$$

where terms of $O(a^2/R^2)$ been neglected in Eq. (S.1.23). The integrals over $r = R$ in Eqs. (S.1.22)-(S.1.23) are evaluated by first expanding the decaying harmonics with respect to $\boldsymbol{r}'$ (contained in $\psi^{(1)}$) as an infinite series of harmonics in $\boldsymbol{r}$, using Eq. (S.A.4) in Appendix S.A. Then, each term in the series is integrated over $r = R$ up to the desired level of accuracy. It should be remembered that the unit normal $\hat{\boldsymbol{n}} = \boldsymbol{r}'/r'$ and $-\boldsymbol{r}/r$ at $r' = a$ and $r = R$, respectively.

The derived expression for $W^{(11)}$ [Eq. (S.1.23)] exactly cancels $W^{(00)}$ [Eq. (S.1.11)] in the limit as $R \to \infty$; that is, $W^{(00)} + W^{(11)} \simeq 0$. This implies that truncating the approximation for the potential after the first reflection is insufficient to calculate the energy. The source of error can be traced to the fact that $\psi^{(0)} + \psi^{(1)}$ does not satisfy the electrostatic condition at $r = R$. As we show in the next section, adding the second reflection $\psi^{(2)}$ results in a finite energy.

S.1.1.3 Second reflection

The addition of the first reflection $\psi^{(1)}$ perturbs the shell boundary condition (3.9) at $r = R$. Thus, we add a correction $\psi^{(2)}$ that satisfies Laplace's equation,

$$\nabla^2\psi^{(2)} = 0, \tag{S.1.24}$$

subject to one of two conditions on the outer shell:

either $$\hat{\boldsymbol{n}} \cdot \boldsymbol{\nabla}(\psi^{(2)}_{Q} + \psi^{(1)}) = 0 \quad \text{at} \quad r = R \tag{S.1.25a}$$

or $$\psi_{\mathcal{V}} + \psi^{(1)} = 0 \quad \text{at} \quad r = R. \tag{S.1.25b}$$

To solve this problem, we must first rewrite $\psi^{(1)}$ in terms of the shell-centered position $\boldsymbol{r}$. Substituting $\boldsymbol{r}' = \boldsymbol{r} - \boldsymbol{\xi}$ into Eq. (S.1.18) and Taylor expanding about $\xi/r = 0$ [cf. Eq. (S.A.4) in Appendix S.A] gives

$$\psi^{(1)} = a^2\boldsymbol{E}^{\mathrm{ext}}_{\boldsymbol{\xi}} \cdot \frac{\boldsymbol{r} - \boldsymbol{\xi}}{|\boldsymbol{r} - \boldsymbol{\xi}|^2} + \tfrac{1}{2}a^4(\boldsymbol{\nabla}\boldsymbol{E}^{\mathrm{ext}})_{\boldsymbol{\xi}} : \frac{(\boldsymbol{r} - \boldsymbol{\xi})(\boldsymbol{r} - \boldsymbol{\xi})}{|\boldsymbol{r} - \boldsymbol{\xi}|^4}$$
$$= a^2\boldsymbol{E}^{\mathrm{ext}}_{\boldsymbol{\xi}} \cdot \sum_{n=0}^{\infty} \frac{(-\boldsymbol{\xi} \cdot \boldsymbol{\nabla})^n}{n!}\left(\frac{\boldsymbol{r}}{r^2}\right) + \tfrac{1}{2}a^4(\boldsymbol{\nabla}\boldsymbol{E}^{\mathrm{ext}})_{\boldsymbol{\xi}} : \sum_{n=0}^{\infty} \frac{(-\boldsymbol{\xi} \cdot \boldsymbol{\nabla})^n}{n!}\left(\frac{\boldsymbol{r}\boldsymbol{r}}{r^4}\right). \tag{S.1.26}$$

Applying Eqs. (S.A.5)-(S.A.6) in Appendix S.A, we may show that the solutions satisfying the fixed-charge [Eq. (S.1.25a)] and fixed-potential [Eq. (S.1.25b)] conditions are equal in magnitude but opposite in sign: $\psi^{(2)}_{Q} = -\psi^{(2)}_{\mathcal{V}}$. Substituting Eq. (S.1.26) for $\psi^{(1)}$ and integrating, we arrive at the expression,

$$\psi^{(2)}_{Q} = -\psi^{(2)}_{\mathcal{V}} = a^2\boldsymbol{E}^{\mathrm{ext}}_{\boldsymbol{\xi}} \cdot \sum_{n=0}^{\infty}\left(\frac{r}{R}\right)^{2(n+1)} \frac{(-\boldsymbol{\xi} \cdot \boldsymbol{\nabla})^n}{n!}\left(\frac{\boldsymbol{r}}{r^2}\right) + \tfrac{1}{2}a^4(\boldsymbol{\nabla}\boldsymbol{E}^{\mathrm{ext}})_{\boldsymbol{\xi}} : \sum_{n=0}^{\infty}\left(\frac{r}{R}\right)^{2(n+2)} \frac{(-\boldsymbol{\xi} \cdot \boldsymbol{\nabla})^n}{n!}\left(\frac{\boldsymbol{r}\boldsymbol{r}}{r^4}\right)$$
$$= a^2\boldsymbol{E}^{\mathrm{ext}}_{\boldsymbol{\xi}} \cdot \left(\frac{\boldsymbol{r}}{R^2} + \frac{2\boldsymbol{r}(\boldsymbol{r} \cdot \boldsymbol{\xi}) - r^2\boldsymbol{\xi}}{R^4} + \cdots\right) + \tfrac{1}{2}a^4(\boldsymbol{\nabla}\boldsymbol{E}^{\mathrm{ext}})_{\boldsymbol{\xi}} : \left(\frac{\boldsymbol{r}\boldsymbol{r}}{R^4} + \cdots\right). \tag{S.1.27}$$

Thus, the second reflection induces an infinite series of growing harmonics in order to rectify the boundary condition at $r = R$. For the purpose of calculating the leading-order contributions to the force and energy, it is sufficient to retain only the terms shown in Eq. (S.1.27). These terms satisfy the electrostatic condition at $r = R$ up to $O(a^3/R^3)$ errors. The remaining terms in the series may be omitted, as these contribute corrections of similar magnitude to the higher reflections.

It can be verified that the second reflection contributes a negligible correction to the force, up to the desired order of accuracy:

$$\begin{aligned}
\boldsymbol{F}^{(02)} &= \varepsilon \oint\limits_{r'=a} \hat{\boldsymbol{n}} \cdot \left[\boldsymbol{\nabla}\psi^{(0)}\boldsymbol{\nabla}\psi^{(2)} + \boldsymbol{\nabla}\psi^{(2)}\boldsymbol{\nabla}\psi^{(0)} - (\boldsymbol{\nabla}\psi^{(0)} \cdot \boldsymbol{\nabla}\psi^{(2)})\boldsymbol{\delta}\right] \mathrm{d}s \\
&= \mathbf{0}, \qquad \text{(S.1.28)} \\
\boldsymbol{F}^{(12)} &= \varepsilon \oint\limits_{r'=a} \hat{\boldsymbol{n}} \cdot \left[\boldsymbol{\nabla}\psi^{(1)}\boldsymbol{\nabla}\psi^{(2)} + \boldsymbol{\nabla}\psi^{(2)}\boldsymbol{\nabla}\psi^{(1)} - (\boldsymbol{\nabla}\psi^{(1)} \cdot \boldsymbol{\nabla}\psi^{(2)})\boldsymbol{\delta}\right] \mathrm{d}s \\
&\simeq \mathbf{0}, \qquad \text{(S.1.29)} \\
\boldsymbol{F}^{(22)} &= \varepsilon \oint\limits_{r'=a} \hat{\boldsymbol{n}} \cdot \left(\boldsymbol{\nabla}\psi^{(2)}\boldsymbol{\nabla}\psi^{(2)} - \tfrac{1}{2}|\boldsymbol{\nabla}\psi^{(2)}|^2\boldsymbol{\delta}\right) \mathrm{d}s \\
&= \mathbf{0}. \qquad \text{(S.1.30)}
\end{aligned}$$

Note that Eqs. (S.1.28) and (S.1.30) are exact, whereas (S.1.29) is an approximation for small a/R – terms of $O(a^4/R^4)$ have been neglected in Eq. (S.1.29).

The second reflection also induces a series of additional corrections $W^{(02)}$, $W^{(12)}$, and $W^{(22)}$ to the energy. Of these, the dominant contribution is due to $W^{(02)}$ and may be readily calculated by use of Eqs. (S.1.4) and (S.1.27):

$$\begin{aligned}
W_Q^{(02)} &= -\varepsilon \oint\limits_{r'=a} \psi^{(0)}(\hat{\boldsymbol{n}} \cdot \boldsymbol{\nabla}\psi_Q^{(2)})\mathrm{d}s - \varepsilon \oint\limits_{r=R} \psi^{(0)}(\hat{\boldsymbol{n}} \cdot \boldsymbol{\nabla}\psi_Q^{(2)})\,\mathrm{d}s \\
&\simeq -\pi\varepsilon a^2 \left[|\boldsymbol{E}_{\boldsymbol{\xi}}^{\mathrm{ext}}|^2 + \tfrac{1}{4}a^2|(\boldsymbol{\nabla}\boldsymbol{E}^{\mathrm{ext}})_{\boldsymbol{\xi}}|^2\right], \qquad \text{(S.1.31a)}
\end{aligned}$$

for the fixed-charge case, and

$$\begin{aligned}
W_{\mathcal{V}}^{(02)} &= -\varepsilon \oint\limits_{r'=a} \psi^{(0)}(\hat{\boldsymbol{n}} \cdot \boldsymbol{\nabla}\psi_{\mathcal{V}}^{(2)})\mathrm{d}s - \varepsilon \oint\limits_{r=R} \psi^{(0)}(\hat{\boldsymbol{n}} \cdot \boldsymbol{\nabla}\psi_{\mathcal{V}}^{(2)})\,\mathrm{d}s \\
&\simeq \pi\varepsilon a^2 \left[|\boldsymbol{E}_{\boldsymbol{\xi}}^{\mathrm{ext}}|^2 + \tfrac{1}{4}a^2|(\boldsymbol{\nabla}\boldsymbol{E}^{\mathrm{ext}})_{\boldsymbol{\xi}}|^2\right], \qquad \text{(S.1.31b)}
\end{aligned}$$

for the fixed-potential case [again, terms of $O(a^2/R^2)$ have been neglected]. Equations (S.1.31) give a nontrivial result because the line integral $\oint_{r=R} \psi^{(0)}(\hat{\boldsymbol{n}} \cdot \boldsymbol{\nabla}\psi^{(2)})\,\mathrm{d}s$ is finite, regardless of the value of a/R. Even though $\psi^{(2)}$ is *locally* smaller than $\psi^{(0)}$ by a factor of R^2/a^2, the coupling between $\psi^{(0)}$ and $\psi^{(2)}$ over an $O(R^2)$ region produces a non-negligible and *non-local* integral contribution to the total energy. *Neglecting this contribution altogether would lead one to conclude, incorrectly, that no energy is required to insert a conducting particle into a dielectric medium.* The other contributions to the energy are negligible:

$$\begin{aligned}
W^{(12)} &= -\tfrac{1}{2}\varepsilon \oint\limits_{r'=a} \left[\psi^{(1)}(\hat{\boldsymbol{n}} \cdot \boldsymbol{\nabla}\psi^{(2)}) + \psi^{(2)}(\hat{\boldsymbol{n}} \cdot \boldsymbol{\nabla}\psi^{(1)})\right] \mathrm{d}s - \tfrac{1}{2}\varepsilon \oint\limits_{r=R} \left[\psi^{(1)}(\hat{\boldsymbol{n}} \cdot \boldsymbol{\nabla}\psi^{(2)}) + \psi^{(2)}(\hat{\boldsymbol{n}} \cdot \boldsymbol{\nabla}\psi^{(1)})\right] \mathrm{d}s \\
&= 0, \qquad \text{(S.1.32)} \\
W^{(22)} &= -\tfrac{1}{2}\varepsilon \oint\limits_{r'=a} \psi^{(2)}(\hat{\boldsymbol{n}} \cdot \boldsymbol{\nabla}\psi^{(2)})\mathrm{d}s - \tfrac{1}{2}\varepsilon \oint\limits_{r=R} \psi^{(2)}(\hat{\boldsymbol{n}} \cdot \boldsymbol{\nabla}\psi^{(2)})\,\mathrm{d}s \\
&\simeq 0. \qquad \text{(S.1.33)}
\end{aligned}$$

Here, Eq. (S.1.32) is exact while (S.1.33) neglects errors of $O(a^2/R^2)$.

S.1.1.4 Composite solution

Up to the second reflection, the solution for the potential is obtained by summing Eqs. (S.1.4), (S.1.18), and (S.1.27). The result, for the case of fixed charges on the outer shell, is given by

$$\psi_Q = \underbrace{-\boldsymbol{E}_{\mathbf{0}}^{\mathrm{ext}} \cdot \boldsymbol{r} - \tfrac{1}{2}(\boldsymbol{\nabla}\boldsymbol{E}^{\mathrm{ext}})_{\mathbf{0}} : \boldsymbol{r}\boldsymbol{r}}_{\psi^{(0)}} + \underbrace{a^2\boldsymbol{E}_{\boldsymbol{\xi}}^{\mathrm{ext}} \cdot \frac{\boldsymbol{r}'}{r'^2} + \tfrac{1}{2}a^4(\boldsymbol{\nabla}\boldsymbol{E}^{\mathrm{ext}})_{\boldsymbol{\xi}} : \frac{\boldsymbol{r}'\boldsymbol{r}'}{r'^4}}_{\psi^{(1)}}$$

$$+\underbrace{a^2\boldsymbol{E}^{\text{ext}}_{\boldsymbol{\xi}}\cdot\left(\frac{\boldsymbol{r}}{R^2}+\frac{2\boldsymbol{r}(\boldsymbol{r}\cdot\boldsymbol{\xi})-r^2\boldsymbol{\xi}}{R^4}+\cdots\right)+\tfrac{1}{2}a^4(\boldsymbol{\nabla}\boldsymbol{E}^{\text{ext}})_{\boldsymbol{\xi}}:\left(\frac{\boldsymbol{rr}}{R^4}+\cdots\right)}_{\psi_{Q}^{(2)}}+\cdots,\tag{S.1.34}$$

which, after recombining terms, gives Eq. (3.13) in the main text. Similarly, the solution for fixed potentials on the outer shell is

$$\begin{aligned}\psi_{\mathcal{V}}=&\underbrace{-\boldsymbol{E}^{\text{ext}}_{\boldsymbol{0}}\cdot\boldsymbol{r}-\tfrac{1}{2}(\boldsymbol{\nabla}\boldsymbol{E}^{\text{ext}})_{\boldsymbol{0}}:\boldsymbol{rr}}_{\psi^{(0)}}+\underbrace{a^2\boldsymbol{E}^{\text{ext}}_{\boldsymbol{\xi}}\cdot\frac{\boldsymbol{r}'}{r'^2}+\tfrac{1}{2}a^4(\boldsymbol{\nabla}\boldsymbol{E}^{\text{ext}})_{\boldsymbol{\xi}}:\frac{\boldsymbol{r}'\boldsymbol{r}'}{r'^4}}_{\psi^{(1)}}\\&\underbrace{-a^2\boldsymbol{E}^{\text{ext}}_{\boldsymbol{\xi}}\cdot\left(\frac{\boldsymbol{r}}{R^2}+\frac{2\boldsymbol{r}(\boldsymbol{r}\cdot\boldsymbol{\xi})-r^2\boldsymbol{\xi}}{R^4}+\cdots\right)-\tfrac{1}{2}a^4(\boldsymbol{\nabla}\boldsymbol{E}^{\text{ext}})_{\boldsymbol{\xi}}:\left(\frac{\boldsymbol{rr}}{R^4}+\cdots\right)}_{\psi_{\mathcal{V}}^{(2)}}+\cdots,\end{aligned}\tag{S.1.35}$$

which differs from (S.1.34) because $\psi_{\mathcal{V}}^{(2)}=-\psi_{Q}^{(2)}$. After recombining terms in Eq. (S.1.35), we obtain (3.14) from the main text. A sketch of each reflection is shown in Fig. 4.

After summing Eqs. (S.1.8), (S.1.20)-(S.1.21), and (S.1.28)-(S.1.30), we obtain the following solution for the force:

$$\boldsymbol{F}=\underbrace{\pi\varepsilon a^2(\boldsymbol{\nabla}|\boldsymbol{E}^{\text{ext}}|^2)_{\boldsymbol{\xi}}}_{\boldsymbol{F}^{(01)}}+\cdots,\tag{S.1.36}$$

where we have used $2\boldsymbol{E}^{\text{ext}}_{\boldsymbol{\xi}}\cdot(\boldsymbol{\nabla}\boldsymbol{E}^{\text{ext}})_{\boldsymbol{\xi}}=(\boldsymbol{\nabla}|\boldsymbol{E}^{\text{ext}}|^2)_{\boldsymbol{\xi}}$. Equation (S.1.36) is equivalent to (3.15) in the main text. The dominant force contribution $\boldsymbol{F}^{(01)}$ is attributed to the coupling between $\psi^{(0)}$ and $\psi^{(1)}$; thus, it is independent of the condition at $r=R$ that establishes the field. In the limit as $R\to\infty$, Eq. (S.1.36) is exact. Higher-order corrections, which are omitted in Eq. (S.1.36), are of $O(a^2/R^2)$ and reflect the finite size of the shell. These corrections are *not* independent of the electrostatic condition at $r=R$, and are discussed further in §S.1.1.5.

Summing Eqs. (S.1.11), (S.1.22)-(S.1.23), and (S.1.31)-(S.1.33) yields the following energy:

$$W_Q=-W_{\mathcal{V}}=\underbrace{-\pi\varepsilon a^2\left[|\boldsymbol{E}^{\text{ext}}_{\boldsymbol{\xi}}|^2+\tfrac{1}{4}a^2|(\boldsymbol{\nabla}\boldsymbol{E}^{\text{ext}})_{\boldsymbol{\xi}}|^2\right]}_{W_Q^{(02)}\text{ or }-W_{\mathcal{V}}^{(02)}}+\cdots.\tag{S.1.37}$$

This expression is equivalent to Eqs. (3.17) and (3.18) in the main text. Since $W^{(00)}$ and $W^{(11)}$ exactly cancel each other in the limit as $R\to\infty$, the only finite contribution is due to $W^{(02)}$. This term results from the coupling between $\psi^{(0)}$ and $\psi^{(2)}$. Finite-size corrections are of $O(a^2/R^2)$ and are discussed in §S.1.1.5.

S.1.1.5 Higher reflections: finite-size effects

The results (S.1.36) and (S.1.37) presented above are exact if the shell radius R is infinitely large compared to the particle radius a. In this limit, one need only compute the first reflection $\psi^{(1)}$ emitted from the particle to calculate the force $\boldsymbol{F}$ from Eq. (S.1.7); to compute the energy W from Eq. (S.1.10), one additionally needs the second reflection $\psi^{(2)}$ emitted from the outer shell. For infinitely large domains, the force is independent of the conditions used to establish the field, whereas the energy is not.

The situation changes if the shell radius R is comparable to the particle radius a. For finite-sized shells, the force must depend on the condition used to establish the external field. The reason is the local field in the vicinity of the particle is now influenced by reflected modes from the outer shell. Higher odd- and even-numbered reflections may be computed by retracing the steps described in §S.1.1.2 and §S.1.1.3, respectively. The third reflection $\psi^{(3)}$ contributes an $O(a^2/R^2)$ correction to the force $\boldsymbol{F}$ by coupling to the ambient potential $\psi^{(0)}$, adding a term $\boldsymbol{F}^{(03)}$ to Eq. (S.1.7). This correction reverses sign depending upon whether the charges or potentials are held fixed on the outer shell. For the energy W, it is necessary to determine the potential up to the fourth reflection $\psi^{(4)}$; the coupling terms $W^{(11)}$, $W^{(02)}$, $W^{(22)}$, $W^{(13)}$, and $W^{(04)}$ all give $O(a^2/R^2)$ contributions to Eq. (S.1.10). Subsequent reflections contribute corrections to $\boldsymbol{F}$ and W in successive powers of a^2/R^2.

Calculating the third and fourth reflections (not presented here, for the sake of brevity) allows us to expand Eqs. (S.1.36) and (S.1.37) up to the $O(a^2/R^2)$ correction. Note that we must also expand the lower-order reflections up to $O(a^2/R^2)$ terms,

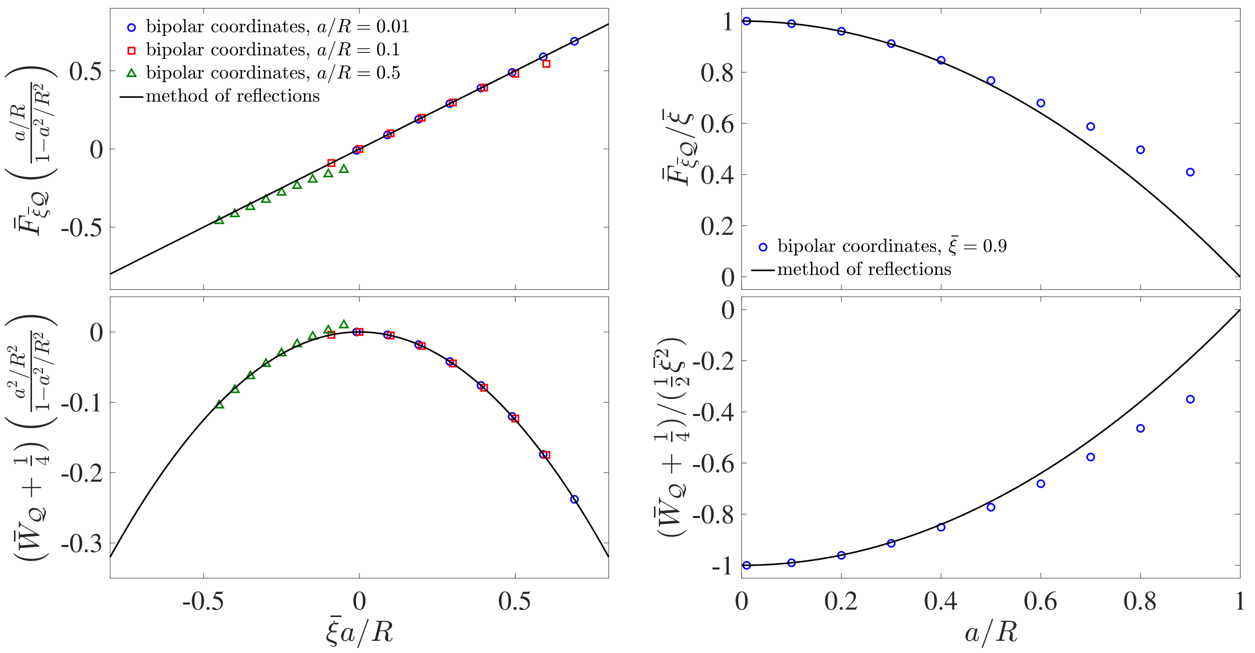

Figure S.1 Electric force and energy for a conducting particle held in a fixed system of charges. The dimensionless force $\bar{\boldsymbol{F}} = \boldsymbol{F}/[\pi\varepsilon a^3|(\boldsymbol{\nabla E}^{\text{ext}})_{\mathbf{0}}|^2]$ (*top*) and energy $\bar{W} = W/[\pi\varepsilon a^4|(\boldsymbol{\nabla E}^{\text{ext}})_{\mathbf{0}}|^2]$ (*bottom*) are plotted against the relative particle position $\bar{\boldsymbol{\xi}} = (\boldsymbol{\xi} - \boldsymbol{\xi}^*)/a$ (*left*) and the radius ratio a/R (*right*), where $\boldsymbol{\xi}^*$ is the fixed point as defined by Eq. (3.24) in the main text. For the numerical calculations in bipolar coordinates, we set the field amplitudes $E_0 = E_1 = 1$ and phase angles $\alpha_0 = \alpha_1 = 0$ [cf. Eqs. (S.2.19)-(S.2.20)].

whereas we had previously invoked the limit $R \to \infty$. For finite-sized shells with a fixed charge distribution, we obtain the following approximations for the force and energy:

$$\boldsymbol{F}_Q = -\frac{\delta W_Q}{\delta \boldsymbol{\xi}} = \pi\varepsilon a^2 \left(1 - \frac{a^2}{R^2}\right)(\boldsymbol{\nabla}|\boldsymbol{E}^{\text{ext}}|^2)_{\boldsymbol{\xi}} + \cdots \tag{S.1.38}$$

and

$$W_Q = -\pi\varepsilon a^2 \left[\left(1 - \frac{a^2}{R^2}\right)|\boldsymbol{E}^{\text{ext}}_{\boldsymbol{\xi}}|^2 + \tfrac{1}{4}a^2|(\boldsymbol{\nabla E}^{\text{ext}})_{\boldsymbol{\xi}}|^2\right] + \cdots, \tag{S.1.39}$$

where the terms neglected are of $O(a^4/R^4)$. Equations (S.1.38) and (S.1.39) are plotted in Fig. S.1 and compared against the exact solution of the Laplace problem using bipolar coordinates, showing excellent agreement when a/R is sufficiently small (for details on the bipolar solution, see §S.2). In the plots, the force and energy are rendered dimensionless using the same scales defined in Fig. 5 of the main text. Additionally, the variables are appropriately stretched so that Eq. (S.1.38) or (S.1.39) appears as a single curve.

For a fixed distribution of potentials on the outer shell, we obtain instead

$$\boldsymbol{F}_{\mathcal{V}} = \frac{\delta W_{\mathcal{V}}}{\delta \boldsymbol{\xi}} = \pi\varepsilon a^2 \left(1 + \frac{a^2}{R^2}\right)(\boldsymbol{\nabla}|\boldsymbol{E}^{\text{ext}}|^2)_{\boldsymbol{\xi}} + \cdots \tag{S.1.40}$$

and

$$W_{\mathcal{V}} = \pi\varepsilon a^2 \left[\left(1 + \frac{a^2}{R^2}\right)|\boldsymbol{E}^{\text{ext}}_{\boldsymbol{\xi}}|^2 + \tfrac{1}{4}a^2|(\boldsymbol{\nabla E}^{\text{ext}})_{\boldsymbol{\xi}}|^2\right] + \cdots. \tag{S.1.41}$$

These expressions are plotted in Fig. S.2 and compared to the bipolar solution (discussed in §S.2). Notably, the discrepancy between the reflections expansion and the bipolar solution as $a/R \to 1$ is larger in the fixed-potential problem (Fig. S.2) compared to the fixed-charge problem (Fig. S.1).

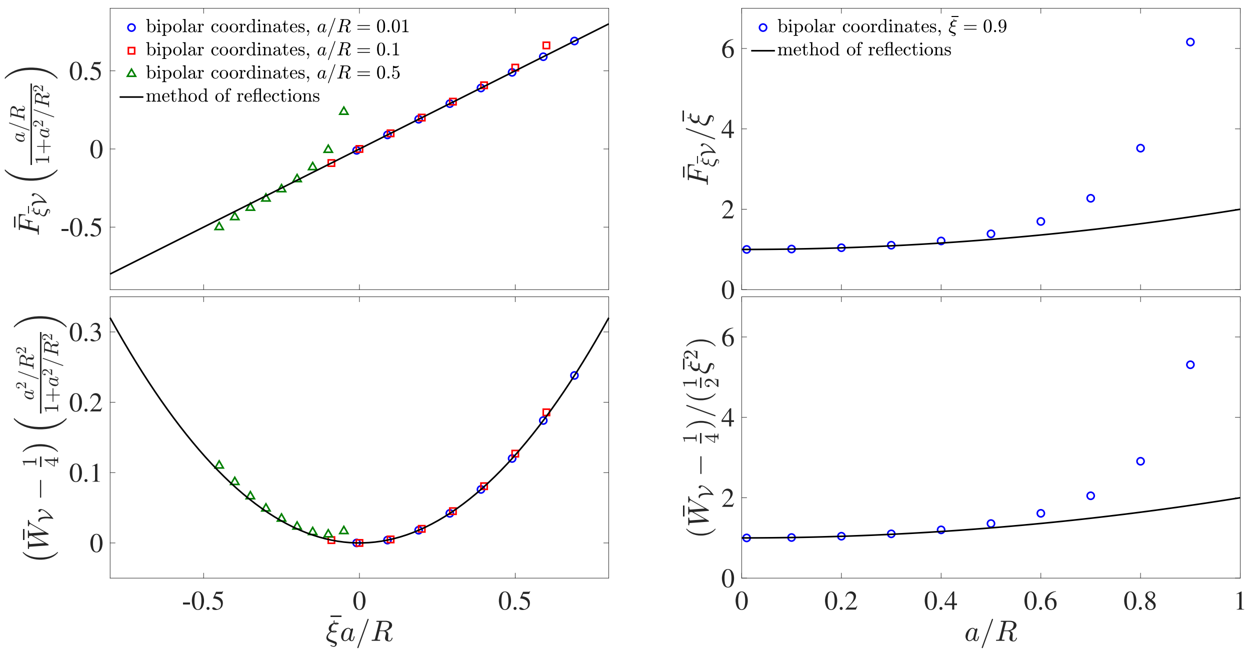

Figure S.2 Electric force and energy for a conducting particle held in a fixed system of potentials. The variables have the same meaning as in Fig. S.1.

Several remarks on Eqs. (S.1.38)-(S.1.41) are in order. First, it is clear that conducting particles are attracted to shells held at a fixed distribution of potentials and repelled by a fixed distribution of charges. These effects are captured by the $O(a^2/R^2)$ finite-size correction in the reflections expansion; a comparison to the (exact) bipolar solution shows that this level of approximation is adequate if $a/R \ll 1$. Second, we find $\boldsymbol{F}_Q \neq \boldsymbol{F}_{\mathcal{V}}$ and $W_Q \neq -W_{\mathcal{V}}$ for shells of finite size, contrary to the limiting result for a semi-infinite medium. It remains true, however, that W_Q and $-W_{\mathcal{V}}$ are equivalent to the particle's potential energy when, respectively, the charges and potentials are held fixed on the outer shell. In the latter case, the external work done to maintain the shell potential is $2W_{\mathcal{V}}$, as shown in the main text [cf. Eq. (3.22)].

S.1.2 Insulating particle

Insulating particles are discussed in §3.2 in the main text. For such particles, the Dirichlet condition (3.10) at $r' = a$ is replaced by the Neumann condition,

$$\hat{\boldsymbol{n}} \cdot \boldsymbol{\nabla}\psi = 0 \quad \text{at} \quad r' = a. \tag{3.25}$$

Once again, the solution for the potential ψ can be obtained using the method of reflections. The solution procedure is essentially the same as the one described in §S.1.1. The key difference to recognize is that the insulating Neumann condition (3.25) induces reflected modes $-(\psi^{(1)} + \psi^{(2)})$, $-(\psi^{(5)} + \psi^{(6)})$, etc. of opposite sign compared to the conducting Dirichlet condition (3.10). We may immediately deduce this result by applying Eqs. (S.A.5)-(S.A.6) to the boundary condition at the particle. To avoid redundancy, we shall not describe this calculation in any detail.

Instead, we shall simply present the final results for insulators that are analogous to Eqs. (S.1.38)-(S.1.41) for conductors. For fixed charges on the outer shell, we find

$$\boldsymbol{F}_Q = -\frac{\delta W_Q}{\delta \boldsymbol{\xi}} = -\pi\varepsilon a^2 \left(1 + \frac{a^2}{R^2}\right) (\boldsymbol{\nabla}|\boldsymbol{E}^{\mathrm{ext}}|^2)_{\xi} + \cdots \tag{S.1.42}$$

and

$$W_Q = \pi\varepsilon a^2 \left[\left(1 + \frac{a^2}{R^2}\right) |\boldsymbol{E}_{\xi}^{\mathrm{ext}}|^2 + \tfrac{1}{4} a^2 |(\boldsymbol{\nabla}\boldsymbol{E}^{\mathrm{ext}})_{\xi}|^2\right] + \cdots, \tag{S.1.43}$$

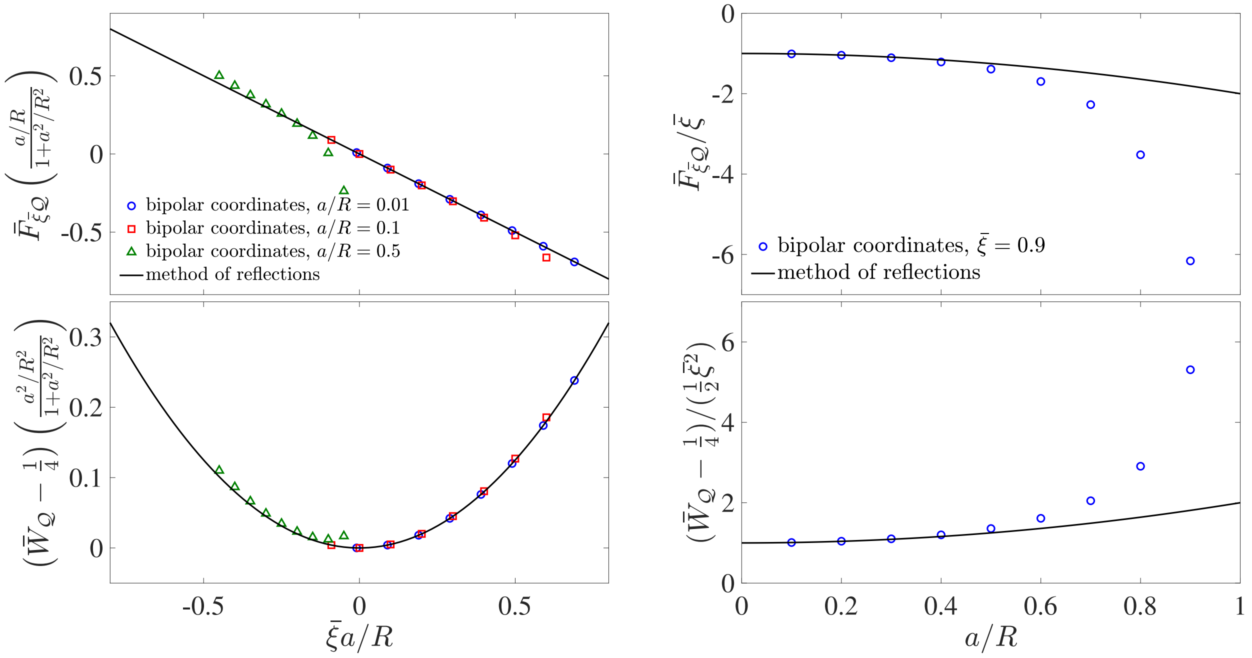

Figure S.3 Electric force and energy for an insulating particle held in a fixed system of charges. The variables have the same meaning as in Fig. S.1.

whereas, for fixed potentials, we find instead

$$F_{\mathcal{V}} = \frac{\delta W_{\mathcal{V}}}{\delta \boldsymbol{\xi}} = -\pi\varepsilon a^2 \left(1 - \frac{a^2}{R^2}\right)(\boldsymbol{\nabla}|\boldsymbol{E}^{\text{ext}}|^2)_{\boldsymbol{\xi}} + \cdots \tag{S.1.44}$$

and

$$W_{\mathcal{V}} = -\pi\varepsilon a^2 \left[\left(1 - \frac{a^2}{R^2}\right)|\boldsymbol{E}^{\text{ext}}_{\boldsymbol{\xi}}|^2 + \tfrac{1}{4}a^2|(\boldsymbol{\nabla}\boldsymbol{E}^{\text{ext}})_{\boldsymbol{\xi}}|^2\right] + \cdots . \tag{S.1.45}$$

Equations (S.1.42)-(S.1.43) and (S.1.44)-(S.1.45) are plotted in Figs. S.3 and S.4, respectively. The leading-order terms can be found in Eqs. (3.27)-(3.28) in the main text. It is interesting to note that W_Q and $W_{\mathcal{V}}$ for insulators are, respectively, identical to $W_{\mathcal{V}}$ and W_Q for conductors. As anticipated, insulators are attracted to fixed charges and repelled by fixed potentials.

To summarize, we have described an approximate solution to the Laplace problem, both for conducting and insulating particles, using the method of reflections. We have validated this reflections solution using an exact solution in bipolar coordinates; the numerical data from the bipolar solution are plotted as markers in Figs. S.1-S.4. Details on the bipolar solution are described in the following section.

S.2 Electric problem: bipolar coordinates

The Laplace problem can be solved exactly using bipolar coordinates (σ, τ). Previously, Liu *et al.*[5] applied eigenfunction expansions in bipolar coordinates in order to solve the 2D electric Laplace problem for a fixed potential distribution on the outer shell. Here, we apply the same method and expand upon their analysis by additionally considering a fixed distribution of charges on the outer shell. Our main purpose in developing an exact solution in bipolar coordinates is to validate the analytical expressions derived for the electric force [Eqs. (S.1.38), (S.1.40), (S.1.42), and (S.1.44)] and energy [Eqs. (S.1.39), (S.1.41), (S.1.43), and (S.1.45)] using the method of reflections.

S.2.1 Governing equations

The transformation from Cartesian coordinates (x, y) to bipolar coordinates (σ, τ) can be found in numerous texts;[3] for details, the reader is referred to Appendix S.B of this supplemental document. In bipolar coordinates, the Laplace problem (3.8)-(3.11)

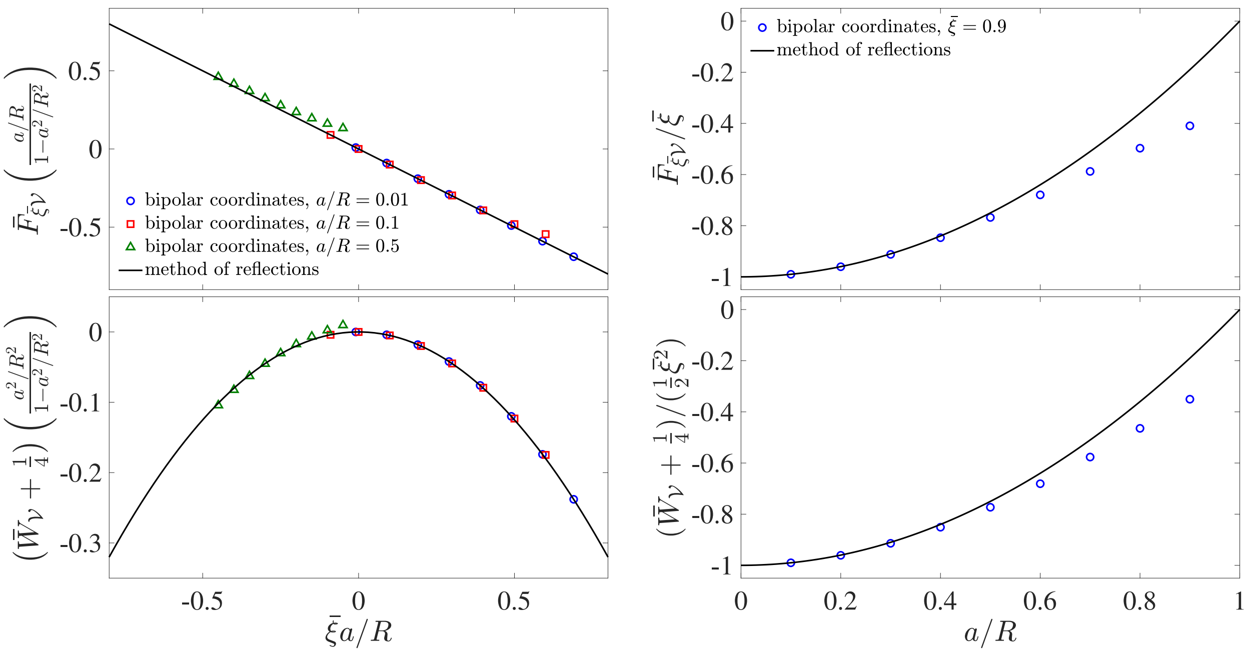

Figure S.4 Electric force and energy for an insulating particle held in a fixed system of potentials. The variables have the same meaning as in Fig. S.1.

for conducting particles becomes

$$\frac{\partial^2 \psi}{\partial \sigma^2} + \frac{\partial^2 \psi}{\partial \tau^2} = 0 \quad \text{for} \quad \tau_R \le \tau \le \tau_a, \tag{S.2.1}$$

with

either
$$\frac{\partial \psi_Q}{\partial \tau} = \frac{\partial \psi^{\text{ext}}}{\partial \tau} \quad \text{at} \quad \tau = \tau_R \tag{S.2.2a}$$

or
$$\psi_{\mathcal{V}} = \psi^{\text{ext}} \quad \text{at} \quad \tau = \tau_R, \tag{S.2.2b}$$

and

$$\frac{\partial \psi}{\partial \sigma} = 0 \quad \text{at} \quad \tau = \tau_a, \tag{S.2.3}$$

$$Q = \varepsilon \int_0^{2\pi} \left.\frac{\partial \psi}{\partial \tau}\right|_{\tau=\tau_a} \mathrm{d}\sigma = 0. \tag{S.2.4}$$

The problem for insulating particles is similar, except that Eq. (S.2.3) is replaced by

$$\frac{\partial \psi}{\partial \tau} = 0 \quad \text{at} \quad \tau = \tau_a, \tag{S.2.5}$$

which is equivalent to Eq. (3.25) from the main text. If the charge density on the outer shell is specified [Eq. (S.2.2a)], then we additionally require that the average potential on the outer shell vanishes:

$$\langle \psi \rangle_R = \frac{1}{2\pi} \int_0^{2\pi} \frac{\psi|_{\tau=\tau_R} \sinh \tau_R}{\cosh \tau_R - \cos \sigma} \mathrm{d}\sigma = 0, \tag{S.2.6}$$

where we have used $R = c/\sinh \tau_R$ to eliminate the shell radius R. The last condition represents a choice of reference potential.

To calculate the force, equation (3.7) may be expressed in bipolar coordinates as

$$\boldsymbol{F} = -\varepsilon \int_0^{2\pi} \left(\frac{\partial \psi}{\partial \sigma} \frac{\partial \psi}{\partial \tau} \hat{\boldsymbol{\sigma}} + \left\{ \left(\frac{\partial \psi}{\partial \tau}\right)^2 - \frac{1}{2} \left[\left(\frac{\partial \psi}{\partial \sigma}\right)^2 + \left(\frac{\partial \psi}{\partial \tau}\right)^2 \right] \right\} \hat{\boldsymbol{\tau}} \right) \Bigg|_{\tau=\tau_a} \frac{(\cosh \tau_a - \cos \sigma)\, \mathrm{d}\sigma}{c}. \tag{S.2.7}$$

Finally, to calculate the energy, equation (S.1.9) may be expressed as

$$W = \tfrac{1}{2}\varepsilon \int_0^{2\pi} \psi \frac{\partial \psi}{\partial \tau}\bigg|_{\tau=\tau_a} \mathrm{d}\sigma - \tfrac{1}{2}\varepsilon \int_0^{2\pi} \left(\psi \frac{\partial \psi}{\partial \tau} - \psi^{\mathrm{ext}} \frac{\partial \psi^{\mathrm{ext}}}{\partial \tau} \right)\bigg|_{\tau=\tau_R} \mathrm{d}\sigma. \tag{S.2.8}$$

Below, we derive a solution for ψ by means of eigenfunction expansions.

S.2.2 Eigenfunction expansions

To solve Eqs. (S.2.1)-(S.2.6) above, we must first represent the external potential ψ^{ext} and its normal derivative $\partial\psi^{\mathrm{ext}}/\partial\tau$ at the outer shell $\tau = \tau_R$ in terms of orthogonal modes:

$$\psi^{\mathrm{ext}}|_{\tau=\tau_R} = a_0 + \sum_{n=1}^{\infty} \left[a_n \cos(n\sigma) + b_n \sin(n\sigma)\right], \tag{S.2.9}$$

$$\frac{\partial \psi^{\mathrm{ext}}}{\partial \tau}\bigg|_{\tau=\tau_R} = \sum_{n=1}^{\infty} n \left[c_n \cos(n\sigma) + d_n \sin(n\sigma)\right], \tag{S.2.10}$$

where the coefficients a_n, b_n, c_n, and d_n are given by the inversion theorem,

$$a_0 = \frac{1}{2\pi} \int_0^{2\pi} \psi^{\mathrm{ext}}|_{\tau=\tau_R} \,\mathrm{d}\sigma, \tag{S.2.11a}$$

$$a_n = \frac{1}{\pi} \int_0^{2\pi} \psi^{\mathrm{ext}}|_{\tau=\tau_R} \cos(n\sigma)\,\mathrm{d}\sigma, \qquad n = 1, 2, \ldots, \tag{S.2.11b}$$

$$b_n = \frac{1}{\pi} \int_0^{2\pi} \psi^{\mathrm{ext}}|_{\tau=\tau_R} \sin(n\sigma)\,\mathrm{d}\sigma, \qquad n = 1, 2, \ldots, \tag{S.2.11c}$$

$$c_n = \frac{1}{n\pi} \int_0^{2\pi} \frac{\partial \psi^{\mathrm{ext}}}{\partial \tau}\bigg|_{\tau=\tau_R} \cos(n\sigma)\,\mathrm{d}\sigma, \qquad n = 1, 2, \ldots, \tag{S.2.11d}$$

$$d_n = \frac{1}{n\pi} \int_0^{2\pi} \frac{\partial \psi^{\mathrm{ext}}}{\partial \tau}\bigg|_{\tau=\tau_R} \sin(n\sigma)\,\mathrm{d}\sigma, \qquad n = 1, 2, \ldots. \tag{S.2.11e}$$

Then, we may expand the potential ψ in terms of eigenfunctions of Laplace's equation:

$$\psi = \psi_0 + \sum_{n=1}^{\infty} \left[\left(\psi_n^+ \mathrm{e}^{n\tau} + \psi_n^- \mathrm{e}^{-n\tau}\right) \cos(n\sigma) + \left(\phi_n^+ \mathrm{e}^{n\tau} + \phi_n^- \mathrm{e}^{-n\tau}\right) \sin(n\sigma) \right], \tag{S.2.12}$$

where the coefficients ψ_0, ψ_n^+, ψ_n^-, ϕ_n^+, and ϕ_n^- are to be determined from the boundary conditions. Note that the zero-charge condition (S.2.4) is automatically satisfied by the general solution (S.2.12). Substituting Eq. (S.2.12) into (S.2.6) gives the average potential on the outer shell,

$$\langle \psi \rangle_R = \psi_0 + \sum_{n=1}^{\infty} \mathrm{e}^{-n\tau_R} \left(\psi_n^+ \mathrm{e}^{n\tau_R} + \psi_n^- \mathrm{e}^{-n\tau_R}\right). \tag{S.2.13}$$

Equations (S.2.7)-(S.2.8) for the force $\boldsymbol{F}$ and energy W may also be written as double series in terms of the coefficients ψ_n^+, ψ_n^-, ϕ_n^+, and ϕ_n^- from the eigenfunction expansions. However, it is more expedient to simply apply a numerical quadrature to the integrals in (S.2.7)-(S.2.8).

S.2.3 Solution of the series coefficients

To solve for the series coefficients ψ_0, ψ_n^+, ψ_n^-, ϕ_n^+, and ϕ_n^-, we first substitute the general solution (S.2.12) into the boundary conditions (S.2.2)-(S.2.5) and resolve the equations into orthogonal modes. This yields a system of linear equations that can be uniquely solved for ψ_0, ψ_n^+, ψ_n^-, ϕ_n^+, and ϕ_n^-. For *conducting particles*, we obtain the system of equations,

either $$\psi_{\mathcal{Q}0} = -\sum_{n=1}^{\infty} \mathrm{e}^{-n\tau_R} \left(\psi_{\mathcal{Q}n}^+ \mathrm{e}^{n\tau_R} + \psi_{\mathcal{Q}n}^- \mathrm{e}^{-n\tau_R}\right), \quad \psi_{\mathcal{Q}n}^+ \mathrm{e}^{n\tau_R} - \psi_{\mathcal{Q}n}^- \mathrm{e}^{-n\tau_R} = c_n, \quad \phi_{\mathcal{Q}n}^+ \mathrm{e}^{n\tau_R} - \phi_{\mathcal{Q}n}^- \mathrm{e}^{-n\tau_R} = d_n \tag{S.2.14a}$$

or $$\psi_{\mathcal{V}0} = a_0, \quad \psi_{\mathcal{V}n}^+ \mathrm{e}^{n\tau_R} + \psi_{\mathcal{V}n}^- \mathrm{e}^{-n\tau_R} = a_n, \quad \phi_{\mathcal{V}n}^+ \mathrm{e}^{n\tau_R} + \phi_{\mathcal{V}n}^- \mathrm{e}^{-n\tau_R} = b_n, \tag{S.2.14b}$$

and

$$\psi_n^+ \mathrm{e}^{n\tau_a} + \psi_n^- \mathrm{e}^{-n\tau_a} = 0, \qquad \phi_n^+ \mathrm{e}^{n\tau_a} + \phi_n^- \mathrm{e}^{-n\tau_a} = 0, \tag{S.2.15}$$

which are derived from Eqs. (S.2.2)-(S.2.3) (here, it is implied that $n = 1, 2, \dots$). Notice that, for the fixed-charge case, the constant potential ψ_{Q0} is chosen to ensure that the average potential on the outer shell is zero. Solving Eqs. (S.2.14)-(S.2.15) yields series coefficients,

$$\psi_{Q0} = -\sum_{n=1}^{\infty} c_n \mathrm{e}^{-n\tau_R} \left(\frac{\mathrm{e}^{-n(\tau_a-\tau_R)} - \mathrm{e}^{n(\tau_a-\tau_R)}}{\mathrm{e}^{-n(\tau_a-\tau_R)} + \mathrm{e}^{n(\tau_a-\tau_R)}} \right), \qquad \psi_{\mathcal{V}0} = a_0, \tag{S.2.16a}$$

$$\psi_{Qn}^+ = \frac{c_n \mathrm{e}^{-n\tau_a}}{\mathrm{e}^{-n(\tau_a-\tau_R)} + \mathrm{e}^{n(\tau_a-\tau_R)}}, \qquad \psi_{\mathcal{V}n}^+ = \frac{a_n \mathrm{e}^{-n\tau_a}}{\mathrm{e}^{-n(\tau_a-\tau_R)} - \mathrm{e}^{n(\tau_a-\tau_R)}}, \tag{S.2.16b}$$

$$\psi_{Qn}^- = -\frac{c_n \mathrm{e}^{n\tau_a}}{\mathrm{e}^{-n(\tau_a-\tau_R)} + \mathrm{e}^{n(\tau_a-\tau_R)}}, \qquad \psi_{\mathcal{V}n}^- = -\frac{a_n \mathrm{e}^{n\tau_a}}{\mathrm{e}^{-n(\tau_a-\tau_R)} - \mathrm{e}^{n(\tau_a-\tau_R)}}, \tag{S.2.16c}$$

$$\phi_{Qn}^+ = \frac{d_n \mathrm{e}^{-n\tau_a}}{\mathrm{e}^{-n(\tau_a-\tau_R)} + \mathrm{e}^{n(\tau_a-\tau_R)}}, \qquad \phi_{\mathcal{V}n}^+ = \frac{b_n \mathrm{e}^{-n\tau_a}}{\mathrm{e}^{-n(\tau_a-\tau_R)} - \mathrm{e}^{n(\tau_a-\tau_R)}}, \tag{S.2.16d}$$

$$\phi_{Qn}^- = -\frac{d_n \mathrm{e}^{n\tau_a}}{\mathrm{e}^{-n(\tau_a-\tau_R)} + \mathrm{e}^{n(\tau_a-\tau_R)}}, \qquad \phi_{\mathcal{V}n}^- = -\frac{b_n \mathrm{e}^{n\tau_a}}{\mathrm{e}^{-n(\tau_a-\tau_R)} - \mathrm{e}^{n(\tau_a-\tau_R)}}. \tag{S.2.16e}$$

Substituting Eqs. (S.2.16) into (S.2.12) then yields the particular solution for the electric potential around a conducting particle.

For *insulating particles*, Eq. (S.2.15) is replaced by

$$\psi_n^+ \mathrm{e}^{n\tau_a} - \psi_n^- \mathrm{e}^{-n\tau_a} = 0, \qquad \phi_n^+ \mathrm{e}^{n\tau_a} - \phi_n^- \mathrm{e}^{-n\tau_a} = 0, \tag{S.2.17}$$

which can be derived from Eq. (S.2.5). The new coefficients are obtained by solving Eqs. (S.2.14) and (S.2.17) simultaneously:

$$\psi_{Q0} = -\sum_{n=1}^{\infty} c_n \mathrm{e}^{-n\tau_R} \left(\frac{\mathrm{e}^{-n(\tau_a-\tau_R)} + \mathrm{e}^{n(\tau_a-\tau_R)}}{\mathrm{e}^{-n(\tau_a-\tau_R)} - \mathrm{e}^{n(\tau_a-\tau_R)}} \right), \qquad \psi_{\mathcal{V}0} = a_0, \tag{S.2.18a}$$

$$\psi_{Qn}^+ = \frac{c_n \mathrm{e}^{-n\tau_a}}{\mathrm{e}^{-n(\tau_a-\tau_R)} - \mathrm{e}^{n(\tau_a-\tau_R)}}, \qquad \psi_{\mathcal{V}n}^+ = \frac{a_n \mathrm{e}^{-n\tau_a}}{\mathrm{e}^{-n(\tau_a-\tau_R)} + \mathrm{e}^{n(\tau_a-\tau_R)}}, \tag{S.2.18b}$$

$$\psi_{Qn}^- = \frac{c_n \mathrm{e}^{n\tau_a}}{\mathrm{e}^{-n(\tau_a-\tau_R)} - \mathrm{e}^{n(\tau_a-\tau_R)}}, \qquad \psi_{\mathcal{V}n}^- = \frac{a_n \mathrm{e}^{n\tau_a}}{\mathrm{e}^{-n(\tau_a-\tau_R)} + \mathrm{e}^{n(\tau_a-\tau_R)}}, \tag{S.2.18c}$$

$$\phi_{Qn}^+ = \frac{d_n \mathrm{e}^{-n\tau_a}}{\mathrm{e}^{-n(\tau_a-\tau_R)} - \mathrm{e}^{n(\tau_a-\tau_R)}}, \qquad \phi_{\mathcal{V}n}^+ = \frac{b_n \mathrm{e}^{-n\tau_a}}{\mathrm{e}^{-n(\tau_a-\tau_R)} + \mathrm{e}^{n(\tau_a-\tau_R)}}, \tag{S.2.18d}$$

$$\phi_{Qn}^- = \frac{d_n \mathrm{e}^{n\tau_a}}{\mathrm{e}^{-n(\tau_a-\tau_R)} - \mathrm{e}^{n(\tau_a-\tau_R)}}, \qquad \phi_{\mathcal{V}n}^- = \frac{b_n \mathrm{e}^{n\tau_a}}{\mathrm{e}^{-n(\tau_a-\tau_R)} + \mathrm{e}^{n(\tau_a-\tau_R)}}. \tag{S.2.18e}$$

This completes the solution for the electric potential for conducting and insulating particles.

S.2.4 Numerical procedure

Equations (S.2.7) and (S.2.8) may be evaluated numerically using the following procedure:

1. First, numerical values of a, R, and ξ are specified and the bipolar parameters c, τ_a, and τ_R are calculated using Eqs. (S.B.4)-(S.B.5) in Appendix S.B. The relevant geometric quantities are then given by Eqs. (S.B.6)-(S.B.10).

2. Second, the dielectric permittivity ε is specified (for convenience, we set $\varepsilon = 1$) and the field coefficients $\boldsymbol{E}_\mathbf{0}^{\text{ext}}$ and $(\boldsymbol{\nabla E}^{\text{ext}})_\mathbf{0}$ in Eq. (3.1) are expressed in the Cartesian basis,

$$\boldsymbol{E}_\mathbf{0}^{\text{ext}} = E_0 \left(\hat{\boldsymbol{x}} \cos\alpha_0 + \hat{\boldsymbol{y}} \sin\alpha_0 \right), \tag{S.2.19}$$

$$(\boldsymbol{\nabla E}^{\text{ext}})_\mathbf{0} = E_1 \left[(\hat{\boldsymbol{x}}\hat{\boldsymbol{x}} - \hat{\boldsymbol{y}}\hat{\boldsymbol{y}}) \cos(2\alpha_1) + (\hat{\boldsymbol{x}}\hat{\boldsymbol{y}} + \hat{\boldsymbol{y}}\hat{\boldsymbol{x}}) \sin(2\alpha_1) \right], \tag{S.2.20}$$

   using numerical values for the field amplitudes E_0, E_1 and phase angles α_0, α_1. Substituting Eqs. (S.2.19)-(S.2.20) and $\boldsymbol{r} = x\hat{\boldsymbol{x}} + y\hat{\boldsymbol{y}}$ into (3.1) then gives the external potential ψ^{ext} in terms of the Cartesian coordinates (x, y). This expression is then rewritten in terms of the bipolar coordinates (σ, τ) using the transformation rules (S.B.3) in Appendix S.B.

3. Third, the series coefficients a_n, b_n, c_n, and d_n appearing in Eqs. (S.2.9)-(S.2.10) are calculated by applying numerical quadratures to Eqs. (S.2.11).

4. Fourth, the series coefficients ψ_0, ψ_n^+, ψ_n^-, ϕ_n^+, and ϕ_n^- appearing in Eq. (S.2.12) are calculated using either Eqs. (S.2.16) (for conductors) or (S.2.18) (for insulators). The electric potential ψ and its derivatives $\partial\psi/\partial\sigma$, $\partial\psi/\partial\tau$ are then evaluated at the boundaries $\tau = \tau_a$ and τ_R.

5. Finally, the boundaries $\tau = \tau_a$ and τ_R are discretized and Eqs. (S.2.7)-(S.2.8) are approximated using numerical quadratures.

The numerical results for $\boldsymbol{F}$ and W are plotted in Figs. S.1-S.4 alongside the approximate results obtained via the method of reflections. For all of the calculations reported here, we set $E_0 = E_1 = 1$ and $\alpha_0 = \alpha_1 = 0$ in Eqs. (S.2.19)-(S.2.20).

S.3 Capillary problem: method of reflections

Table S.2 Analytical expressions for the capillary force and energy, including the first finite-size correction, for a particle held in a fixed system of heights ($\mathcal{U}$) or slopes ($\mathcal{P}$) [cf. Eqs. (S.3.41)-(S.3.48) and (S.3.54)-(S.3.55)].

Particle	Capillary Force	Capillary Energy
Pinned Contact Line (Non-Undulated)	$\boldsymbol{F}_{\mathcal{U}} = -\frac{1}{4}\pi\gamma a^4 \left(1 + \frac{a^4}{R^4}\right)(\nabla\lvert\boldsymbol{K}^{\text{ext}}\rvert^2)_{\xi} + \cdots$	$W_{\mathcal{U}} = \frac{1}{4}\pi\gamma a^4 \left[\left(1 + \frac{a^4}{R^4}\right)\lvert\boldsymbol{K}_{\xi}^{\text{ext}}\rvert^2 + \frac{1}{12}a^2\lvert(\nabla\boldsymbol{K}^{\text{ext}})_{\xi}\rvert^2\right] + \cdots$
	$\boldsymbol{F}_{\mathcal{P}} = -\frac{1}{4}\pi\gamma a^4 \left(1 - \frac{a^4}{R^4}\right)(\nabla\lvert\boldsymbol{K}^{\text{ext}}\rvert^2)_{\xi} + \cdots$	$W_{\mathcal{P}} = -\frac{1}{4}\pi\gamma a^4 \left[\left(1 - \frac{a^4}{R^4}\right)\lvert\boldsymbol{K}_{\xi}^{\text{ext}}\rvert^2 + \frac{1}{12}a^2\lvert(\nabla\boldsymbol{K}^{\text{ext}})_{\xi}\rvert^2\right] + \cdots$
Equilibrium Contact Angle (Cylinder, 90°)	$\boldsymbol{F}_{\mathcal{U}} = \frac{1}{4}\pi\gamma a^4 \left(1 - \frac{a^4}{R^4}\right)(\nabla\lvert\boldsymbol{K}^{\text{ext}}\rvert^2)_{\xi} + \cdots$	$W_{\mathcal{U}} = -\frac{1}{4}\pi\gamma a^4 \left[\left(1 - \frac{a^4}{R^4}\right)\lvert\boldsymbol{K}_{\xi}^{\text{ext}}\rvert^2 + \frac{1}{12}a^2\lvert(\nabla\boldsymbol{K}^{\text{ext}})_{\xi}\rvert^2\right] + \cdots$
	$\boldsymbol{F}_{\mathcal{P}} = \frac{1}{4}\pi\gamma a^4 \left(1 + \frac{a^4}{R^4}\right)(\nabla\lvert\boldsymbol{K}^{\text{ext}}\rvert^2)_{\xi} + \cdots$	$W_{\mathcal{P}} = \frac{1}{4}\pi\gamma a^4 \left[\left(1 + \frac{a^4}{R^4}\right)\lvert\boldsymbol{K}_{\xi}^{\text{ext}}\rvert^2 + \frac{1}{12}a^2\lvert(\nabla\boldsymbol{K}^{\text{ext}})_{\xi}\rvert^2\right] + \cdots$
Equilibrium Contact Angle (Sphere)	$\boldsymbol{F}_{\mathcal{U}} = \frac{1}{12}\pi\gamma a^4 \left(1 - \frac{1}{3}\frac{a^4}{R^4}\right)(\nabla\lvert\boldsymbol{K}^{\text{ext}}\rvert^2)_{\xi} + \cdots$	$W_{\mathcal{U}} = -\frac{1}{12}\pi\gamma a^4 \left[\left(1 - \frac{1}{3}\frac{a^4}{R^4}\right)\lvert\boldsymbol{K}_{\xi}^{\text{ext}}\rvert^2 + \frac{1}{8}a^2\lvert(\nabla\boldsymbol{K}^{\text{ext}})_{\xi}\rvert^2\right] + \cdots$
	$\boldsymbol{F}_{\mathcal{P}} = \frac{1}{12}\pi\gamma a^4 \left(1 + \frac{1}{3}\frac{a^4}{R^4}\right)(\nabla\lvert\boldsymbol{K}^{\text{ext}}\rvert^2)_{\xi} + \cdots$	$W_{\mathcal{P}} = \frac{1}{12}\pi\gamma a^4 \left[\left(1 + \frac{1}{3}\frac{a^4}{R^4}\right)\lvert\boldsymbol{K}_{\xi}^{\text{ext}}\rvert^2 + \frac{1}{8}a^2\lvert(\nabla\boldsymbol{K}^{\text{ext}})_{\xi}\rvert^2\right] + \cdots$

S.3.1 Particle with a pinned contact line

Below, we present supplemental details of the calculation for particles with pinned contact lines (discussed in §4.1 of the main text). For the most part, we focus on symmetrically pinned (i.e., non-undulated) contact lines; results for undulated contact lines are discussed in Appendix E of the main text.

S.3.1.1 Governing equations

Laplace problem: The boundary-value problem for a particle with a symmetrically pinned contact line is given by Eqs. (4.8)-(4.12) in the main text (reproduced here for convenience):

$$\nabla^2\zeta = 0 \quad \text{for} \quad r' \geq a \quad \text{and} \quad r \leq R, \tag{4.8}$$

where

either $$\zeta_{\mathcal{U}} = \zeta^{\text{ext}} \quad \text{at} \quad r = R \tag{4.9a}$$

or $$\hat{\boldsymbol{n}}\cdot\nabla\zeta_{\mathcal{P}} = \hat{\boldsymbol{n}}\cdot\nabla\zeta^{\text{ext}} \quad \text{at} \quad r = R, \tag{4.9b}$$

and

$$\zeta = U + \boldsymbol{\Omega}_{\times}\cdot\boldsymbol{r}' \quad \text{at} \quad r' = a, \tag{4.10}$$

$$P = -\gamma \oint_{r'=a} \hat{\boldsymbol{n}}\cdot\nabla\zeta \, \mathrm{d}s = 0, \tag{4.11}$$

$$\boldsymbol{N}_{\times} = -\gamma \oint_{r'=a} [\boldsymbol{r}'(\hat{\boldsymbol{n}}\cdot\nabla\zeta) - \zeta\hat{\boldsymbol{n}}] \, \mathrm{d}s = \boldsymbol{0}. \tag{4.12}$$

Here, ζ^{ext} is the host interface height given by Eq. (4.1), U is the vertical translation, $\boldsymbol{\Omega} = \boldsymbol{\epsilon}\cdot\boldsymbol{\Omega}_{\times}$ is the horizontal rotation, P is the vertical force, and $\boldsymbol{N} = \boldsymbol{\epsilon}\cdot\boldsymbol{N}_{\times}$ is the horizontal torque. In addition to the zero-monopole condition (4.11), we also have the constraint (4.12) on the dipole moment. This is what distinguishes the capillary problem from the electric problem.

Interface height: As in the electric problem, we pursue a solution of Eqs. (4.8)-(4.12) by the method of reflections:

$$\zeta = \sum_{n=0}^{\infty} \zeta^{(n)}, \tag{S.3.2}$$

$$U = \sum_{n \text{ odd}}^{\infty} U^{(n)}, \tag{S.3.3}$$

$$\boldsymbol{\Omega} = \sum_{n \text{ odd}}^{\infty} \boldsymbol{\Omega}^{(n)}. \tag{S.3.4}$$

The zeroth reflection is just the height of the host interface,

$$\zeta^{(0)} = \zeta^{\text{ext}} = \tfrac{1}{2}\boldsymbol{K}_{\boldsymbol{0}}^{\text{ext}} : \boldsymbol{r}\boldsymbol{r} + \tfrac{1}{6}(\boldsymbol{\nabla}\boldsymbol{K}^{\text{ext}})_{\boldsymbol{0}} \, (\cdot)^3 \, \boldsymbol{r}\boldsymbol{r}\boldsymbol{r} \tag{S.3.5}$$

[cf. Eq. (4.1)], and the $\zeta^{(n)}$, $U^{(n)}$, and $\boldsymbol{\Omega}^{(n)}$ satisfy

$$\left.\begin{aligned} &\zeta^{(n)} + \zeta^{(n-1)} = U^{(n)} + \boldsymbol{\Omega}_{\times}^{(n)} \cdot \boldsymbol{r} \\ &\oint_{r'=a} \hat{\boldsymbol{n}} \cdot \boldsymbol{\nabla}(\zeta^{(n)} + \zeta^{(n-1)}) \, \mathrm{d}s = 0 \\ &\oint_{r'=a} \left\{\boldsymbol{r}'[\hat{\boldsymbol{n}} \cdot \boldsymbol{\nabla}(\zeta^{(n)} + \zeta^{(n-1)})] - (\zeta^{(n)} + \zeta^{(n-1)})\hat{\boldsymbol{n}}\right\} \mathrm{d}s = \boldsymbol{0} \end{aligned}\right\} \quad \text{at} \quad r' = a, \qquad n = 1, 3, \ldots, \tag{S.3.6}$$

$$\left.\begin{aligned} &\zeta_{\mathcal{U}}^{(n)} + \zeta^{(n-1)} = 0 \\ &\hat{\boldsymbol{n}} \cdot \boldsymbol{\nabla}(\zeta_{\mathcal{P}}^{(n)} + \zeta^{(n-1)}) = 0 \end{aligned}\right\} \quad \text{at} \quad r = R, \qquad n = 2, 4, \ldots. \tag{S.3.7}$$

The even and odd reflections have the same meaning as in the electric problem.

Capillary force: The capillary force $\boldsymbol{F}$ (as defined via the stress tensor) is given by Eq. (4.7) in the main text:

$$\boldsymbol{F} = -\gamma \oint_{r'=a} \left[(\hat{\boldsymbol{n}} \cdot \boldsymbol{\nabla}\zeta)\boldsymbol{\nabla}\zeta - \tfrac{1}{2}|\boldsymbol{\nabla}\zeta|^2\hat{\boldsymbol{n}}\right] \mathrm{d}s. \tag{4.7}$$

Substituting Eq. (S.3.2) into (4.7) then gives

$$\boldsymbol{F} \equiv \sum_{n=0}^{\infty}\sum_{m=0}^{n} \boldsymbol{F}^{(mn)} = -\gamma \sum_{n=0}^{\infty}\sum_{m=0}^{\infty} \oint_{r'=a} \left[(\hat{\boldsymbol{n}} \cdot \boldsymbol{\nabla}\zeta^{(m)})\boldsymbol{\nabla}\zeta^{(n)} - \tfrac{1}{2}(\boldsymbol{\nabla}\zeta^{(m)} \cdot \boldsymbol{\nabla}\zeta^{(n)})\hat{\boldsymbol{n}}\right] \mathrm{d}s, \tag{S.3.8}$$

similar to (S.1.7). The first term in the series (S.3.8) is

$$\begin{aligned} \boldsymbol{F}^{(00)} &= -\gamma \oint_{r'=a} \hat{\boldsymbol{n}} \cdot \left(\boldsymbol{\nabla}\zeta^{(0)}\boldsymbol{\nabla}\zeta^{(0)} - \tfrac{1}{2}|\boldsymbol{\nabla}\zeta^{(0)}|^2\boldsymbol{\delta}\right) \mathrm{d}s \\ &= \boldsymbol{0}, \end{aligned} \tag{S.3.9}$$

as expected.

Capillary energy: The capillary energy W is given by Eq. (4.5),

$$W = \tfrac{1}{2}\gamma \iint_{\substack{r' \geq a \\ r \leq R}} |\boldsymbol{\nabla}\zeta|^2 \, \mathrm{d}^2\boldsymbol{r} - \tfrac{1}{2}\gamma \iint_{r \leq R} |\boldsymbol{\nabla}\zeta^{\text{ext}}|^2 \, \mathrm{d}^2\boldsymbol{r} + \tfrac{1}{2}\pi\gamma a^2 \Omega^2. \tag{4.5}$$

Integrating by parts gives

$$W = -\tfrac{1}{2}\gamma \oint_{r'=a} \zeta(\hat{\boldsymbol{n}} \cdot \boldsymbol{\nabla}\zeta)\mathrm{d}s - \tfrac{1}{2}\gamma \oint_{r=R} \hat{\boldsymbol{n}} \cdot (\zeta\boldsymbol{\nabla}\zeta - \zeta^{\text{ext}}\boldsymbol{\nabla}\zeta^{\text{ext}}) \, \mathrm{d}s + \tfrac{1}{2}\pi\gamma a^2 \Omega^2, \tag{S.3.10}$$

where we have used $\nabla^2\zeta = \nabla^2\zeta^{\text{ext}} = 0$ to eliminate the area integrals. Substituting Eqs. (S.3.2) and (S.3.4) into (S.3.10) for ζ and $\boldsymbol{\Omega}$, respectively, and setting $\zeta^{\text{ext}} = \zeta^{(0)}$ yields the double-series expansion,

$$W \equiv \sum_{n=0}^{\infty}\sum_{m=0}^{n} W^{(mn)} = -\tfrac{1}{2}\gamma \sum_{n=0}^{\infty}\sum_{m=0}^{\infty}\left(\oint_{r'=a} \zeta^{(m)}(\hat{\boldsymbol{n}}\cdot\nabla\zeta^{(n)})\mathrm{d}s + \oint_{r=R} \hat{\boldsymbol{n}}\cdot(\zeta^{(m)}\nabla\zeta^{(n)} - \zeta^{(0)}\nabla\zeta^{(0)})\,\mathrm{d}s - \pi a^2 \Omega^{(m)}\Omega^{(n)}\right), \tag{S.3.11}$$

similar to (S.1.10) (it is implied that $\Omega^{(n)}$ depends upon $\zeta^{(n)}$ and $\Omega^{(n)} = 0$ for even values of n). Using Eqs. (S.3.5) and (S.3.11), we find the "self energy" due to $\zeta^{(0)}$ is given by

$$\begin{aligned} W^{(00)} &= -\tfrac{1}{2}\gamma \oint_{r'=a} \zeta^{(0)}(\hat{\boldsymbol{n}}\cdot\nabla\zeta^{(0)})\,\mathrm{d}s \\ &= -\tfrac{1}{8}\pi\gamma a^4\left[|\boldsymbol{K}^{\text{ext}}_{\boldsymbol{\xi}}|^2 + \tfrac{1}{12}a^2|(\nabla\boldsymbol{K}^{\text{ext}})_{\boldsymbol{\xi}}|^2\right] - \tfrac{1}{2}\pi a^2\left|\boldsymbol{K}^{\text{ext}}_{\mathbf{0}}\cdot\boldsymbol{\xi} + \tfrac{1}{2}(\nabla\boldsymbol{K}^{\text{ext}})_{\mathbf{0}} : \boldsymbol{\xi}\boldsymbol{\xi}\right|^2. \end{aligned} \tag{S.3.12}$$

Adding the "trapping energy" $-\pi\gamma a^2$ to $W^{(00)}$ gives the total work needed to eliminate a patch of interfacial area. We shall soon see that the boundary perturbation due to rotation of the particle out of the undeformed plane contributes a term to the energy that exactly cancels the last term in Eq. (S.3.12). The leading contribution to the particle rotation is determined by the first reflection, which we consider next.

S.3.1.2 First reflection

The first reflection $\zeta^{(1)}$ is added to correct the particle boundary conditions (4.10)-(4.12). Hence, it must satisfy Laplace's equation

$$\nabla^2\zeta^{(1)} = 0 \tag{S.3.13}$$

and the boundary conditions

$$\zeta^{(1)} + \zeta^{(0)} = U^{(1)} + \boldsymbol{\Omega}^{(1)}_{\times}\cdot\boldsymbol{r}' \quad \text{at} \quad r' = a, \tag{S.3.14}$$

$$\oint_{r'=a} \hat{\boldsymbol{n}}\cdot\nabla(\zeta^{(1)} + \zeta^{(0)})\,\mathrm{d}s = 0, \tag{S.3.15}$$

$$\oint_{r'=a} \left\{\boldsymbol{r}'[\hat{\boldsymbol{n}}\cdot\nabla(\zeta^{(1)} + \zeta^{(0)})] - (\zeta^{(1)} + \zeta^{(0)})\hat{\boldsymbol{n}}\right\}\mathrm{d}s = \mathbf{0}. \tag{S.3.16}$$

Recasting $\zeta^{(0)}$ [Eq. (S.3.5)] in terms of $\boldsymbol{r}' = \boldsymbol{r} - \boldsymbol{\xi}$ gives

$$\zeta^{(0)} = \tfrac{1}{2}\boldsymbol{K}^{\text{ext}}_{\mathbf{0}} : \boldsymbol{\xi}\boldsymbol{\xi} + \tfrac{1}{6}(\nabla\boldsymbol{K}^{\text{ext}})_{\mathbf{0}}(\cdot)^3\boldsymbol{\xi}\boldsymbol{\xi}\boldsymbol{\xi} + [\boldsymbol{K}^{\text{ext}}_{\mathbf{0}}\cdot\boldsymbol{\xi} + \tfrac{1}{2}(\nabla\boldsymbol{K}^{\text{ext}})_{\mathbf{0}} : \boldsymbol{\xi}\boldsymbol{\xi}]\cdot\boldsymbol{r}' + \tfrac{1}{2}\boldsymbol{K}^{\text{ext}}_{\boldsymbol{\xi}} : \boldsymbol{r}'\boldsymbol{r}' + \tfrac{1}{6}(\nabla\boldsymbol{K}^{\text{ext}})_{\boldsymbol{\xi}}(\cdot)^3\boldsymbol{r}'\boldsymbol{r}'\boldsymbol{r}', \tag{S.3.17}$$

where we have defined the external curvature and curvature gradient at the particle's center,

$$\boldsymbol{K}^{\text{ext}}_{\boldsymbol{\xi}} = \boldsymbol{K}^{\text{ext}}_{\mathbf{0}} + (\nabla\boldsymbol{K}^{\text{ext}})_{\mathbf{0}}\cdot\boldsymbol{\xi}, \tag{S.3.18}$$

$$(\nabla\boldsymbol{K}^{\text{ext}})_{\boldsymbol{\xi}} = (\nabla\boldsymbol{K}^{\text{ext}})_{\mathbf{0}}. \tag{S.3.19}$$

Substituting Eq. (S.3.17) into (S.3.14)-(S.3.16) and integrating yields the solution

$$\zeta^{(1)} = -\tfrac{1}{2}a^4\boldsymbol{K}^{\text{ext}}_{\boldsymbol{\xi}} : \frac{\boldsymbol{r}'\boldsymbol{r}'}{r'^4} - \tfrac{1}{6}a^6(\nabla\boldsymbol{K}^{\text{ext}})_{\boldsymbol{\xi}}(\cdot)^3\frac{\boldsymbol{r}'\boldsymbol{r}'\boldsymbol{r}'}{r'^6}, \tag{S.3.20}$$

$$U^{(1)} = \tfrac{1}{2}\boldsymbol{K}^{\text{ext}}_{\mathbf{0}} : \boldsymbol{\xi}\boldsymbol{\xi} + \tfrac{1}{6}(\nabla\boldsymbol{K}^{\text{ext}})_{\mathbf{0}}(\cdot)^3\boldsymbol{\xi}\boldsymbol{\xi}\boldsymbol{\xi}, \tag{S.3.21}$$

$$\boldsymbol{\Omega}^{(1)}_{\times} = \boldsymbol{K}^{\text{ext}}_{\mathbf{0}}\cdot\boldsymbol{\xi} + \tfrac{1}{2}(\nabla\boldsymbol{K}^{\text{ext}})_{\mathbf{0}} : \boldsymbol{\xi}\boldsymbol{\xi}. \tag{S.3.22}$$

It is straightforward to verify that Eqs. (S.3.20)-(S.3.22) uniquely satisfy (S.3.13)-(S.3.16). Thus, the first reflection induces quadrupolar and octupolar disturbances to the interface height. The sum $\zeta^{(0)} + \zeta^{(1)}$ represents the height of a semi-infinite interface, for which $a/R = 0$.

As with the electric problem, it is the coupling between the zeroth and first reflections that gives the leading contribution to the force:

$$\begin{aligned}\boldsymbol{F}^{(01)} &= -\gamma \oint_{r'=a} \hat{\boldsymbol{n}} \cdot \left[\boldsymbol{\nabla}\zeta^{(0)}\boldsymbol{\nabla}\zeta^{(1)} + \boldsymbol{\nabla}\zeta^{(1)}\boldsymbol{\nabla}\zeta^{(0)} - (\boldsymbol{\nabla}\zeta^{(0)} \cdot \boldsymbol{\nabla}\zeta^{(1)})\boldsymbol{\delta}\right] \mathrm{d}s \\ &= -\tfrac{1}{2}\pi\gamma a^4 \boldsymbol{K}_{\boldsymbol{\xi}}^{\mathrm{ext}} : (\boldsymbol{\nabla}\boldsymbol{K}^{\mathrm{ext}})_{\boldsymbol{\xi}}, \qquad \text{(S.3.23)}\\ \boldsymbol{F}^{(11)} &= -\gamma \oint_{r'=a} \hat{\boldsymbol{n}} \cdot \left(\boldsymbol{\nabla}\zeta^{(1)}\boldsymbol{\nabla}\zeta^{(1)} - \tfrac{1}{2}|\boldsymbol{\nabla}\zeta^{(1)}|^2\boldsymbol{\delta}\right) \mathrm{d}s \\ &= \boldsymbol{0}. \qquad \text{(S.3.24)}\end{aligned}$$

We may also calculate the following contributions to the energy:

$$\begin{aligned}W^{(01)} &= -\gamma \oint_{r'=a} \zeta^{(0)}(\hat{\boldsymbol{n}} \cdot \boldsymbol{\nabla}\zeta^{(1)})\mathrm{d}s - \gamma \oint_{r=R} \zeta^{(0)}(\hat{\boldsymbol{n}} \cdot \boldsymbol{\nabla}\zeta^{(1)})\,\mathrm{d}s \\ &= 0, \qquad \text{(S.3.25)}\\ W^{(11)} &= -\tfrac{1}{2}\gamma \oint_{r'=a} \zeta^{(1)}(\hat{\boldsymbol{n}} \cdot \boldsymbol{\nabla}\zeta^{(1)})\mathrm{d}s - \tfrac{1}{2}\gamma \oint_{r=R} \zeta^{(1)}(\hat{\boldsymbol{n}} \cdot \boldsymbol{\nabla}\zeta^{(1)})\,\mathrm{d}s + \tfrac{1}{2}\pi(\Omega^{(1)})^2 \\ &\simeq \tfrac{1}{8}\pi\gamma a^4 \left[|\boldsymbol{K}_{\boldsymbol{\xi}}^{\mathrm{ext}}|^2 + \tfrac{1}{12}a^2|(\boldsymbol{\nabla}\boldsymbol{K}^{\mathrm{ext}})_{\boldsymbol{\xi}}|^2\right] + \tfrac{1}{2}\pi a^2\left|\boldsymbol{K}_{\boldsymbol{0}}^{\mathrm{ext}} \cdot \boldsymbol{\xi} + \tfrac{1}{2}(\boldsymbol{\nabla}\boldsymbol{K}^{\mathrm{ext}})_{\boldsymbol{0}} : \boldsymbol{\xi}\boldsymbol{\xi}\right|^2, \qquad \text{(S.3.26)}\end{aligned}$$

where $O(a^4/R^4)$ corrections have been neglected in the last equation. Notice that the apparent boundary stretching due to particle rotation is responsible for the last term in Eq. (S.3.26). Comparison to Eq. (S.3.12) reveals that $W^{(00)} + W^{(11)} \simeq 0$ – exactly the same result as in the electric problem!

S.3.1.3 Second reflection

Next, we must compute the second reflection $\zeta^{(2)}$ as the solution of the Laplace problem,

$$\nabla^2\zeta^{(2)} = 0 \qquad \text{(S.3.27)}$$

with

either $$\zeta_{\mathcal{U}}^{(2)} + \zeta^{(1)} = 0 \quad \text{at} \quad r = R \qquad \text{(S.3.28a)}$$

or $$\hat{\boldsymbol{n}} \cdot \boldsymbol{\nabla}(\zeta_{\mathcal{P}}^{(2)} + \zeta^{(1)}) = 0 \quad \text{at} \quad r = R. \qquad \text{(S.3.28b)}$$

First, we recast $\zeta^{(1)}$ [Eq. (S.3.20)] as an infinite series of multipoles with respect to $\boldsymbol{r} = \boldsymbol{r}' + \boldsymbol{\xi}$:

$$\begin{aligned}\zeta^{(1)} &= -\tfrac{1}{2}a^4\boldsymbol{K}_{\boldsymbol{\xi}}^{\mathrm{ext}} : \frac{(\boldsymbol{r}-\boldsymbol{\xi})(\boldsymbol{r}-\boldsymbol{\xi})}{|\boldsymbol{r}-\boldsymbol{\xi}|^4} - \tfrac{1}{6}a^6(\boldsymbol{\nabla}\boldsymbol{K}^{\mathrm{ext}})_{\boldsymbol{\xi}}(\cdot)^3\frac{(\boldsymbol{r}-\boldsymbol{\xi})(\boldsymbol{r}-\boldsymbol{\xi})(\boldsymbol{r}-\boldsymbol{\xi})}{|\boldsymbol{r}-\boldsymbol{\xi}|^6} \\ &= -\tfrac{1}{2}a^4\boldsymbol{K}_{\boldsymbol{\xi}}^{\mathrm{ext}} : \sum_{n=0}^{\infty}\frac{(-\boldsymbol{\xi}\cdot\boldsymbol{\nabla})^n}{n!}\left(\frac{\boldsymbol{r}\boldsymbol{r}}{r^4}\right) - \tfrac{1}{6}a^6(\boldsymbol{\nabla}\boldsymbol{K}^{\mathrm{ext}})_{\boldsymbol{\xi}}(\cdot)^3\sum_{n=0}^{\infty}\frac{(-\boldsymbol{\xi}\cdot\boldsymbol{\nabla})^n}{n!}\left(\frac{\boldsymbol{r}\boldsymbol{r}\boldsymbol{r}}{r^6}\right). \qquad \text{(S.3.29)}\end{aligned}$$

The unique solution that satisfies (S.3.27)-(S.3.29) is

$$\begin{aligned}\zeta_{\mathcal{U}}^{(2)} = -\zeta_{\mathcal{P}}^{(2)} &= \tfrac{1}{2}a^4\boldsymbol{K}_{\boldsymbol{\xi}}^{\mathrm{ext}} : \sum_{n=0}^{\infty}\left(\frac{r}{R}\right)^{2(n+2)}\frac{(-\boldsymbol{\xi}\cdot\boldsymbol{\nabla})^n}{n!}\left(\frac{\boldsymbol{r}\boldsymbol{r}}{r^4}\right) + \tfrac{1}{6}a^6(\boldsymbol{\nabla}\boldsymbol{K}^{\mathrm{ext}})_{\boldsymbol{\xi}}(\cdot)^3\sum_{n=0}^{\infty}\left(\frac{r}{R}\right)^{2(n+3)}\frac{(-\boldsymbol{\xi}\cdot\boldsymbol{\nabla})^n}{n!}\left(\frac{\boldsymbol{r}\boldsymbol{r}\boldsymbol{r}}{r^6}\right) \\ &= \tfrac{1}{2}a^4\boldsymbol{K}_{\boldsymbol{\xi}}^{\mathrm{ext}} \cdot \left(\frac{\boldsymbol{r}\boldsymbol{r}}{R^4} + \frac{4\boldsymbol{r}\boldsymbol{r}(\boldsymbol{r}\cdot\boldsymbol{\xi}) - 2r^2\boldsymbol{r}\boldsymbol{\xi}}{R^6} + \cdots\right) + \tfrac{1}{6}a^6(\boldsymbol{\nabla}\boldsymbol{K}^{\mathrm{ext}})_{\boldsymbol{\xi}}(\cdot)^3\left(\frac{\boldsymbol{r}\boldsymbol{r}\boldsymbol{r}}{R^6} + \cdots\right). \qquad \text{(S.3.30)}\end{aligned}$$

It is unnecessary to keep all terms in the infinite series, since higher reflections will contribute like-ordered corrections. The terms retained in Eq. (S.3.30) satisfy the wetting condition at $r = R$ up to $O(a^4/R^4)$ errors.

As in the electric problem, the second reflection introduces a negligible correction to the force:

$$\begin{aligned}
\boldsymbol{F}^{(02)} &= -\gamma \oint_{r'=a} \hat{\boldsymbol{n}} \cdot \left[\nabla\zeta^{(0)}\nabla\zeta^{(2)} + \nabla\zeta^{(2)}\nabla\zeta^{(0)} - (\nabla\zeta^{(0)} \cdot \nabla\zeta^{(2)})\boldsymbol{\delta}\right] \mathrm{d}s \\
&= \mathbf{0}, && \text{(S.3.31)} \\
\boldsymbol{F}^{(12)} &= -\gamma \oint_{r'=a} \hat{\boldsymbol{n}} \cdot \left[\nabla\zeta^{(1)}\nabla\zeta^{(2)} + \nabla\zeta^{(2)}\nabla\zeta^{(1)} - (\nabla\zeta^{(1)} \cdot \nabla\zeta^{(2)})\boldsymbol{\delta}\right] \mathrm{d}s \\
&\simeq \mathbf{0}, && \text{(S.3.32)} \\
\boldsymbol{F}^{(22)} &= -\gamma \oint_{r'=a} \hat{\boldsymbol{n}} \cdot \left(\nabla\zeta^{(2)}\nabla\zeta^{(2)} - \tfrac{1}{2}|\nabla\zeta^{(2)}|^2\boldsymbol{\delta}\right) \mathrm{d}s \\
&= \mathbf{0}. && \text{(S.3.33)}
\end{aligned}$$

The only finite contribution to the energy is due to $W^{(02)}$:

$$\begin{aligned}
W^{(02)}_{\mathcal{U}} &= -\gamma \oint_{r'=a} \zeta^{(0)}(\hat{\boldsymbol{n}} \cdot \nabla\zeta^{(2)}_{\mathcal{U}})\mathrm{d}s - \gamma \oint_{r=R} \zeta^{(0)}(\hat{\boldsymbol{n}} \cdot \nabla\zeta^{(2)}_{\mathcal{U}})\,\mathrm{d}s \\
&\simeq \tfrac{1}{4}\pi\gamma a^4 \left[|\boldsymbol{K}^{\mathrm{ext}}_{\boldsymbol{\xi}}|^2 + \tfrac{1}{12}a^2|(\nabla\boldsymbol{K}^{\mathrm{ext}})_{\boldsymbol{\xi}}|^2\right], && \text{(S.3.34a)}
\end{aligned}$$

for the fixed-hieght case, and

$$\begin{aligned}
W^{(02)}_{\mathcal{P}} &= -\gamma \oint_{r'=a} \zeta^{(0)}(\hat{\boldsymbol{n}} \cdot \nabla\zeta^{(2)}_{\mathcal{P}})\mathrm{d}s - \gamma \oint_{r=R} \zeta^{(0)}(\hat{\boldsymbol{n}} \cdot \nabla\zeta^{(2)}_{\mathcal{P}})\,\mathrm{d}s \\
&\simeq -\tfrac{1}{4}\pi\gamma a^4 \left[|\boldsymbol{K}^{\mathrm{ext}}_{\boldsymbol{\xi}}|^2 + \tfrac{1}{12}a^2|(\nabla\boldsymbol{K}^{\mathrm{ext}})_{\boldsymbol{\xi}}|^2\right], && \text{(S.3.34b)}
\end{aligned}$$

for the fixed-slope case. The other terms are negligible to the present order of approximation:

$$\begin{aligned}
W^{(12)} &= -\tfrac{1}{2}\gamma \oint_{r'=a} \left[\zeta^{(1)}(\hat{\boldsymbol{n}} \cdot \nabla\zeta^{(2)}) + \zeta^{(2)}(\hat{\boldsymbol{n}} \cdot \nabla\zeta^{(1)})\right] \mathrm{d}s - \tfrac{1}{2}\gamma \oint_{r=R} \left[\zeta^{(1)}(\hat{\boldsymbol{n}} \cdot \nabla\zeta^{(2)}) + \zeta^{(2)}(\hat{\boldsymbol{n}} \cdot \nabla\zeta^{(1)})\right] \mathrm{d}s \\
&= 0, && \text{(S.3.35)} \\
W^{(22)} &= -\tfrac{1}{2}\gamma \oint_{r'=a} \zeta^{(2)}(\hat{\boldsymbol{n}} \cdot \nabla\zeta^{(2)})\mathrm{d}s - \tfrac{1}{2}\gamma \oint_{r=R} \zeta^{(2)}(\hat{\boldsymbol{n}} \cdot \nabla\zeta^{(2)})\,\mathrm{d}s \\
&\simeq 0. && \text{(S.3.36)}
\end{aligned}$$

In the last equation, errors of $O(a^4/R^4)$ have been neglected.

S.3.1.4 Composite solution

Summing Eqs. (S.3.5), (S.3.20), and (S.3.30) yields the following solution for the interface height:

$$\begin{aligned}
\zeta_{\mathcal{U}} = {} & \underbrace{\tfrac{1}{2}\boldsymbol{K}^{\mathrm{ext}}_{\mathbf{0}} : \boldsymbol{rr} + \tfrac{1}{6}(\nabla\boldsymbol{K}^{\mathrm{ext}})_{\mathbf{0}}\,(\cdot)^3\,\boldsymbol{rrr}}_{\zeta^{(0)}} \underbrace{- \tfrac{1}{2}a^4\boldsymbol{K}^{\mathrm{ext}}_{\boldsymbol{\xi}} : \frac{\boldsymbol{r}'\boldsymbol{r}'}{r'^4} - \tfrac{1}{6}a^6(\nabla\boldsymbol{K}^{\mathrm{ext}})_{\boldsymbol{\xi}}(\cdot)^3\frac{\boldsymbol{r}'\boldsymbol{r}'\boldsymbol{r}'}{r'^6}}_{\zeta^{(1)}} \\
& \underbrace{+ \tfrac{1}{2}a^4\boldsymbol{K}^{\mathrm{ext}}_{\boldsymbol{\xi}} \cdot \left(\frac{\boldsymbol{rr}}{R^4} + \frac{4\boldsymbol{rr}(\boldsymbol{r}\cdot\boldsymbol{\xi}) - 2r^2\boldsymbol{r\xi}}{R^6} + \cdots\right) + \tfrac{1}{6}a^6(\nabla\boldsymbol{K}^{\mathrm{ext}})_{\boldsymbol{\xi}}(\cdot)^3\left(\frac{\boldsymbol{rrr}}{R^6} + \cdots\right)}_{\zeta^{(2)}_{\mathcal{U}}} + \cdots, && \text{(S.3.37)}
\end{aligned}$$

for fixed heights, or

$$\zeta_{\mathcal{V}} = \underbrace{\tfrac{1}{2}\boldsymbol{K}_{\mathbf{0}}^{\text{ext}} : \boldsymbol{r}\boldsymbol{r} + \tfrac{1}{6}(\nabla \boldsymbol{K}^{\text{ext}})_{\mathbf{0}}\,(\cdot)^3\,\boldsymbol{r}\boldsymbol{r}\boldsymbol{r}}_{\zeta^{(0)}}\ \underbrace{-\tfrac{1}{2}a^4\boldsymbol{K}_{\boldsymbol{\xi}}^{\text{ext}} : \frac{\boldsymbol{r}'\boldsymbol{r}'}{r'^4} - \tfrac{1}{6}a^6(\nabla \boldsymbol{K}^{\text{ext}})_{\boldsymbol{\xi}}(\cdot)^3\frac{\boldsymbol{r}'\boldsymbol{r}'\boldsymbol{r}'}{r'^6}}_{\zeta^{(1)}}$$

$$\underbrace{-\tfrac{1}{2}a^4\boldsymbol{K}_{\boldsymbol{\xi}}^{\text{ext}} \cdot \left(\frac{\boldsymbol{r}\boldsymbol{r}}{R^4} + \frac{4\boldsymbol{r}\boldsymbol{r}(\boldsymbol{r}\cdot\boldsymbol{\xi}) - 2r^2\boldsymbol{r}\boldsymbol{\xi}}{R^6} + \cdots\right) - \tfrac{1}{6}a^6(\nabla \boldsymbol{K}^{\text{ext}})_{\boldsymbol{\xi}}(\cdot)^3\left(\frac{\boldsymbol{r}\boldsymbol{r}\boldsymbol{r}}{R^6} + \cdots\right)}_{\zeta_{\mathcal{P}}^{(2)}} + \cdots, \tag{S.3.38}$$

for fixed slopes. Recombining terms in Eqs. (S.3.37)-(S.3.38) yields (4.14)-(4.15) in the main text. A sketch of each reflection is shown in Fig. 9.

The sum of Eqs. (S.3.9), (S.3.23)-(S.3.24), and (S.3.31)-(S.3.33) gives the total capillary force

$$\boldsymbol{F} = \underbrace{-\tfrac{1}{4}\pi\gamma a^4(\nabla|\boldsymbol{K}^{\text{ext}}|^2)_{\boldsymbol{\xi}}}_{\boldsymbol{F}^{(01)}} + \cdots, \tag{S.3.39}$$

where we have used $2\boldsymbol{K}_{\boldsymbol{\xi}}^{\text{ext}} : (\nabla \boldsymbol{K}^{\text{ext}})_{\boldsymbol{\xi}} = (\nabla|\boldsymbol{K}^{\text{ext}}|^2)_{\boldsymbol{\xi}}$. The leading term in Eq. (S.3.39) is equivalent to (4.17) in the main text. As in the electric problem, the dominant contribution is due to $\boldsymbol{F}^{(01)}$. Similarly, after summing Eqs. (S.3.12), (S.3.25)-(S.3.26), and (S.3.34)-(S.3.36), we obtain the energy

$$W_{\mathcal{U}} = -W_{\mathcal{P}} = \underbrace{\tfrac{1}{4}\pi\gamma a^4\left[|\boldsymbol{K}_{\boldsymbol{\xi}}^{\text{ext}}|^2 + \tfrac{1}{12}a^2|(\nabla \boldsymbol{K}^{\text{ext}})_{\boldsymbol{\xi}}|^2\right]}_{W_{\mathcal{U}}^{(02)} \text{ or } -W_{\mathcal{P}}^{(02)}} + \cdots, \tag{S.3.40}$$

which is equivalent to Eqs. (4.18) and (4.21) in the main text.

S.3.1.5 Higher reflections: finite-size effects

In §S.1.1.5, we computed the $O(a^2/R^2)$ corrections to the electric force and energy due to higher reflections. The magnitude of these corrections are characteristic of dipolar interactions, which decay like $1/r'$ from the center of the particle. For quadrupolar interactions, the rate of decay goes like $1/r'^2$ and so the first correction to the capillary force and energy are of $O(a^4/R^4)$. Computing this correction is straightforward for the capillary force, which only requires integrals over the particle boundary. For the capillary energy, which includes integrals over the shell boundary, a large number of terms must be retained in the approximation for the interface height in order to acquire all $O(a^4/R^4)$ integral contributions. This very quickly becomes a tedious endeavor. A more practical approach is the one used to derive Eqs. (4.18) and (4.21) in the main text – that is, apply the wetting condition (4.9) to eliminate the integral over $r = R$ in Eq. (S.3.10). This allows us to compute the energy solely in terms of integrals over the particle boundary.

Without going into the details of the derivation and instead focusing on the results, we expand Eqs. (S.3.39) and (S.3.40) up to the $O(a^4/R^4)$ correction. For fixed heights on the outer shell, we obtain

$$\boldsymbol{F}_{\mathcal{U}} = -\frac{\delta W_{\mathcal{U}}}{\delta\boldsymbol{\xi}} = -\tfrac{1}{4}\pi\gamma a^4\left(1 + \frac{a^4}{R^4}\right)(\nabla|\boldsymbol{K}^{\text{ext}}|^2)_{\boldsymbol{\xi}} + \cdots \tag{S.3.41}$$

and

$$W_{\mathcal{U}} = \tfrac{1}{4}\pi\gamma a^4\left[\left(1 + \frac{a^4}{R^4}\right)|\boldsymbol{K}_{\boldsymbol{\xi}}^{\text{ext}}|^2 + \tfrac{1}{12}a^2|(\nabla \boldsymbol{K}^{\text{ext}})_{\boldsymbol{\xi}}|^2\right] + \cdots, \tag{S.3.42}$$

where the terms neglected are of $O(a^8/R^8)$. For fixed slopes, we obtain instead

$$\boldsymbol{F}_{\mathcal{P}} = \frac{\delta W_{\mathcal{P}}}{\delta\boldsymbol{\xi}} = -\tfrac{1}{4}\pi\gamma a^4\left(1 - \frac{a^4}{R^4}\right)(\nabla|\boldsymbol{K}^{\text{ext}}|^2)_{\boldsymbol{\xi}} + \cdots \tag{S.3.43}$$

and

$$W_{\mathcal{P}} = -\tfrac{1}{4}\pi\gamma a^4\left[\left(1 - \frac{a^4}{R^4}\right)|\boldsymbol{K}_{\boldsymbol{\xi}}^{\text{ext}}|^2 + \tfrac{1}{12}a^2|(\nabla \boldsymbol{K}^{\text{ext}})_{\boldsymbol{\xi}}|^2\right] + \cdots. \tag{S.3.44}$$

Note that the force now depends upon the wetting condition applied at the shell boundary through the $O(a^4/R^4)$ finite-size correction. A fixed-height condition repels particles with symmetrically pinned contact lines, whereas a fixed-slope condition attracts them.

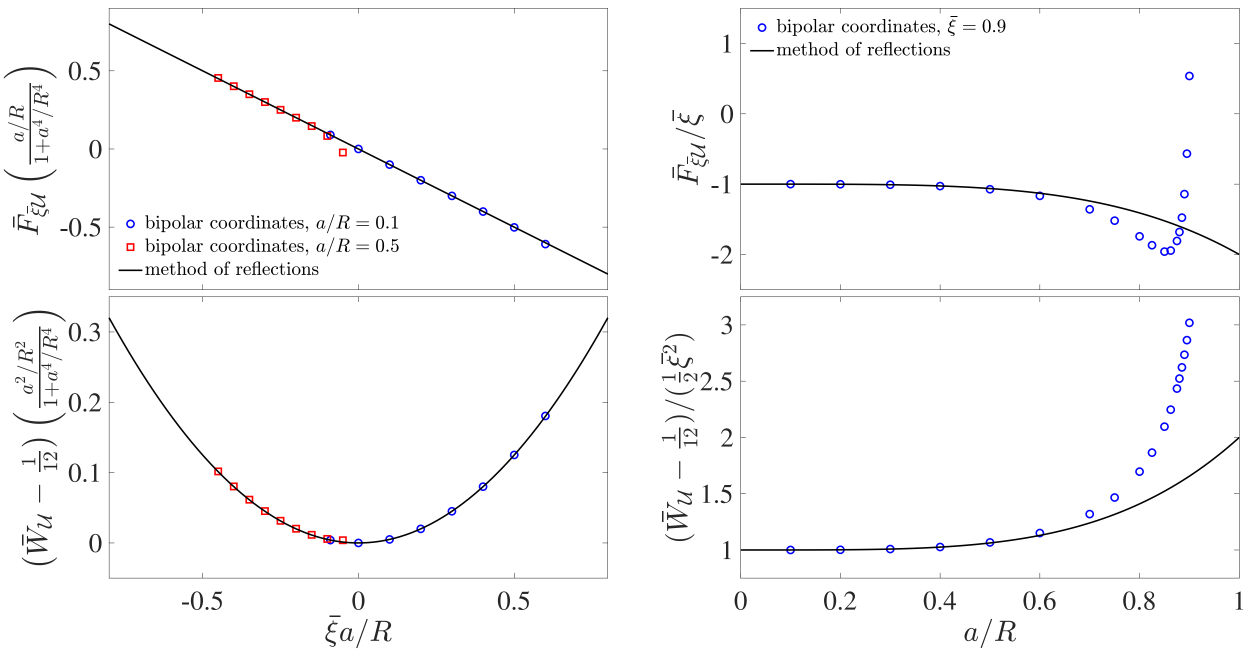

Figure S.5 Capillary force and energy for a particle with a symmetrically pinned contact line trapped at an interface with a fixed height distribution on the outer shell. The dimensionless force $\bar{\boldsymbol{F}} = \boldsymbol{F}/[\frac{1}{4}\pi\gamma a^5|(\boldsymbol{\nabla}\boldsymbol{K}^{\text{ext}})_{\mathbf{0}}|^2]$ (*top*) and energy $\bar{W} = W/[\frac{1}{4}\pi\gamma a^6|(\boldsymbol{\nabla}\boldsymbol{K}^{\text{ext}})_{\mathbf{0}}|^2]$ (*bottom*) are plotted against the relative particle position $\bar{\boldsymbol{\xi}} = (\boldsymbol{\xi} - \boldsymbol{\xi}^*)/a$ (*left*) and the radius ratio a/R (*right*), where $\boldsymbol{\xi}^*$ is the fixed point as defined by Eq. (4.25) in the main text. For the numerical calculations in bipolar coordinates, we set the curvature amplitudes $K_0 = K_1 = 1$ and phase angles $\alpha_0 = \alpha_1 = 0$ [cf. Eqs. (S.4.37)-(S.4.38)].

Equations (S.3.41)-(S.3.42) and (S.3.43)-(S.3.44) are plotted in Figs. S.5 and S.6, respectively, against the radius ratio a/R and particle position $\boldsymbol{\xi}$. Also plotted are exact results using eigenfunction expansions in bipolar coordinates (see §S.4). Although the two solutions agree very well when a/R is small, qualitative differences are observed as $a/R \to 1$ when the outer height distribution is fixed. Whereas the reflections solution (S.3.41) predicts a negative finite-size correction of $O(a^4/R^4)$, the bipolar solution indicates that this correction switches sign (from negative to positive) as the particle fills the shell (see Fig. S.5, *right*). The same effect is not observed when the outer slope distribution is fixed (Fig. S.6). Further exploration of this finite-size effect is beyond the scope of the present study.

To conclude this section, it is worth discussing finite-size corrections to the external work W^{ext} supplied to the system, which has not been mentioned up to this point. As was indicated near the beginning of this section, the results (S.3.42) and (S.3.44) for the energy W can be derived without having to evaluate integrals over the shell boundary, if one is clever about applying the wetting condition (4.9) *before* integration. This same trick cannot be applied to the Young-Dupré work of adhesion [Eq. (4.23) in the main text], which is defined solely as an integral over the shell boundary. This implies that, to compute the $O(a^4/R^4)$ correction to Eq. (4.23) (not presented here), one has to expand ζ up to a large number of terms. Fortunately, if there are no force sources in the domain, then the work of adhesion is always = 0 for fixed heights on the outer shell and = $2W_{\mathcal{P}}$ for fixed slopes, regardless of the value of a/R. This means that we need not compute the adhesion energy directly for particles with symmetrically pinned contact lines. On the other hand, consider a particle with an *undulated* contact line (discussed in Appendix E of the main text) trapped at an interface with a fixed distribution of slopes on the outer shell. In this case, the work of adhesion $\neq 2W_{\mathcal{P}}$ because the contact-line undulation acts as a source of force density. Thus, one is again faced with the problem of directly evaluating the adhesion energy through integrals over the shell boundary [cf. Eq. (E.5) in Appendix E]. Exactly the same problem occurs in electrostatics if a permanent electret is suspended in a dielectric medium and a field is applied through a fixed distribution of potentials.

S.3.2 Particle with an equilibrium contact angle

Particles with equilibrium contact angles are discussed in §4.2 in the main text. In keeping with the structure of that section, we divide our attention between cylindrical particles with 90° contact angles (§S.3.2.1) and spherical particles with unrestricted

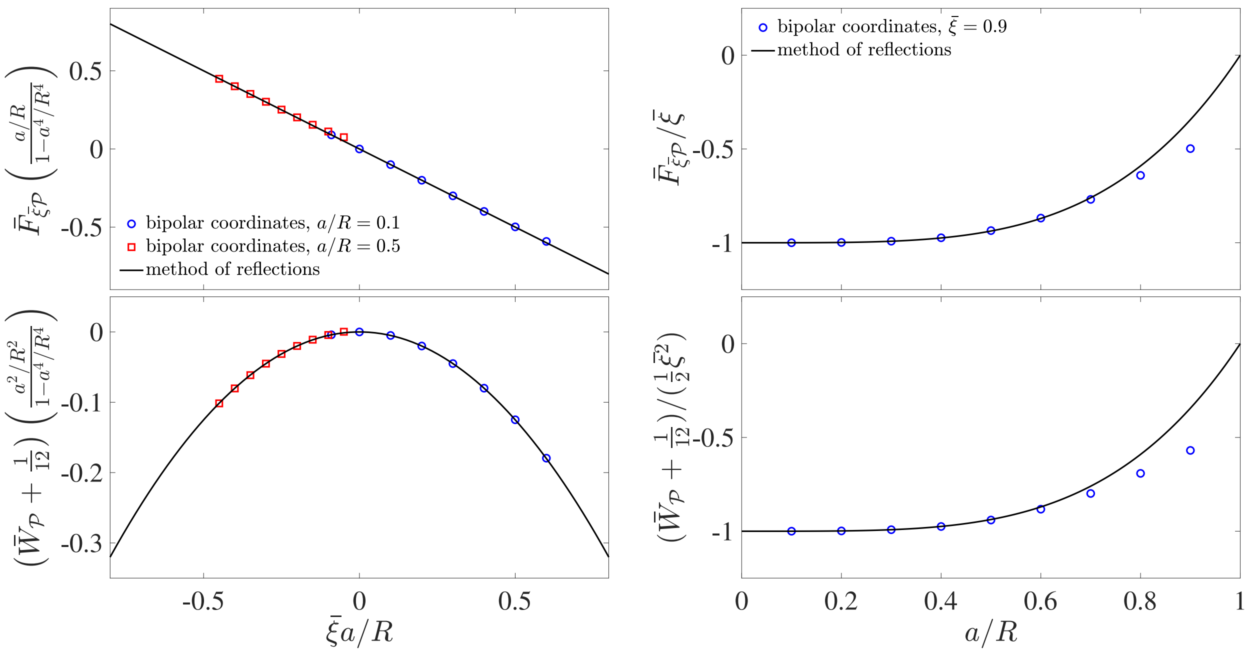

Figure S.6 Capillary force and energy for a particle with a symmetrically pinned contact line trapped at an interface with a fixed outer slope distribution. The variables have the same meaning as in Fig. S.5.

contact angles (§S.3.2.2).

S.3.2.1 Cylindrical particle

For cylindrical particles with 90° contact angles, the Dirichlet condition (4.10) is replaced by the Neumann condition

$$\hat{\boldsymbol{n}} \cdot \boldsymbol{\nabla}\zeta = \hat{\boldsymbol{n}} \cdot \boldsymbol{\Omega}_\times \quad \text{at} \quad r' = a. \tag{4.26}$$

The reflections are computed in the same way as in §S.3.1 except that the particle reflections reverse sign, giving $-(\zeta^{(1)} + \zeta^{(2)})$, $-(\zeta^{(5)} + \zeta^{(6)})$, etc. For more details, the reader is referred to §S.1.2 on insulating particles.

Below, we present the analogues of Eqs. (S.3.41)-(S.3.44) for cylindrical particles with equilibrium contact angles:

$$\boldsymbol{F}_{\mathcal{U}} = -\frac{\delta W_{\mathcal{U}}}{\delta \boldsymbol{\xi}} = \tfrac{1}{4}\pi\gamma a^4 \left(1 - \frac{a^4}{R^4}\right) (\boldsymbol{\nabla}|\boldsymbol{K}^{\text{ext}}|^2)_{\boldsymbol{\xi}} + \cdots, \tag{S.3.45}$$

$$W_{\mathcal{U}} = -\tfrac{1}{4}\pi\gamma a^4 \left[\left(1 - \frac{a^4}{R^4}\right) |\boldsymbol{K}^{\text{ext}}_{\boldsymbol{\xi}}|^2 + \tfrac{1}{12}a^2|(\boldsymbol{\nabla}\boldsymbol{K}^{\text{ext}})_{\boldsymbol{\xi}}|^2\right] + \cdots, \tag{S.3.46}$$

for fixed heights, and

$$\boldsymbol{F}_{\mathcal{P}} = \frac{\delta W_{\mathcal{P}}}{\delta \boldsymbol{\xi}} = \tfrac{1}{4}\pi\gamma a^4 \left(1 + \frac{a^4}{R^4}\right) (\boldsymbol{\nabla}|\boldsymbol{K}^{\text{ext}}|^2)_{\boldsymbol{\xi}} + \cdots, \tag{S.3.47}$$

$$W_{\mathcal{P}} = \tfrac{1}{4}\pi\gamma a^4 \left[\left(1 + \frac{a^4}{R^4}\right) |\boldsymbol{K}^{\text{ext}}_{\boldsymbol{\xi}}|^2 + \tfrac{1}{12}a^2|(\boldsymbol{\nabla}\boldsymbol{K}^{\text{ext}})_{\boldsymbol{\xi}}|^2\right] + \cdots. \tag{S.3.48}$$

for fixed slopes. As before, the neglected terms are of $O(a^8/R^8)$. The leading-order terms can be found in Eqs. (4.28)-(4.29) in the main text. Equations (S.3.45)-(S.3.48) are plotted in Figs. S.7-(S.8), which may be compared to Figs. S.5-S.6.

S.3.2.2 Spherical particle

For spherical particles with arbitrary contact angles, the correct boundary condition is

$$\hat{\boldsymbol{n}} \cdot \boldsymbol{\nabla}\zeta - \frac{\zeta}{r'} = -\frac{U}{r'} \quad \text{at} \quad r' = a. \tag{4.30}$$

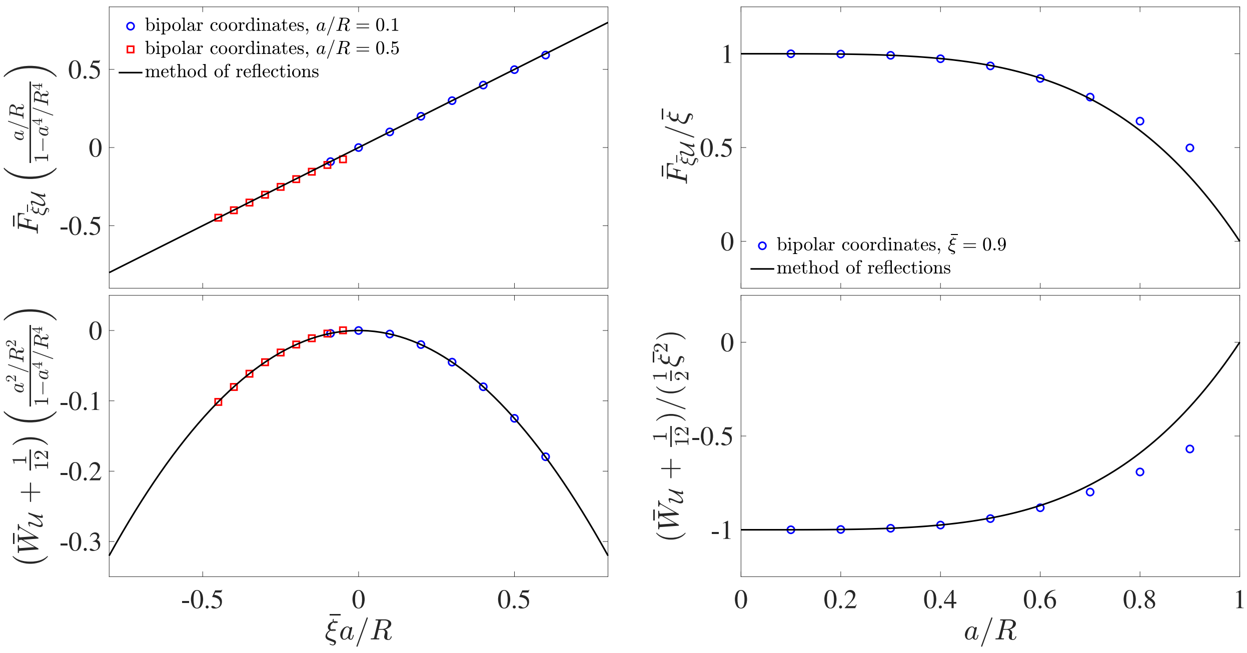

Figure S.7 Capillary force and energy for a cylindrical particle with an equilibrium contact angle of 90° trapped at an interface with a fixed outer height distribution. The variables have the same meaning as in Fig. S.5.

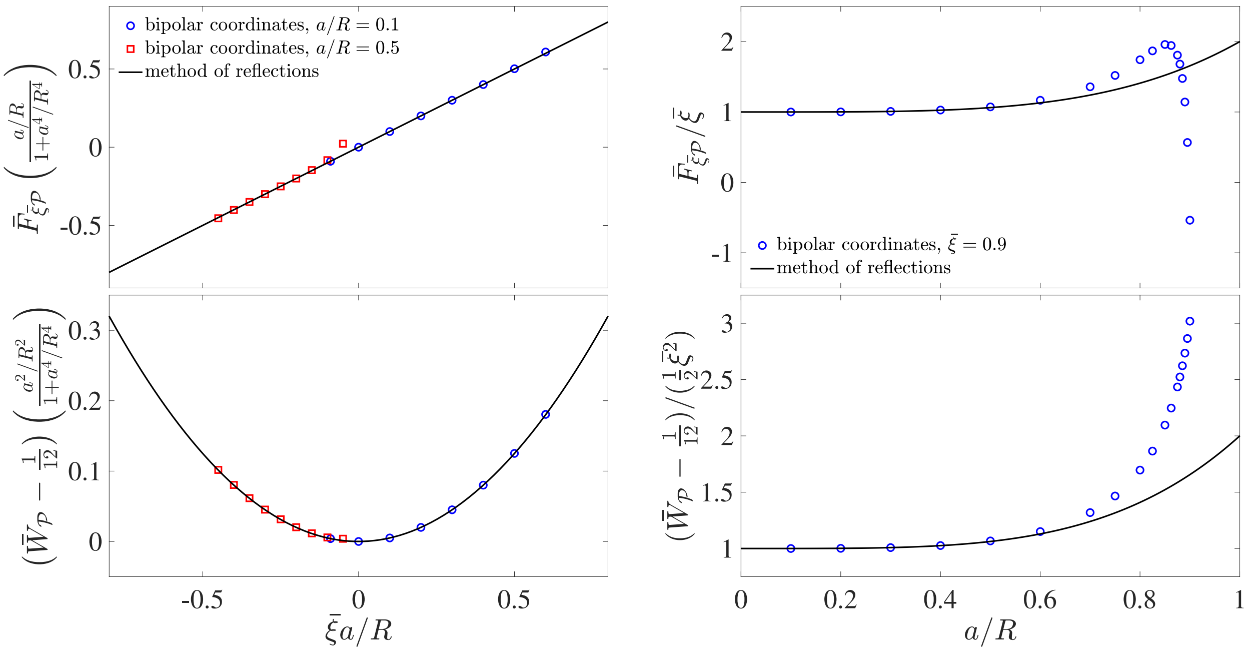

Figure S.8 Capillary force and energy for a cylindrical particle with an equilibrium contact angle of 90° trapped at an interface with a fixed outer slope distribution. The variables have the same meaning as in Fig. S.5.

Compared to the Neumann condition (4.26) for cylinders, the Robin boundary condition (4.30) for spheres alters the numerical prefactors of the particle reflections. Each particle reflection introduces an extra prefactor of $\frac{1}{3}$ for terms multiplied by $\boldsymbol{K}_{\boldsymbol{\xi}}^{\text{ext}}$ and a factor of $\frac{1}{2}$ for those multiplied by $(\boldsymbol{\nabla K}^{\text{ext}})_{\boldsymbol{\xi}}$. The resulting expression for the interface height around a spherical particle with an arbitrary contact angle is

$$\zeta_{\mathcal{U}} = \underbrace{\tfrac{1}{2}\boldsymbol{K}_{\boldsymbol{0}}^{\text{ext}} : \boldsymbol{rr} + \tfrac{1}{6}(\boldsymbol{\nabla K}^{\text{ext}})_{\boldsymbol{0}}\,(\cdot)^3\,\boldsymbol{rrr}}_{\zeta^{(0)}} + \underbrace{\tfrac{1}{6}a^4\boldsymbol{K}_{\boldsymbol{\xi}}^{\text{ext}} : \frac{\boldsymbol{r'r'}}{r'^4} + \tfrac{1}{12}a^6(\boldsymbol{\nabla K}^{\text{ext}})_{\boldsymbol{\xi}}(\cdot)^3\frac{\boldsymbol{r'r'r'}}{r'^6}}_{\zeta^{(1)}}$$
$$\underbrace{-\tfrac{1}{6}a^4\boldsymbol{K}_{\boldsymbol{\xi}}^{\text{ext}}\cdot\left(\frac{\boldsymbol{rr}}{R^4} + \frac{4\boldsymbol{rr}(\boldsymbol{r}\cdot\boldsymbol{\xi}) - 2r^2\boldsymbol{r\xi}}{R^6} + \cdots\right) - \tfrac{1}{12}a^6(\boldsymbol{\nabla K}^{\text{ext}})_{\boldsymbol{\xi}}(\cdot)^3\left(\frac{\boldsymbol{rrr}}{R^6} + \cdots\right)}_{\zeta_{\mathcal{U}}^{(2)}} + \cdots, \tag{S.3.49}$$

for fixed outer heights, or

$$\zeta_{\mathcal{V}} = \underbrace{\tfrac{1}{2}\boldsymbol{K}_{\boldsymbol{0}}^{\text{ext}} : \boldsymbol{rr} + \tfrac{1}{6}(\boldsymbol{\nabla K}^{\text{ext}})_{\boldsymbol{0}}\,(\cdot)^3\,\boldsymbol{rrr}}_{\zeta^{(0)}} + \underbrace{\tfrac{1}{6}a^4\boldsymbol{K}_{\boldsymbol{\xi}}^{\text{ext}} : \frac{\boldsymbol{r'r'}}{r'^4} + \tfrac{1}{12}a^6(\boldsymbol{\nabla K}^{\text{ext}})_{\boldsymbol{\xi}}(\cdot)^3\frac{\boldsymbol{r'r'r'}}{r'^6}}_{\zeta^{(1)}}$$
$$\underbrace{+\tfrac{1}{6}a^4\boldsymbol{K}_{\boldsymbol{\xi}}^{\text{ext}}\cdot\left(\frac{\boldsymbol{rr}}{R^4} + \frac{4\boldsymbol{rr}(\boldsymbol{r}\cdot\boldsymbol{\xi}) - 2r^2\boldsymbol{r\xi}}{R^6} + \cdots\right) + \tfrac{1}{12}a^6(\boldsymbol{\nabla K}^{\text{ext}})_{\boldsymbol{\xi}}(\cdot)^3\left(\frac{\boldsymbol{rrr}}{R^6} + \cdots\right)}_{\zeta_{\mathcal{P}}^{(2)}} + \cdots, \tag{S.3.50}$$

for fixed outer slopes. This pattern continues to higher reflections. Another important difference between cylinders and spheres is that the contact line is, in the latter case, non-circular. Thus, additional terms must be added to the energy to account for the contact-line distortion, as discussed in §4.2.2 in the main text.

For the energy calculation, we must account for the distortion of the contact line needed to maintain a fixed contact angle α on a spherical particle. We denote by W_1 the energy given by Eq. (S.3.10), which accounts for interfacial distortions outside the particle. The additional work W_2 done to distort the contact line is

$$W_2 = \tfrac{1}{2}\gamma \oint_{r'=a} \frac{\tilde{\zeta}^2}{a}\,\mathrm{d}s, \tag{S.3.51}$$

where $\tilde{\zeta} = (\zeta - U - \boldsymbol{\Omega}_\times \cdot \boldsymbol{r}')|_{r'=a}$ is the height of the contact line in the co-translated and co-rotated frame [see also Eq. (4.36) in the main text]. The expression (S.3.51) for W_2 may be expanded in terms of the reflections $\zeta^{(n)}$, $U^{(n)}$, and $\Omega^{(n)}$, yielding a double series akin to (S.3.11) for W_1. The total energy is then given by the sum $W = W_1 + W_2$. The calculation is straightforward, and, for the sake of brevity, will not be delineated in any further detail here. Below, we present the final results for the force and energy.

Up to the $O(a^4/R^4)$ correction, the force and energy for spherical particles with equilibrium contact angles are, respectively,

$$\boldsymbol{F}_{\mathcal{U}} = -\frac{\delta W_{\mathcal{U}}}{\delta\boldsymbol{\xi}} = \tfrac{1}{12}\pi\gamma a^4\left(1 - \frac{1}{3}\frac{a^4}{R^4}\right)(\boldsymbol{\nabla}|\boldsymbol{K}^{\text{ext}}|^2)_{\boldsymbol{\xi}} + \cdots \tag{S.3.52}$$

and

$$W_{\mathcal{U}} = -\tfrac{1}{12}\pi\gamma a^4\left[\left(1 - \frac{1}{3}\frac{a^4}{R^4}\right)|\boldsymbol{K}_{\boldsymbol{\xi}}^{\text{ext}}|^2 + \tfrac{1}{8}a^2|(\boldsymbol{\nabla K}^{\text{ext}})_{\boldsymbol{\xi}}|^2\right] + \cdots, \tag{S.3.53}$$

assuming a fixed distribution of heights on the outer shell. For fixed slopes, we obtain instead

$$\boldsymbol{F}_{\mathcal{P}} = \frac{\delta W_{\mathcal{P}}}{\delta\boldsymbol{\xi}} = \tfrac{1}{12}\pi\gamma a^4\left(1 + \frac{1}{3}\frac{a^4}{R^4}\right)(\boldsymbol{\nabla}|\boldsymbol{K}^{\text{ext}}|^2)_{\boldsymbol{\xi}} + \cdots \tag{S.3.54}$$

and

$$W_{\mathcal{P}} = \tfrac{1}{12}\pi\gamma a^4\left[\left(1 + \frac{1}{3}\frac{a^4}{R^4}\right)|\boldsymbol{K}_{\boldsymbol{\xi}}^{\text{ext}}|^2 + \tfrac{1}{8}a^2|(\boldsymbol{\nabla K}^{\text{ext}})_{\boldsymbol{\xi}}|^2\right] + \cdots. \tag{S.3.55}$$

Equations (S.3.52)-(S.3.55) for spheres may be compared to (S.3.45)-(S.3.48) for cylinders. The leading-order terms appear in Eqs. (4.32) and (4.37) in the main text. Figures S.9-S.10 plot the reflections expansions (S.3.52)-(S.3.55) alongside the corresponding exact solution in bipolar coordinates. Compared to the cylinder solution (Figs. S.7-S.8), the reflections expansions for spheres generally show better agreement with the bipolar solution. Details of the bipolar solution method are given in the following section.

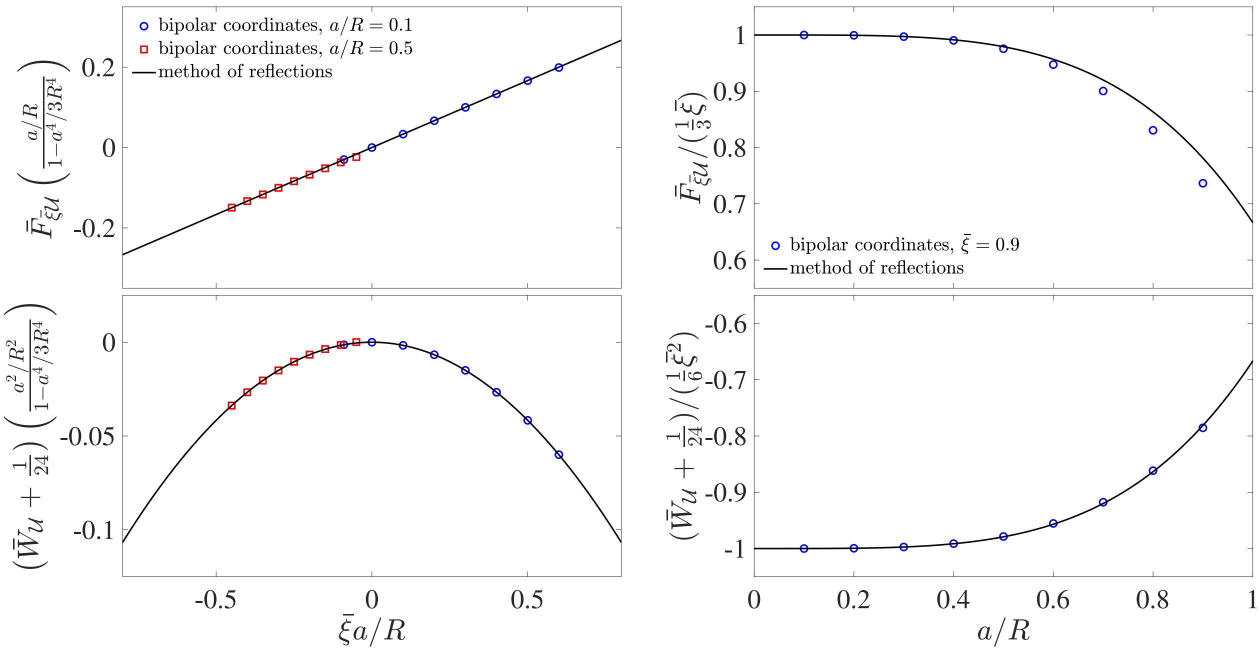

Figure S.9 Capillary force and energy for a spherical particle with an equilibrium contact angle trapped at an interface with a fixed outer height distribution. The variables have the same meaning as in Fig. S.5.

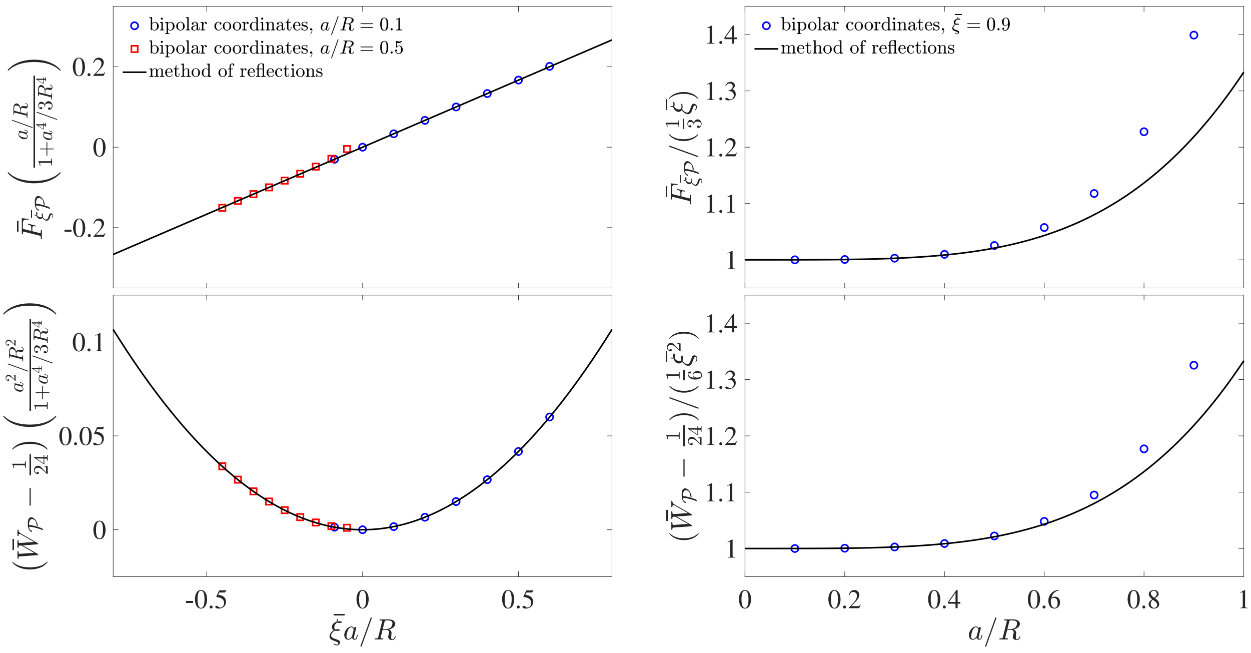

Figure S.10 Capillary force and energy for a spherical particle with an equilibrium contact angle trapped at an interface with a fixed outer slope distribution. The variables have the same meaning as in Fig. S.5.

S.4 Capillary problem: bipolar coordinates

In §S.2, we developed an exact solution to the electric problem using bipolar coordinates. We apply the same method, below, for the capillary problem.

S.4.1 Governing equations

For details on the transformation from Cartesian coordinates (x, y) to bipolar coordinates (σ, τ), see Appendix S.B at the end of this supplemental document. In bipolar coordinates, the Laplace problem (4.8)-(4.12) for particles with pinned contact lines becomes

$$\frac{\partial^2\zeta}{\partial\sigma^2} + \frac{\partial^2\zeta}{\partial\tau^2} = 0 \quad \text{for} \quad \tau_R \le \tau \le \tau_a, \tag{S.4.1}$$

with

either
$$\zeta_{\mathcal{U}} = \zeta^{\text{ext}} \quad \text{at} \quad \tau = \tau_R \tag{S.4.2a}$$

or
$$\frac{\partial\zeta_{\mathcal{P}}}{\partial\tau} = \frac{\partial\zeta^{\text{ext}}}{\partial\tau} \quad \text{at} \quad \tau = \tau_R, \tag{S.4.2b}$$

and

$$\frac{\partial\zeta}{\partial\sigma} = \frac{c(\hat{\boldsymbol{\sigma}} \cdot \boldsymbol{\Omega}_\times)}{\cosh\tau - \cos\sigma} \quad \text{at} \quad \tau = \tau_a, \tag{S.4.3}$$

$$P = \gamma \int_0^{2\pi} \left.\frac{\partial\zeta}{\partial\tau}\right|_{\tau=\tau_a} \mathrm{d}\sigma = 0, \tag{S.4.4}$$

$$\boldsymbol{N}_\times = \gamma \int_0^{2\pi} \left.\left(\boldsymbol{r}'\frac{\partial\zeta}{\partial\tau} - \frac{c\zeta\hat{\boldsymbol{\tau}}}{\cosh\tau - \cos\sigma}\right)\right|_{\tau=\tau_a} \mathrm{d}\sigma = \mathbf{0}, \tag{S.4.5}$$

where the particle rotation $\boldsymbol{\Omega} = \boldsymbol{\epsilon} \cdot \boldsymbol{\Omega}_\times$ must be determined as part of the solution. If the particle has an equilibrium contact angle instead of a pinned contact line, then the boundary conditions at $\tau = \tau_a$ must be modified. For cylindrical particles with 90° contact angles, we have

$$\frac{\partial\zeta}{\partial\tau} = \frac{c(\hat{\boldsymbol{\tau}} \cdot \boldsymbol{\Omega}_\times)}{\cosh\tau - \cos\sigma} \quad \text{at} \quad \tau = \tau_a, \tag{S.4.6}$$

in place of Eq. (S.4.3). For spherical particles with arbitrary contact angles, the correct boundary condition is Eq. (4.30), which when expressed in bipolar coordinates gives

$$\frac{\partial}{\partial\tau}\left(\cosh\tau\frac{\partial\zeta}{\partial\sigma}\right) - \frac{\partial}{\partial\sigma}\left(\cos\sigma\frac{\partial\zeta}{\partial\tau}\right) = 0 \quad \text{at} \quad \tau = \tau_a. \tag{S.4.7}$$

Here, we have used the identity $a = c/\sinh\tau_a$ to eliminate the particle radius a. Finally, if the slope on the outer shell is specified [Eq. (S.4.2b)], then we must add another constraint,

$$\langle\zeta\rangle_R = \frac{1}{2\pi}\int_0^{2\pi} \frac{\zeta|_{\tau=\tau_R}\sinh\tau_R}{\cosh\tau_R - \cos\sigma}\,\mathrm{d}\sigma = 0, \tag{S.4.8}$$

so that the reference height is taken to be the average height of the outer shell. Here, we have used $R = c/\sinh\tau_R$ to eliminate the shell radius R.

To calculate the force, equation (4.7) may be expressed in bipolar coordinates as

$$\boldsymbol{F} = \gamma\int_0^{2\pi} \left.\left(\frac{\partial\zeta}{\partial\sigma}\frac{\partial\zeta}{\partial\tau}\hat{\boldsymbol{\sigma}} + \left\{\left(\frac{\partial\zeta}{\partial\tau}\right)^2 - \frac{1}{2}\left[\left(\frac{\partial\zeta}{\partial\sigma}\right)^2 + \left(\frac{\partial\zeta}{\partial\tau}\right)^2\right]\right\}\hat{\boldsymbol{\tau}}\right)\right|_{\tau=\tau_a} \frac{(\cosh\tau_a - \cos\sigma)\,\mathrm{d}\sigma}{c}. \tag{S.4.9}$$

To calculate the energy, equation (S.3.10) may be expressed as

$$W = \tfrac{1}{2}\gamma\int_0^{2\pi} \zeta\left.\frac{\partial\zeta}{\partial\tau}\right|_{\tau=\tau_a}\mathrm{d}\sigma - \tfrac{1}{2}\gamma\int_0^{2\pi}\left.\left(\zeta\frac{\partial\zeta}{\partial\tau} - \zeta^{\text{ext}}\frac{\partial\zeta^{\text{ext}}}{\partial\tau}\right)\right|_{\tau=\tau_R}\mathrm{d}\sigma + \tfrac{1}{2}\pi\gamma a^2(\Omega_x^2 + \Omega_y^2), \tag{S.4.10}$$

where the components Ω_x, Ω_y of the particle rotation must be determined simultaneously with the interface height ζ. For spherical particles with equilibrium contact angles, an additional term must be added to (S.4.10) that accounts for the work needed to distort the contact line. Following the same procedure as in §S.3.2.2, we denote by W_1 the energy given by Eq.

(S.4.10). The extra energy W_2 needed to deform the contact line is then given by Eq. (S.3.51), which is straightforwardly written in bipolar coordinates:

$$W_2 = \tfrac{1}{2}\gamma \int_0^{2\pi} \frac{\tilde{\zeta}^2|_{\tau=\tau_a} \sinh \tau_a}{\cosh \tau_a - \cos \sigma}\, \mathrm{d}\sigma, \tag{S.4.11}$$

where $\tilde{\zeta} = [\zeta - U - \Omega_x y + \Omega_y (x - \xi)]|_{\tau=\tau_a}$, ξ is defined by Eq. (S.B.2) in Appendix S.B, and U, Ω_x, and Ω_y are given by Eqs. (S.4.35)-(S.4.36), below. The sum $W = W_1 + W_2$ gives the total energy for spherical particles with equilibrium contact angles.

S.4.2 Eigenfunction expansions

To find a unique solution, we must first represent ζ^{ext} and $\partial\zeta^{\mathrm{ext}}/\partial\tau$ on the outer boundary as a series of orthogonal modes:

$$\zeta^{\mathrm{ext}}|_{\tau=\tau_R} = a_0 + \sum_{n=1}^{\infty} \left[a_n \cos(n\sigma) + b_n \sin(n\sigma)\right], \tag{S.4.12}$$

$$\left.\frac{\partial \zeta^{\mathrm{ext}}}{\partial \tau}\right|_{\tau=\tau_R} = \sum_{n=1}^{\infty} n\left[c_n \cos(n\sigma) + d_n \sin(n\sigma)\right], \tag{S.4.13}$$

where the coefficients a_n, b_n, c_n, and d_n are given by

$$a_0 = \frac{1}{2\pi}\int_0^{2\pi} \zeta^{\mathrm{ext}}|_{\tau=\tau_R}\, \mathrm{d}\sigma, \tag{S.4.14a}$$

$$a_n = \frac{1}{\pi}\int_0^{2\pi} \zeta^{\mathrm{ext}}|_{\tau=\tau_R} \cos(n\sigma)\, \mathrm{d}\sigma, \qquad n = 1, 2, \ldots, \tag{S.4.14b}$$

$$b_n = \frac{1}{\pi}\int_0^{2\pi} \zeta^{\mathrm{ext}}|_{\tau=\tau_R} \sin(n\sigma)\, \mathrm{d}\sigma, \qquad n = 1, 2, \ldots, \tag{S.4.14c}$$

$$c_n = \frac{1}{n\pi}\int_0^{2\pi} \left.\frac{\partial \zeta^{\mathrm{ext}}}{\partial \tau}\right|_{\tau=\tau_R} \cos(n\sigma)\, \mathrm{d}\sigma, \qquad n = 1, 2, \ldots, \tag{S.4.14d}$$

$$d_n = \frac{1}{n\pi}\int_0^{2\pi} \left.\frac{\partial \zeta^{\mathrm{ext}}}{\partial \tau}\right|_{\tau=\tau_R} \sin(n\sigma)\, \mathrm{d}\sigma, \qquad n = 1, 2, \ldots. \tag{S.4.14e}$$

Then, we may expand the height ζ in terms of eigenfunctions of Laplace's equation:

$$\zeta = \zeta_0 + \sum_{n=1}^{\infty} \left[\left(\zeta_n^+ \mathrm{e}^{n\tau} + \zeta_n^- \mathrm{e}^{-n\tau}\right) \cos(n\sigma) + \left(\chi_n^+ \mathrm{e}^{n\tau} + \chi_n^- \mathrm{e}^{-n\tau}\right) \sin(n\sigma)\right], \tag{S.4.15}$$

where the coefficients ζ_0, ζ_n^+, ζ_n^-, χ_n^+, and χ_n^- are to be determined from the boundary conditions. Note that the force-free condition (S.4.4) is automatically satisfied by the general solution (S.4.15). Substituting Eq. (S.4.15) into (S.4.8) gives the average height of the outer shell:

$$\langle \zeta \rangle_R = \zeta_0 + \sum_{n=1}^{\infty} \mathrm{e}^{-n\tau_R} \left(\zeta_n^+ \mathrm{e}^{n\tau_R} + \zeta_n^- \mathrm{e}^{-n\tau_R}\right). \tag{S.4.16}$$

Equations (S.4.9)-(S.4.11) may also be written as double series in terms of the coefficients ζ_n^+, ζ_n^-, χ_n^+, and χ_n^-. For our purposes, we simply approximate the integrals in these expressions by numerical quadratures.

Particle rotation and torque: Since the particle rotation $\boldsymbol{\Omega}$ must also be calculated, it behooves us to expand the right-hand side of Eqs. (S.4.3) and (S.4.6) in terms of orthogonal modes:

$$\frac{c(\hat{\boldsymbol{\sigma}} \cdot \boldsymbol{\Omega}_\times)}{\cosh\tau - \cos\sigma} = -\frac{c(\hat{\boldsymbol{\tau}} \cdot \boldsymbol{\Omega})}{\cosh\tau - \cos\sigma} = 2c\sum_{n=1}^{\infty} n\mathrm{e}^{-n\tau}\left[\Omega_x \cos(n\sigma) + \Omega_y \sin(n\sigma)\right], \tag{S.4.17}$$

$$\frac{c(\hat{\boldsymbol{\tau}} \cdot \boldsymbol{\Omega}_\times)}{\cosh\tau - \cos\sigma} = \frac{c(\hat{\boldsymbol{\sigma}} \cdot \boldsymbol{\Omega})}{\cosh\tau - \cos\sigma} = 2c\sum_{n=1}^{\infty} n\mathrm{e}^{-n\tau}\left[\Omega_y \cos(n\sigma) - \Omega_x \sin(n\sigma)\right]. \tag{S.4.18}$$

The trick to obtaining these series representations is to substitute $\cosh\tau = \frac{1}{2}(\mathrm{e}^{\tau} + \mathrm{e}^{-\tau})$ and $\sinh\tau = \frac{1}{2}(\mathrm{e}^{\tau} - \mathrm{e}^{-\tau})$ and Taylor expand about the singular point $\mathrm{e}^{\tau} = \infty$, which is excluded from the domain. Then, projecting the Taylor series onto the $\cos(n\sigma)$ and $\sin(n\sigma)$ modes directly gives the series coefficients in Eqs. (S.4.17)-(S.4.18).

The Cartesian components Ω_x and Ω_y of the particle rotation must be determined by applying the torque-free condition (S.4.5). After inserting the general solution (S.4.15) into (S.4.5) and exchanging the order of integration and summation, we obtain the following series representation for the lateral torque:

$$\boldsymbol{N}_\times = -\boldsymbol{\epsilon}\cdot\boldsymbol{N} = 4\pi\gamma c\sum_{n=1}^{\infty} n\left(\zeta_n^+\hat{\boldsymbol{x}} + \chi_n^+\hat{\boldsymbol{y}}\right). \tag{S.4.19}$$

(Again, the trick is to Taylor-expand each mode about $e^\tau = \infty$ and integrate term-wise). Resolving Eq. (S.4.19) into x and y components then gives

$$N_x = 4\pi\gamma c\sum_{n=1}^{\infty} n\chi_n^+, \qquad N_y = -4\pi\gamma c\sum_{n=1}^{\infty} n\zeta_n^+. \tag{S.4.20}$$

Thus, all of the growing modes (with respect to τ) are coupled through the torque-free condition (S.4.5) such that the capillary dipole is annihilated.

Sphere "dipole" density: For spherical particles with equilibrium contact angles, the correct boundary condition is given by Eq. (S.4.7) and is independent of the particle rotation $\boldsymbol{\Omega}$. Thus, we also need to expand the terms on the left-hand side of (S.4.7) in terms of orthogonal modes. After substituting Eq. (S.4.15) into (S.4.7), the first term becomes

$$\frac{\partial}{\partial\tau}\left(\cosh\tau\frac{\partial\zeta}{\partial\sigma}\right) = \sum_{n=1}^{\infty} n\left\{-\left[\zeta_n^+e^{n\tau}(n\cosh\tau+\sinh\tau) - \zeta_n^-e^{-n\tau}(n\cosh\tau-\sinh\tau)\right]\sin(n\sigma)\right.$$
$$\left. + \left[\chi_n^+e^{n\tau}(n\cosh\tau+\sinh\tau) - \chi_n^-e^{-n\tau}(n\cosh\tau-\sinh\tau)\right]\cos(n\sigma)\right\}, \tag{S.4.21}$$

while the second term becomes

$$\frac{\partial}{\partial\sigma}\left(\cos\sigma\frac{\partial\zeta}{\partial\tau}\right) = \sum_{n=1}^{\infty} n\left\{-\left(\zeta_n^+e^{n\tau} - \zeta_n^-e^{-n\tau}\right)\left[n\sin(n\sigma)\cos\sigma + \cos(n\sigma)\sin\sigma\right]\right.$$
$$\left. + \left(\chi_n^+e^{n\tau} - \chi_n^-e^{-n\tau}\right)\left[n\cos(n\sigma)\cos\sigma - \sin(n\sigma)\sin\sigma\right]\right\}. \tag{S.4.22}$$

By making use of the trigonometric identities,

$$n\cosh\tau + \sinh\tau = \tfrac{1}{2}\left[(n-1)e^{-\tau} + (n+1)e^{\tau}\right], \tag{S.4.23a}$$
$$n\cosh\tau - \sinh\tau = \tfrac{1}{2}\left[(n+1)e^{-\tau} + (n-1)e^{\tau}\right], \tag{S.4.23b}$$

and

$$n\sin(n\sigma)\cos\sigma + \cos(n\sigma)\sin\sigma = \tfrac{1}{2}\left\{(n-1)\sin[(n-1)\sigma] + (n+1)\sin[(n+1)\sigma]\right\}, \tag{S.4.24a}$$
$$n\cos(n\sigma)\cos\sigma - \sin(n\sigma)\sin\sigma = \tfrac{1}{2}\left\{(n-1)\cos[(n-1)\sigma] + (n+1)\cos[(n+1)\sigma]\right\}, \tag{S.4.24b}$$

the previous series (S.4.21)-(S.4.22) may be rewritten as

$$\frac{\partial}{\partial\tau}\left(\cosh\tau\frac{\partial\zeta}{\partial\sigma}\right) = \tfrac{1}{2}\sum_{n=1}^{\infty} n\left(-\left\{\zeta_n^+\left[(n-1)e^{(n-1)\tau} + (n+1)e^{(n+1)\tau}\right] - \zeta_n^-\left[(n-1)e^{-(n-1)\tau} + (n+1)e^{-(n+1)\tau}\right]\right\}\sin(n\sigma)\right.$$
$$\left. + \left\{\chi_n^+\left[(n-1)e^{(n-1)\tau} + (n+1)e^{(n+1)\tau}\right] - \chi_n^-e^{-n\tau}\left[(n-1)e^{-(n-1)\tau} + (n+1)e^{-(n+1)\tau}\right]\right\}\cos(n\sigma)\right), \tag{S.4.25}$$

$$\frac{\partial}{\partial\sigma}\left(\cos\sigma\frac{\partial\zeta}{\partial\tau}\right) = \tfrac{1}{2}\sum_{n=1}^{\infty} n\left(-\left(\zeta_n^+e^{n\tau} - \zeta_n^-e^{-n\tau}\right)\left\{(n-1)\sin[(n-1)\sigma] + (n+1)\sin[(n+1)\sigma]\right\}\right.$$
$$\left. + \left(\chi_n^+e^{n\tau} - \chi_n^-e^{-n\tau}\right)\left\{(n-1)\cos[(n-1)\sigma] + (n+1)\cos[(n+1)\sigma]\right\}\right). \tag{S.4.26}$$

Equations (S.4.25)-(S.4.26) clearly show that the sphere boundary condition "scatters" the n mode to the $n-1$ and $n+1$ levels. Thus, the $n-1$, n, and $n+1$ modes are all coupled through Eq. (S.4.7) (a banded system of linear equations).

S.4.3 Solution of the series coefficients

Now we must solve for the series coefficients ζ_0, ζ_n^+, ζ_n^-, χ_n^+, and χ_n^- by applying the boundary conditions (S.4.2)-(S.4.8). For *particles with symmetrically pinned contact lines*, we obtain the following system of equations:

$$\textit{either} \qquad \zeta_{\mathcal{U}0} = a_0, \qquad \zeta_{\mathcal{U}n}^+ \mathrm{e}^{n\tau_R} + \zeta_{\mathcal{U}n}^- \mathrm{e}^{-n\tau_R} = a_n, \quad \chi_{\mathcal{U}n}^+ \mathrm{e}^{n\tau_R} + \chi_{\mathcal{U}n}^- \mathrm{e}^{-n\tau_R} = b_n \tag{S.4.27a}$$

$$\textit{or} \qquad \zeta_{\mathcal{P}0} = -\sum_{n=1}^{\infty} \mathrm{e}^{-n\tau_R}\left(\zeta_{\mathcal{P}n}^+ \mathrm{e}^{n\tau_R} + \zeta_{\mathcal{P}n}^- \mathrm{e}^{-n\tau_R}\right), \quad \zeta_{\mathcal{P}n}^+ \mathrm{e}^{n\tau_R} - \zeta_{\mathcal{P}n}^- \mathrm{e}^{-n\tau_R} = c_n, \quad \chi_{\mathcal{P}n}^+ \mathrm{e}^{n\tau_R} - \chi_{\mathcal{P}n}^- \mathrm{e}^{-n\tau_R} = d_n, \tag{S.4.27b}$$

and

$$\zeta_n^+ \mathrm{e}^{n\tau_a} + \zeta_n^- \mathrm{e}^{-n\tau_a} = -2c\Omega_y \mathrm{e}^{-n\tau_a}, \qquad \chi_n^+ \mathrm{e}^{n\tau_a} + \chi_n^- \mathrm{e}^{-n\tau_a} = 2c\Omega_x \mathrm{e}^{-n\tau_a}, \tag{S.4.28}$$

which can be derived from Eqs. (S.4.2)-(S.4.3) (it is implied that $n = 1, 2, \dots$). The solution of Eqs. (S.4.27)-(S.4.28) is

$$\zeta_{\mathcal{U}0} = a_0, \qquad \zeta_{\mathcal{P}0} = -\sum_{n=1}^{\infty}\left[c_n \mathrm{e}^{-n\tau_R}\left(\frac{\mathrm{e}^{-n(\tau_a-\tau_R)} - \mathrm{e}^{n(\tau_a-\tau_R)}}{\mathrm{e}^{-n(\tau_a-\tau_R)} + \mathrm{e}^{n(\tau_a-\tau_R)}}\right) - \frac{4c\Omega_{\mathcal{P}y}\mathrm{e}^{-n(\tau_a+\tau_R)}}{\mathrm{e}^{-n(\tau_a-\tau_R)} + \mathrm{e}^{n(\tau_a-\tau_R)}}\right], \tag{S.4.29a}$$

$$\zeta_{\mathcal{U}n}^+ = \frac{a_n \mathrm{e}^{-n\tau_a} + 2c\Omega_{\mathcal{U}y}\mathrm{e}^{-n(\tau_a+\tau_R)}}{\mathrm{e}^{-n(\tau_a-\tau_R)} - \mathrm{e}^{n(\tau_a-\tau_R)}}, \qquad \zeta_{\mathcal{P}n}^+ = \frac{c_n \mathrm{e}^{-n\tau_a} - 2c\Omega_{\mathcal{P}y}\mathrm{e}^{-n(\tau_a+\tau_R)}}{\mathrm{e}^{-n(\tau_a-\tau_R)} + \mathrm{e}^{n(\tau_a-\tau_R)}}, \tag{S.4.29b}$$

$$\zeta_{\mathcal{U}n}^- = -\frac{a_n \mathrm{e}^{n\tau_a} + 2c\Omega_{\mathcal{U}y}\mathrm{e}^{-n(\tau_a-\tau_R)}}{\mathrm{e}^{-n(\tau_a-\tau_R)} - \mathrm{e}^{n(\tau_a-\tau_R)}}, \qquad \zeta_{\mathcal{P}n}^- = -\frac{c_n \mathrm{e}^{n\tau_a} + 2c\Omega_{\mathcal{P}y}\mathrm{e}^{-n(\tau_a-\tau_R)}}{\mathrm{e}^{-n(\tau_a-\tau_R)} + \mathrm{e}^{n(\tau_a-\tau_R)}}, \tag{S.4.29c}$$

$$\chi_{\mathcal{U}n}^+ = \frac{b_n \mathrm{e}^{-n\tau_a} - 2c\Omega_{\mathcal{U}x}\mathrm{e}^{-n(\tau_a+\tau_R)}}{\mathrm{e}^{-n(\tau_a-\tau_R)} - \mathrm{e}^{n(\tau_a-\tau_R)}}, \qquad \chi_{\mathcal{P}n}^+ = \frac{d_n \mathrm{e}^{-n\tau_a} + 2c\Omega_{\mathcal{P}x}\mathrm{e}^{-n(\tau_a+\tau_R)}}{\mathrm{e}^{-n(\tau_a-\tau_R)} + \mathrm{e}^{n(\tau_a-\tau_R)}}, \tag{S.4.29d}$$

$$\chi_{\mathcal{U}n}^- = -\frac{b_n \mathrm{e}^{n\tau_a} - 2c\Omega_{\mathcal{U}x}\mathrm{e}^{-n(\tau_a-\tau_R)}}{\mathrm{e}^{-n(\tau_a-\tau_R)} - \mathrm{e}^{n(\tau_a-\tau_R)}}, \qquad \chi_{\mathcal{P}n}^- = -\frac{d_n \mathrm{e}^{n\tau_a} - 2c\Omega_{\mathcal{P}x}\mathrm{e}^{-n(\tau_a-\tau_R)}}{\mathrm{e}^{-n(\tau_a-\tau_R)} + \mathrm{e}^{n(\tau_a-\tau_R)}}. \tag{S.4.29e}$$

The constants Ω_x and Ω_y are obtained by setting the right-hand side of Eqs. (S.4.20) equal to zero, giving

$$\Omega_{\mathcal{U}x} = \frac{1}{2c}\frac{\sum_{n=1}^{\infty}\frac{nb_n\mathrm{e}^{-n\tau_a}}{\mathrm{e}^{-n(\tau_a-\tau_R)}-\mathrm{e}^{n(\tau_a-\tau_R)}}}{\sum_{n=1}^{\infty}\frac{n\mathrm{e}^{-n(\tau_a+\tau_R)}}{\mathrm{e}^{-n(\tau_a-\tau_R)}-\mathrm{e}^{n(\tau_a-\tau_R)}}}, \qquad \Omega_{\mathcal{P}x} = -\frac{1}{2c}\frac{\sum_{n=1}^{\infty}\frac{nd_n\mathrm{e}^{-n\tau_a}}{\mathrm{e}^{-n(\tau_a-\tau_R)}+\mathrm{e}^{n(\tau_a-\tau_R)}}}{\sum_{n=1}^{\infty}\frac{n\mathrm{e}^{-n(\tau_a+\tau_R)}}{\mathrm{e}^{-n(\tau_a-\tau_R)}+\mathrm{e}^{n(\tau_a-\tau_R)}}}, \tag{S.4.30a}$$

$$\Omega_{\mathcal{U}y} = -\frac{1}{2c}\frac{\sum_{n=1}^{\infty}\frac{na_n\mathrm{e}^{-n\tau_a}}{\mathrm{e}^{-n(\tau_a-\tau_R)}-\mathrm{e}^{n(\tau_a-\tau_R)}}}{\sum_{n=1}^{\infty}\frac{n\mathrm{e}^{-n(\tau_a+\tau_R)}}{\mathrm{e}^{-n(\tau_a-\tau_R)}-\mathrm{e}^{n(\tau_a-\tau_R)}}}, \qquad \Omega_{\mathcal{P}y} = \frac{1}{2c}\frac{\sum_{n=1}^{\infty}\frac{nc_n\mathrm{e}^{-n\tau_a}}{\mathrm{e}^{-n(\tau_a-\tau_R)}+\mathrm{e}^{n(\tau_a-\tau_R)}}}{\sum_{n=1}^{\infty}\frac{n\mathrm{e}^{-n(\tau_a+\tau_R)}}{\mathrm{e}^{-n(\tau_a-\tau_R)}+\mathrm{e}^{n(\tau_a-\tau_R)}}}. \tag{S.4.30b}$$

Substituting the above expressions into Eq. (S.4.15) yields the particular solution for the interface height around a particle with a symmetrically pinned contact line.

For *cylindrical particles with 90° contact angles*, Eq. (S.4.28) is replaced by

$$\zeta_n^+ \mathrm{e}^{n\tau_a} - \zeta_n^- \mathrm{e}^{-n\tau_a} = 2c\Omega_y \mathrm{e}^{-n\tau_a}, \qquad \chi_n^+ \mathrm{e}^{n\tau_a} - \chi_n^- \mathrm{e}^{-n\tau_a} = -2c\Omega_x \mathrm{e}^{-n\tau_a}. \tag{S.4.31}$$

The new coefficients are obtained by solving Eqs. (S.4.27) and (S.4.31) simultaneously, giving

$$\zeta_{\mathcal{U}0} = a_0, \qquad \zeta_{\mathcal{P}0} = -\sum_{n=1}^{\infty}\left[c_n \mathrm{e}^{-n\tau_R}\left(\frac{\mathrm{e}^{-n(\tau_a-\tau_R)} + \mathrm{e}^{n(\tau_a-\tau_R)}}{\mathrm{e}^{-n(\tau_a-\tau_R)} - \mathrm{e}^{n(\tau_a-\tau_R)}}\right) - \frac{4c\Omega_{\mathcal{P}y}\mathrm{e}^{-n(\tau_a+\tau_R)}}{\mathrm{e}^{-n(\tau_a-\tau_R)} - \mathrm{e}^{n(\tau_a-\tau_R)}}\right], \tag{S.4.32a}$$

$$\zeta_{\mathcal{U}n}^+ = \frac{a_n \mathrm{e}^{-n\tau_a} + 2c\Omega_{\mathcal{U}y}\mathrm{e}^{-n(\tau_a+\tau_R)}}{\mathrm{e}^{-n(\tau_a-\tau_R)} + \mathrm{e}^{n(\tau_a-\tau_R)}}, \qquad \zeta_{\mathcal{P}n}^+ = \frac{c_n \mathrm{e}^{-n\tau_a} - 2c\Omega_{\mathcal{P}y}\mathrm{e}^{-n(\tau_a+\tau_R)}}{\mathrm{e}^{-n(\tau_a-\tau_R)} - \mathrm{e}^{n(\tau_a-\tau_R)}}, \tag{S.4.32b}$$

$$\zeta^{-}_{\mathcal{U}n} = \frac{a_n e^{n\tau_a} - 2c\Omega_{\mathcal{U}y} e^{-n(\tau_a-\tau_R)}}{e^{-n(\tau_a-\tau_R)} + e^{n(\tau_a-\tau_R)}}, \qquad \zeta^{-}_{\mathcal{P}n} = \frac{c_n e^{n\tau_a} - 2c\Omega_{\mathcal{P}y} e^{-n(\tau_a-\tau_R)}}{e^{-n(\tau_a-\tau_R)} - e^{n(\tau_a-\tau_R)}}, \tag{S.4.32c}$$

$$\chi^{+}_{\mathcal{U}n} = \frac{b_n e^{-n\tau_a} - 2c\Omega_{\mathcal{U}x} e^{-n(\tau_a+\tau_R)}}{e^{-n(\tau_a-\tau_R)} + e^{n(\tau_a-\tau_R)}}, \qquad \chi^{+}_{\mathcal{P}n} = \frac{d_n e^{-n\tau_a} + 2c\Omega_{\mathcal{P}x} e^{-n(\tau_a+\tau_R)}}{e^{-n(\tau_a-\tau_R)} - e^{n(\tau_a-\tau_R)}}, \tag{S.4.32d}$$

$$\chi^{-}_{\mathcal{U}n} = \frac{b_n e^{n\tau_a} + 2c\Omega_{\mathcal{U}x} e^{-n(\tau_a-\tau_R)}}{e^{-n(\tau_a-\tau_R)} + e^{n(\tau_a-\tau_R)}}, \qquad \chi^{-}_{\mathcal{P}n} = \frac{d_n e^{n\tau_a} + 2c\Omega_{\mathcal{P}x} e^{-n(\tau_a-\tau_R)}}{e^{-n(\tau_a-\tau_R)} - e^{n(\tau_a-\tau_R)}}, \tag{S.4.32e}$$

where

$$\Omega_{\mathcal{U}x} = \frac{1}{2c} \frac{\sum_{n=1}^{\infty} \frac{n b_n e^{-n\tau_a}}{e^{-n(\tau_a-\tau_R)}+e^{n(\tau_a-\tau_R)}}}{\sum_{n=1}^{\infty} \frac{n e^{-n(\tau_a+\tau_R)}}{e^{-n(\tau_a-\tau_R)}+e^{n(\tau_a-\tau_R)}}}, \qquad \Omega_{\mathcal{P}x} = -\frac{1}{2c} \frac{\sum_{n=1}^{\infty} \frac{n d_n e^{-n\tau_a}}{e^{-n(\tau_a-\tau_R)}-e^{n(\tau_a-\tau_R)}}}{\sum_{n=1}^{\infty} \frac{n e^{-n(\tau_a+\tau_R)}}{e^{-n(\tau_a-\tau_R)}-e^{n(\tau_a-\tau_R)}}}, \tag{S.4.33a}$$

$$\Omega_{\mathcal{U}y} = -\frac{1}{2c} \frac{\sum_{n=1}^{\infty} \frac{n a_n e^{-n\tau_a}}{e^{-n(\tau_a-\tau_R)}+e^{n(\tau_a-\tau_R)}}}{\sum_{n=1}^{\infty} \frac{n e^{-n(\tau_a+\tau_R)}}{e^{-n(\tau_a-\tau_R)}+e^{n(\tau_a-\tau_R)}}}, \qquad \Omega_{\mathcal{P}y} = \frac{1}{2c} \frac{\sum_{n=1}^{\infty} \frac{n c_n e^{-n\tau_a}}{e^{-n(\tau_a-\tau_R)}-e^{n(\tau_a-\tau_R)}}}{\sum_{n=1}^{\infty} \frac{n e^{-n(\tau_a+\tau_R)}}{e^{-n(\tau_a-\tau_R)}-e^{n(\tau_a-\tau_R)}}}. \tag{S.4.33b}$$

Finally, for *spherical particles with arbitrary contact angles*, the boundary condition at $\tau = \tau_a$ is given by Eq. (S.4.7). After substituting Eqs. (S.4.25)-(S.4.26) into (S.4.7) and projecting the result onto each n mode, we obtain a banded system of equations,

$$\begin{aligned} &\zeta_n^+ \left[(n-1)e^{(n-1)\tau_a} + (n+1)e^{(n+1)\tau_a}\right] - \zeta_n^- \left[(n-1)e^{-(n-1)\tau_a} + (n+1)e^{-(n+1)\tau_a}\right] \\ &\quad - (n-1)\left[\zeta_{n-1}^+ e^{(n-1)\tau_a} - \zeta_{n-1}^- e^{-(n-1)\tau_a}\right] - (n+1)\left[\zeta_{n+1}^+ e^{(n+1)\tau_a} - \zeta_{n+1}^- e^{-(n+1)\tau_a}\right] = 0, \end{aligned} \tag{S.4.34a}$$

$$\begin{aligned} &\chi_n^+ \left[(n-1)e^{(n-1)\tau_a} + (n+1)e^{(n+1)\tau_a}\right] - \chi_n^- \left[(n-1)e^{-(n-1)\tau_a} + (n+1)e^{-(n+1)\tau_a}\right] \\ &\quad - (n-1)\left[\chi_{n-1}^+ e^{(n-1)\tau_a} - \chi_{n-1}^- e^{-(n-1)\tau_a}\right] - (n+1)\left[\chi_{n+1}^+ e^{(n+1)\tau_a} - \chi_{n+1}^- e^{-(n+1)\tau_a}\right] = 0, \end{aligned} \tag{S.4.34b}$$

which must be solved together with Eqs. (S.4.27). The system formed by Eqs. (S.4.27) and (S.4.34) can be solved numerically for ζ_0, ζ_n^+, ζ_n^-, χ_n^+, and χ_n^-, $n = 1, 2, \ldots, N$ by truncating the series after N modes and setting $\zeta_{N+1}^+ = \zeta_{N+1}^- = \chi_{N+1}^+ = \chi_{N+1}^- = 0$. The solution, though not conveniently written in closed analytical form, is straightforwardly obtained via a banded matrix solver. For the numerical calculation, the values of c, τ_a, and τ_R as well as the source coefficients a_n, b_n, c_n, and d_n must be supplied. The particle translation $U = (2\pi a)^{-1} \oint_{r'=a} \zeta \, ds$ and rotation $\boldsymbol{\Omega} = (\pi a^2)^{-1} \oint_{r'=a} \boldsymbol{\epsilon} \cdot (\zeta \hat{\boldsymbol{n}}) \, ds$ (which are needed for the energy calculation) may be determined after the fact by substituting the series solution (S.4.15) for ζ, yielding the relations

$$U = \zeta_0 + \sum_{n=1}^{\infty} e^{-n\tau_a} \left(\zeta_n^+ e^{n\tau_a} + \zeta_n^- e^{-n\tau_a}\right) \tag{S.4.35}$$

and

$$\Omega_x = \frac{2\sinh^2\tau_a}{c} \sum_{n=1}^{\infty} n e^{-n\tau_a} \left(\chi_n^+ e^{n\tau_a} + \chi_n^- e^{-n\tau_a}\right), \qquad \Omega_y = -\frac{2\sinh^2\tau_a}{c} \sum_{n=1}^{\infty} n e^{-n\tau_a} \left(\zeta_n^+ e^{n\tau_a} + \zeta_n^- e^{-n\tau_a}\right). \tag{S.4.36}$$

This completes the solution for the interface height for particles with (symmetrically) pinned contact lines and (cylindrical and spherical) particles with equilibrium contact angles.

S.4.4 Numerical procedure

Equations (S.4.9)-(S.4.11) may be evaluated numerically using the following procedure:

1. First, numerical values of a, R, and ξ are specified and the bipolar parameters c, τ_a, and τ_R are calculated using Eqs. (S.B.4)-(S.B.5) in Appendix S.B. The relevant geometric quantities are then given by Eqs. (S.B.6)-(S.B.10).

2. Second, the interfacial tension γ is specified (for convenience, we set $\gamma = 1$) and the curvature coefficients $\boldsymbol{K}_{\mathbf{0}}^{\text{ext}}$ and $(\nabla \boldsymbol{K}^{\text{ext}})_{\mathbf{0}}$ in Eq. (4.1) are expressed in the Cartesian basis,

$$\boldsymbol{K}_{\mathbf{0}}^{\text{ext}} = K_0 \left[(\hat{\boldsymbol{x}}\hat{\boldsymbol{x}} - \hat{\boldsymbol{y}}\hat{\boldsymbol{y}})\cos(2\alpha_0) + (\hat{\boldsymbol{x}}\hat{\boldsymbol{y}} + \hat{\boldsymbol{y}}\hat{\boldsymbol{x}})\sin(2\alpha_0)\right], \tag{S.4.37}$$

$$(\nabla \boldsymbol{K}^{\text{ext}})_{\mathbf{0}} = K_1 \left\{\left[\hat{\boldsymbol{x}}(\hat{\boldsymbol{x}}\hat{\boldsymbol{x}} - \hat{\boldsymbol{y}}\hat{\boldsymbol{y}}) - \hat{\boldsymbol{y}}(\hat{\boldsymbol{x}}\hat{\boldsymbol{y}} + \hat{\boldsymbol{y}}\hat{\boldsymbol{x}})\right]\cos(3\alpha_1) + \left[\hat{\boldsymbol{x}}(\hat{\boldsymbol{x}}\hat{\boldsymbol{y}} + \hat{\boldsymbol{y}}\hat{\boldsymbol{x}}) + \hat{\boldsymbol{y}}(\hat{\boldsymbol{x}}\hat{\boldsymbol{x}} - \hat{\boldsymbol{y}}\hat{\boldsymbol{y}})\right]\sin(3\alpha_1)\right\}, \tag{S.4.38}$$

using numerical values for the curvature amplitudes K_0, K_1 and phase angles α_0, α_1. Substituting Eqs. (S.4.37)-(S.4.38) and $\boldsymbol{r} = x\hat{\boldsymbol{x}} + y\hat{\boldsymbol{y}}$ into (4.1) then gives the external height ζ^{ext} in terms of the Cartesian coordinates (x, y). This expression is then rewritten in terms of the bipolar coordinates (σ, τ) using the transformation rules (S.B.3) in Appendix S.B.

3. Third, the series coefficients a_n, b_n, c_n, and d_n appearing in Eqs. (S.4.12)-(S.4.13) are calculated by applying numerical quadratures to Eqs. (S.4.14).

4. Fourth, the series coefficients ζ_0, ζ_n^+, ζ_n^-, χ_n^+, and χ_n^- appearing in Eq. (S.4.15) are calculated using Eqs. (S.4.29)-(S.4.30) (for particles with symmetrically pinned contact lines), (S.4.32)-(S.4.33) (for cylindrical particles with 90° contact angles), or the numerical solution of the banded system (S.4.27) and (S.4.34) (for spherical particles with arbitrary contact angles). The interface height ζ and its derivatives $\partial\zeta/\partial\sigma$, $\partial\zeta/\partial\tau$ are then evaluated at the boundaries $\tau = \tau_a$ and τ_R.

5. Finally, the boundaries $\tau = \tau_a$ and τ_R are discretized and Eqs. (S.4.9)-(S.4.11) are approximated using numerical quadratures [Eq. (S.4.11) is only needed for spherical particles with equilibrium contact angles].

The numerical results for $\boldsymbol{F}$ and W are plotted in Figs. S.5-S.10 alongside the approximate results obtained via the method of reflections. For all of the calculations reported here, we set $K_0 = K_1 = 1$ and $\alpha_0 = \alpha_1 = 0$ in Eqs. (S.4.37)-(S.4.38).

Appendices

S.A Vector harmonics in two dimensions

The fundamental solution of Laplace's equation in 2D is $\log r$,

$$\nabla^2 \log r = 0. \tag{S.A.1}$$

Successive gradients of $\log r$ give the decaying solutions of Laplace's equation, $\boldsymbol{\nabla}^n \log r$ with $n = 1, 2, \ldots$. The first few decaying harmonics are

$$\left.\begin{aligned} \boldsymbol{\nabla} \log r &= \frac{\boldsymbol{r}}{r^2}, \\ \boldsymbol{\nabla\nabla} \log r &= -\frac{2\boldsymbol{rr} - r^2\boldsymbol{\delta}}{r^4}, \\ \boldsymbol{\nabla\nabla\nabla} \log r &= \frac{8\boldsymbol{rrr} - 2r^2[\boldsymbol{r\delta} + \boldsymbol{\delta r} + (\boldsymbol{r\delta})^{\top}]}{r^6}, \\ &\ \ \vdots \end{aligned}\right\} \tag{S.A.2}$$

The decaying harmonics (S.A.2), with $\boldsymbol{r}$ replaced by $\boldsymbol{r}'$, are used to construct reflected modes from the particle boundary $r' = a$. Similarly, the growing solutions of Laplace's equation are given by $r^{2n}\boldsymbol{\nabla}^n \log r$ with $n = 1, 2, \ldots$. The first few growing harmonics are

$$\left.\begin{aligned} r^2\boldsymbol{\nabla} \log r &= \boldsymbol{r}, \\ r^4\boldsymbol{\nabla\nabla} \log r &= -(2\boldsymbol{rr} - r^2\boldsymbol{\delta}), \\ r^6\boldsymbol{\nabla\nabla\nabla} \log r &= 8\boldsymbol{rrr} - 2r^2[\boldsymbol{r\delta} + \boldsymbol{\delta r} + (\boldsymbol{r\delta})^{\top}], \\ &\ \ \vdots \end{aligned}\right\} \tag{S.A.3}$$

The growing harmonics (S.A.3) are used to construct reflected modes from the shell boundary $r = R$.

It is straightforward to express the decaying harmonics with respect to the particle-centered position $\boldsymbol{r}' = \boldsymbol{r} - \boldsymbol{\xi}$ in terms of the shell-centered position $\boldsymbol{r}$, by Taylor-expanding $\log r'$ about $\boldsymbol{\xi} = \boldsymbol{0}$:

$$\begin{aligned} \log r' &= \log |\boldsymbol{r} - \boldsymbol{\xi}| \\ &= \sum_{n=0}^{\infty} \frac{(-\boldsymbol{\xi} \cdot \boldsymbol{\nabla})^n}{n!} \log r \\ &= \log r - \boldsymbol{\xi} \cdot \boldsymbol{\nabla} \log r + \frac{1}{2}\boldsymbol{\xi\xi} : \boldsymbol{\nabla\nabla} \log r + \cdots \\ &= \log r - \frac{\boldsymbol{r} \cdot \boldsymbol{\xi}}{r^2} - \frac{1}{2}\left(\frac{2(\boldsymbol{r} \cdot \boldsymbol{\xi})^2 - r^2\xi^2}{r^4}\right) + \cdots . \end{aligned} \tag{S.A.4}$$

The series converges so long as $\xi < r$. Similar expressions for the higher-order decaying harmonics are obtained by taking successive gradients of Eq. (S.A.4) and exploiting the spatial invariance of the gradient, $\boldsymbol{\nabla}' = \boldsymbol{\nabla}$. The addition theorem (S.A.4) is used to represent reflected modes from the particle boundary $r' = a$ on the shell boundary $r = R$.

Another useful identity involves the derivative of a harmonic with respect to r. For some nth-order decaying harmonic, we can prove that

$$\frac{\boldsymbol{r}}{r} \cdot \boldsymbol{\nabla\nabla}^n \log r = -\frac{n}{r}\boldsymbol{\nabla}^n \log r, \qquad n = 1, 2, \ldots . \tag{S.A.5}$$

Similarly, for some nth-order growing harmonic,

$$\frac{\boldsymbol{r}}{r} \cdot \boldsymbol{\nabla}(r^{2n}\boldsymbol{\nabla}^n \log r) = \frac{n}{r}(r^{2n}\boldsymbol{\nabla}^n \log r), \qquad n = 1, 2, \ldots . \tag{S.A.6}$$

Thus, the eigenfunctions of the operator $\boldsymbol{r} \cdot \boldsymbol{\nabla} = r(\partial/\partial r)$ are the decaying and growing harmonics of order n, with eigenvalues given by $-n$ and n, respectively. Equations (S.A.5)-(S.A.6) are used to reverse the sign of reflected modes when alternating between Dirichlet and Neumann conditions on either the particle or shell boundaries.

S.B Transformation to bipolar coordinates

We choose the Cartesian coordinates (x, y) such that the center of the shell and particle are aligned at $y = 0$ and the particle is positioned to the left of the shell's center, with the x-axis directed from left to right. In this basis, we define $\boldsymbol{r} = x\hat{\boldsymbol{x}} + y\hat{\boldsymbol{y}}$ as the position measured from the shell's center and $\boldsymbol{r}' = x'\hat{\boldsymbol{x}} + y'\hat{\boldsymbol{y}}$ as the position measured from the particle's center, where the coordinates (x, y) and (x', y') are related by

$$x - x_0 = x' - x_0', \qquad y = y'. \tag{S.B.1}$$

The particle's position relative to the shell's center is then given by $\boldsymbol{\xi} = \boldsymbol{r} - \boldsymbol{r}' = \xi\hat{\boldsymbol{x}}$, where

$$\xi = x_0 - x_0'. \tag{S.B.2}$$

The bipolar-symmetry axis is defined at the horizontal position $x = x_0$ (in shell-centered coordinates) or $x' = x_0'$ (in particle-centered coordinates).

The transformation from Cartesian coordinates (x, y) to bipolar coordinates (σ, τ) is defined as

$$x = x_0 + \frac{c \sinh\tau}{\cosh\tau - \cos\sigma}, \qquad y = \frac{c\sin\sigma}{\cosh\tau - \cos\sigma}, \tag{S.B.3}$$

where $0 \le \sigma \le 2\pi$, $0 \le \tau < \infty$, and c is related to a and R by

$$c = \sqrt{x_0^2 - R^2} = \sqrt{x_0'^2 - a^2}. \tag{S.B.4}$$

The curves $\tau = \tau_a$ and $\tau = \tau_R$ (where $0 < \tau_R < \tau_a < \infty$) define the particle and shell boundaries, respectively, and are given by

$$\tau_a = \frac{1}{2}\log\left(\frac{x_0 - c}{x_0 + c}\right), \qquad \tau_R = \frac{1}{2}\log\left(\frac{x_0' - c}{x_0' + c}\right). \tag{S.B.5}$$

Fig. S.11 illustrates the bipolar coordinates (σ, τ). A few remarks are in order:

- The isocontours of σ (blue curves in Fig. S.11) form a family of circular arcs centered at $x = x_0$, $y = c\cot\sigma$ (the bipolar-symmetry axis) and passing through the limiting point $x = x_0 + c$, $y = 0$ (the center of the particle). For values of $\sigma < \pi$, the circular arc lies in the upper half plane; for values of $\sigma > \pi$, the arc lies in the lower half plane. The value $\sigma = 0$ (2π) generates a circular arc of infinite radius centered at $y = \infty$. When $\sigma = \pi$, the circles degenerate to lines on the x-axis connecting the bipolar-symmetry axis to the center of the particle. The value $\sigma = \pi/2$ $(3\pi/2)$ is a semi-circle of radius c located in the upper (lower) half plane and whose center is located at the origin.
- The isocontours of τ (red curves in Fig. S.11) form a family of non-intersecting circles of radius $c/\sinh\tau$ whose centers are located at $x = x_0 + c\coth\tau$, $y = 0$. For values of $\tau > 0$, the circle lies to the right of $x = x_0$. The value $\tau = 0$ generates a circle of infinite radius centered at $x = \infty$, $y = 0$. When $\tau = \infty$, the circles degenerate to points located at $x = x_0 + c$, $y = 0$ (the center of the particle).

Several other geometric quantities will be useful to define. These include the Cartesian basis vectors $\hat{\boldsymbol{x}}$ and $\hat{\boldsymbol{y}}$ expressed in terms of the bipolar basis vectors $\hat{\boldsymbol{\sigma}}$ and $\hat{\boldsymbol{\tau}}$:

$$\begin{pmatrix} \hat{\boldsymbol{x}} \\ \hat{\boldsymbol{y}} \end{pmatrix} = \frac{1}{\cosh\tau - \cos\sigma} \begin{pmatrix} -\sin\sigma\sinh\tau & 1 - \cos\sigma\cosh\tau \\ -1 + \cos\sigma\cosh\tau & -\sin\sigma\sinh\tau \end{pmatrix} \begin{pmatrix} \hat{\boldsymbol{\sigma}} \\ \hat{\boldsymbol{\tau}} \end{pmatrix}, \tag{S.B.6}$$

with the inverse relations,

$$\begin{pmatrix} \hat{\boldsymbol{\sigma}} \\ \hat{\boldsymbol{\tau}} \end{pmatrix} = \frac{1}{\cosh\tau - \cos\sigma} \begin{pmatrix} -\sin\sigma\sinh\tau & -1 + \cos\sigma\cosh\tau \\ 1 - \cos\sigma\cosh\tau & -\sin\sigma\sinh\tau \end{pmatrix} \begin{pmatrix} \hat{\boldsymbol{x}} \\ \hat{\boldsymbol{y}} \end{pmatrix}. \tag{S.B.7}$$

The differential arc length ds on an isocontour τ = constant is given by

$$\mathrm{d}s = \frac{c\,\mathrm{d}\sigma}{\cosh\tau - \cos\sigma}. \tag{S.B.8}$$

Finally, we may define the gradient

$$\boldsymbol{\nabla} = \hat{\boldsymbol{x}}\frac{\partial}{\partial x} + \hat{\boldsymbol{y}}\frac{\partial}{\partial y} = \frac{\cosh\tau - \cos\sigma}{c}\left(\hat{\boldsymbol{\sigma}}\frac{\partial}{\partial\sigma} + \hat{\boldsymbol{\tau}}\frac{\partial}{\partial\tau}\right) \tag{S.B.9}$$

and the Laplacian

$$\nabla^2 = \boldsymbol{\nabla}\cdot\boldsymbol{\nabla} = \frac{\partial^2}{\partial x^2} + \frac{\partial^2}{\partial y^2} = \frac{(\cosh\tau - \cos\sigma)^2}{c^2}\left(\frac{\partial^2}{\partial\sigma^2} + \frac{\partial^2}{\partial\tau^2}\right). \tag{S.B.10}$$

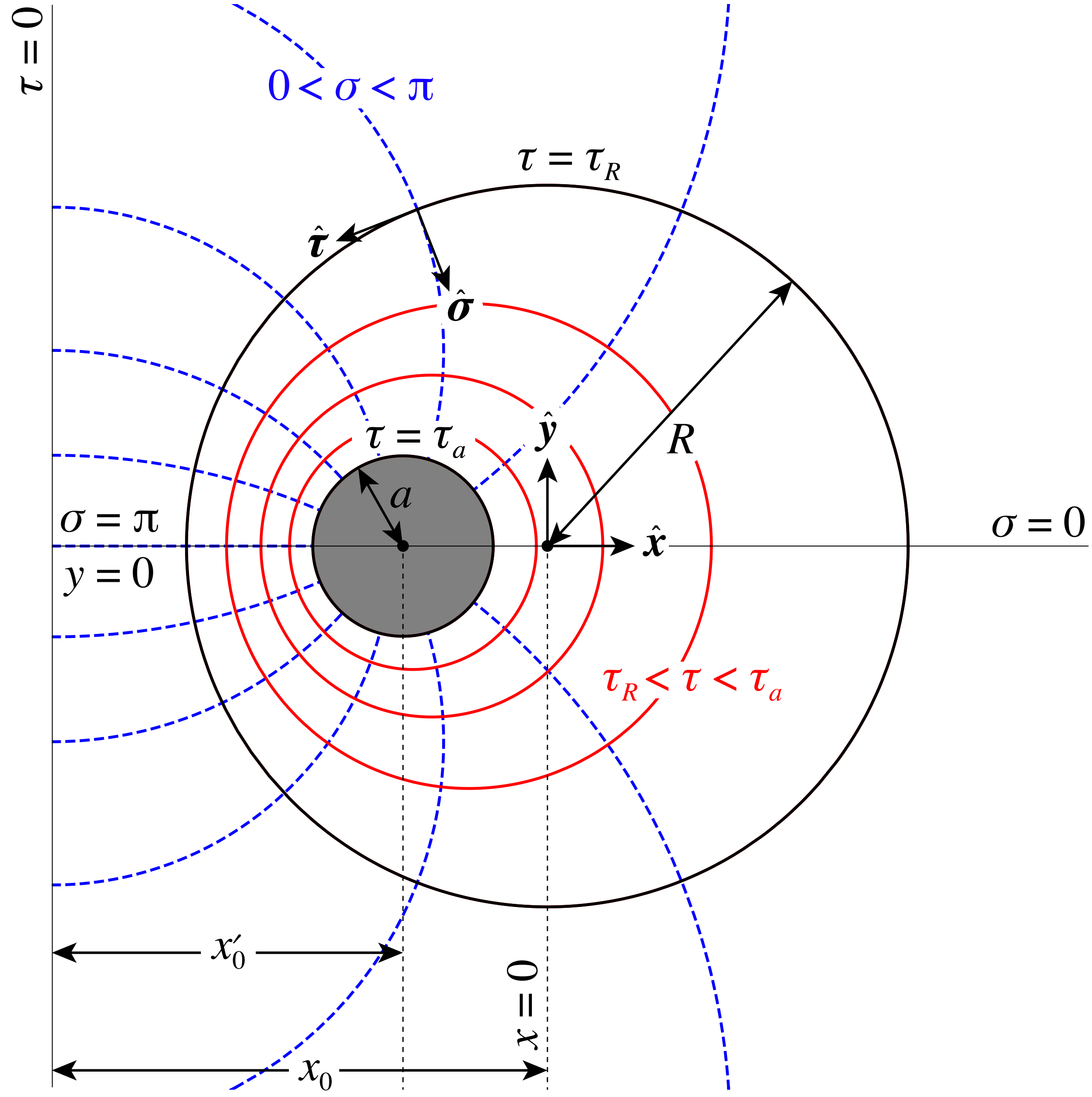

Figure S.11 Isocontours of the bipolar coordinates σ and τ.

References

[1] M. Smoluchowski, *Bull. Acad. Sci. Cracovie A*, 1911, **1**, 28–39.

[2] G. M. Golusin, *Math. Sb.*, 1934, **41**, 246–276.

[3] J. Happel and H. Brenner, *Low Reynolds Number Hydrodynamics*, Springer, 1965.

[4] J. H. C. Luke, *SIAM J. Appl. Math.*, 1989, **49**, 1635–1651.

[5] H. Liu and H. H. Bau, *Phys. Fluids*, 2004, **16**, 1217–1228.
